# Multiproxy evidence of millet reliance and selective dietary change during iron age transformation in Central Europe

**DOI:** 10.1038/s41598-025-25274-z

**Published:** 2025-11-21

**Authors:** Alžběta Danielisová, Mária Hajnalová, Adéla Pokorná, Petr Kočár, Samuel Kertés, Daniel Bursák, Kateřina Pachnerová Brabcová, Zdeněk Tvrdý, Tereza Šálková, Veronika Komárková, Ivo Světlík

**Affiliations:** 1 Czech Academy of Sciences, Institute of Archaeology, Prague, Prague, Czech Republic; 2 Constantine the Philosopher University, Nitra, Slovakia; 3 Czech Academy of Sciences, Nuclear Physics Institute, Prague, Czech Republic; 4 Moravian Museum, Brno, Czech Republic; 5 University of South Bohemia in České Budějovice, České Budějovice, Czech Republic

**Keywords:** Archaeology, Cultural evolution, Agroecology, Environmental economics, Evolutionary ecology, Stable isotope analysis

## Abstract

This study integrates archaeobotanical and stable isotopic data to investigate dietary adaptations and crop use in past Central European societies during later prehistory (500–0 BCE), a period marked by growing social complexity and technological innovation that approximates sub-modern population structures. Using a multi-proxy approach, we analyse supra-regional datasets of plant macro-remains and human bone collagen from Bohemia, Moravia, and Slovakia, focusing on millet, the only C₄ crop cultivated in preindustrial temperate Europe, which serves as a distinct isotopic marker for tracing subsistence shifts. Archaeobotanical evidence, assessed through minimum number of individuals (MNI), ubiquity, and Representativeness Index, shows that millet cultivation was largely influenced by local environmental conditions, with no consistent temporal trend across regions. In contrast, stable carbon isotope data reveal a systemic dietary shift towards higher δ13C values during the third century BCE, coinciding with technological advances and increasing socio-economic complexity. This new trend, however, was not applied consistently: its intensity varied across sites and social groups, reflecting flexible and context-specific subsistence strategies. Non-elite individuals exhibited more pronounced shifts, while elite groups maintained more conservative, C₃-based diets. By demonstrating broad patterns alongside local variability, this study highlights the flexibility and adaptive capacity of past food production. The findings underscore the importance of integrating both regional and local scales in multi-proxy analyses and provide insights relevant to modern contexts facing globalised yet ecologically diverse agricultural challenges.

**Supplementary Information:**

The online version contains supplementary material available at 10.1038/s41598-025-25274-z.

## Introduction

The Late Iron Age, or La Tène period (500–0 BCE), in Central Europe was marked by major societal transformations, culminating towards an end of the third century BCE^1^. In this period, the evidence points to the rise of large, nucleated settlements focused on craft specialisation, intensification. Integration of agglomerations into expansive supra-regional trade and communication networks brought in innovations in traditional technologies such as metalworking^2^, new materials—glass^3^, leaded bronze, and silver^2,4^, along with new decorative styles^5^ and ritual practices^1^. The introduction of coinage^6^ shaped the nature of trade relations, especially with the Mediterranean, but also within wider Transalpine area. By the late third century BCE, socio-economic structures had reached a level of complexity broadly comparable to pre-industrial or traditional modern societies. An integral part of the transformation was also a development of agricultural strategies. The traditionally cultivated crop spectrum was quite diverse, and varied regionally, with cereals typically dominated by barley and wheats ^7–11^. Archaeobotanical and osteological analyses from agglomerations noticed shifts toward more productive systems capable of supporting denser populations and growing trade^10,12^. A notable trend was the increasing reliance on millet as one of the staple crops, though its occurrence varied regionally as well as chronologically^10,13–15^.

Understanding how past communities adapted their food systems to environmental and societal challenges is essential for informing modern sustainability strategies. Millet, in particular, and its varying use across different periods and environments has proven valuable for examining broader societal dynamics, such as migration, production intensification, and social differentiation, that underlie agricultural change^16–20^. However, interpreting these variations remains challenging without sufficient contextual and supporting evidence. Traditionally, past crop use is studied through archaeobotanical macro-remains, which provide insight into dietary patterns and plant food choices shaped by environmental or cultural factors^21^. Stable isotope analysis, particularly δ^13^C in human collagen, offers an independent proxy, reflecting the balance of C₃ and C₄ plants (or marine inputs) in past human and animal diets^22,23^. C₃ crops (e.g. wheat, barley, legumes), dominating mesic woodland biomes^4,8^, typically show δ^13^C values from –37‰ to –20‰, influenced by factors like aridity or the canopy effect^24,25^. C₄ plants, on the other hand, such as millets, are typical for tropical grassland or savannah biomes^19,26^ and range from –15‰ to –10‰ in modern crops^27^, and around –12.7‰ to –11.4‰ in fossil specimens from Europe^13,19^. C₄ plants are more tolerant of arid conditions, displaying greater resilience and adaptability to water availability, and have shorter growing seasons, making them resilient in marginal environments^18,28,29^. In pre-industrial mid-latitude Europe, millets, specifically *Panicum miliaceum* and *Setaria italica,* were the only C₄ crops widely cultivated^30^. Their distinctive isotopic ‘fingerprint´ allows for reliable identification in diet. For C₄ signals to appear in collagen, millet-derived protein must make up at least 20% of the diet^31^. Given a trophic enrichment factor for carbon of up to 1‰^32^, a δ^13^C collagen threshold of − 18‰ (VPDB) is conventionally used to indicate substantial millet intake ^13,18,19,33–36^. Values near this mark suggest a mixed C₃/C₄ diet, while higher values reflect C₄ dominance^18,37,38^.

Recent scholarship has explored the cultural and economic roles of millet across Eurasia^18,20,39,40^, where its spread during the Late Bronze Age (ca. 1600–1200 BCE)^20,36,38,41–47^ has been framed as an episode of ‘food globalisation’—a reflection of intensified cultural connectivity and innovation^18,40,43,48–50^. Consequently, millet emerged as a symbol of innovation and adaptability, with its cultivation considered a critical driver of societal transformations and agricultural advances that enhanced subsistence stability in prehistoric communities^18,19^. It became a dietary mainstay across many regions, as shown by both archaeobotanical and isotopic evidence^7,18,36,41,51–54^, though its prominence declined in some areas after the Bronze Age^7,36,38^, arguably due to climate change^16^. In later periods, millet periodically resurfaced in specific socio-cultural contexts^13,34,35,41,55,56^. Besides direct link to climate fluctuations in certain periods^16,57^, integration of millet into agricultural strategies appears to reflect distinct socio-economic settings or culturally mediated preferences. For example, millet held a central role in Early Medieval agricultural systems of Southern^58–61^ and Eastern Europe^46,62,63^, while remaining largely absent among Germanic communities in Central and Western Europe^50,64–66^.

Unlike the Bronze Age, when millet first appeared as a novel crop, the La Tène period allows for the examination of shifts within an already established crop spectrum. This context enables a more detailed assessment of how environmental and socio-economic factors influenced food production, in ways that are arguably closer to more recent historical patterns. Regional and temporal fluctuations in millet use underscore its value as a proxy for agricultural resilience and socio-economic adaptation. When viewed across long timespans, isotopic shifts in human diets can be aligned with archaeobotanical trends to reconstruct nuanced cultivation and consumption strategies. This dual approach, when applied at the highest achievable temporal, spatial, and social resolution, offers key insights into how environmental, technological, and cultural forces shaped past subsistence systems, with meaningful implications for food security in today’s globalised yet ecologically diverse world.

**Fig. 1:**
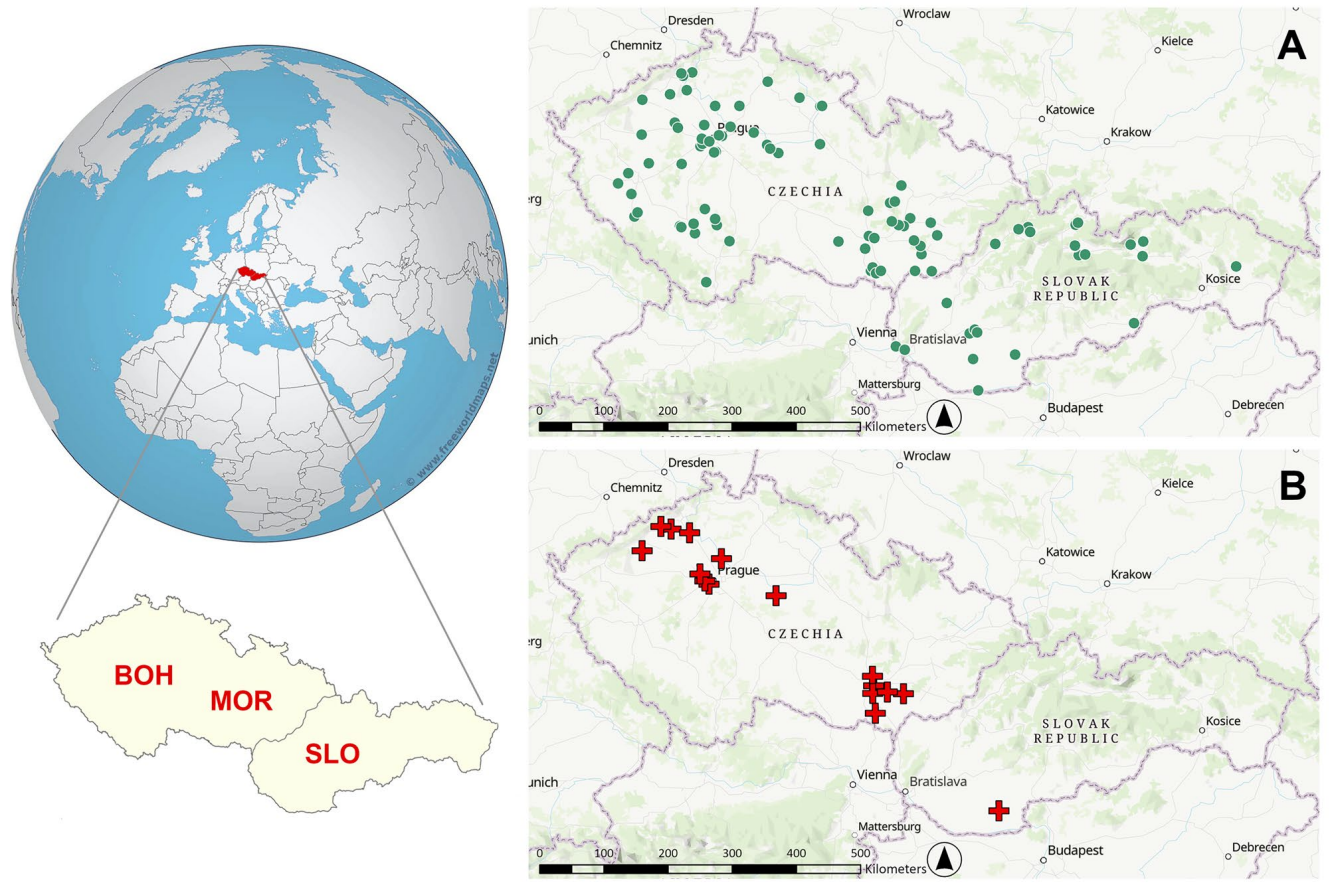
Map of the area under study (“BOH”–Bohemia, “MOR”–Moravia, “SLO”–Slovakia) with Iron Age sites with botanical (**A**) and stable isotope (**B**) data. The lists of archaeological contexts (sites) and associated datasets are provided in Tables S4 and S9, and Fig. S3 and S4. (source maps: the Earth created with MapChart.net, CC BY-SA 4.0; https://creativecommons.org/licenses/by-sa/4.0/; basemaps in A and B insets: World Topographic Map by © Esri and NASA, NGA, USGS, GUGiK, ŠOP SR, Esri TomTom, Garmin, FAO, METI/NASA; graphic by A.D.).

In this study, the significance of millet within late Iron Age agriculture is being evaluated using a multi-proxy approach of combined analysis of archaeobotanical and isotopic evidence. This multi-proxy approach is applied here for the first time, made possible by the availability of sufficiently robust and analytically comparable datasets from three regions in Central Europe —Bohemia, Moravia, and Slovakia (Fig. 1). Settlement-based archaeobotanical data were classified into distinct chronological phases to align with stable isotope evidence from human and animal remains (see Methods). Previous research identified regional variation in crop use, with barley, spelt wheat, free-threshing wheat, einkorn, and millet forming the primary agricultural spectrum^8^, with proportions influenced by geography and altitude^9,10,14,15,51^. Isotopic data, available for ca. 400–200 BCE, suggest a general reliance on C₃ crops^33^. However, several sites show a marked shift towards higher δ^13^C values during the third century BCE^56^, indicating increased millet consumption. Comparable trends have been observed in Switzerland^13,67^ and Italy^37^, but broader synthetic evaluations remain lacking.

To address this, we analyse new and previously published stable isotope data from burials contextualised by grave goods and demographic information. Statistical evaluation of both archaeobotanical and isotopic datasets, at the highest resolution permitted by the archaeological record, seeks to identify correlating temporal, geographical, and social patterns. Specifically, we test whether the isotopic shift in the third century BCE corresponds with a systemic increase in millet cultivation across the study region. The results are discussed in relation to changing food production strategies and the socio-economic organisation of La Tène societies undergoing societal transformation. By comparing production trends with local constraints, we explore how rural communities negotiated supra-regional processes while adapting to specific ecological and social settings. Finally, we address methodological considerations in integrating archaeobotanical data with dietary patterns inferred from stable isotope analysis.

## Results

### Archaeobotanical analysis

The data clearly demonstrate that millet is a relatively constant crop throughout the analysed period (Table S1, Fig. S5, S9). Its occurrence and abundance slightly fluctuate in time, and it is more common at sites in the eastern part of the study area.

**Fig. 2:**
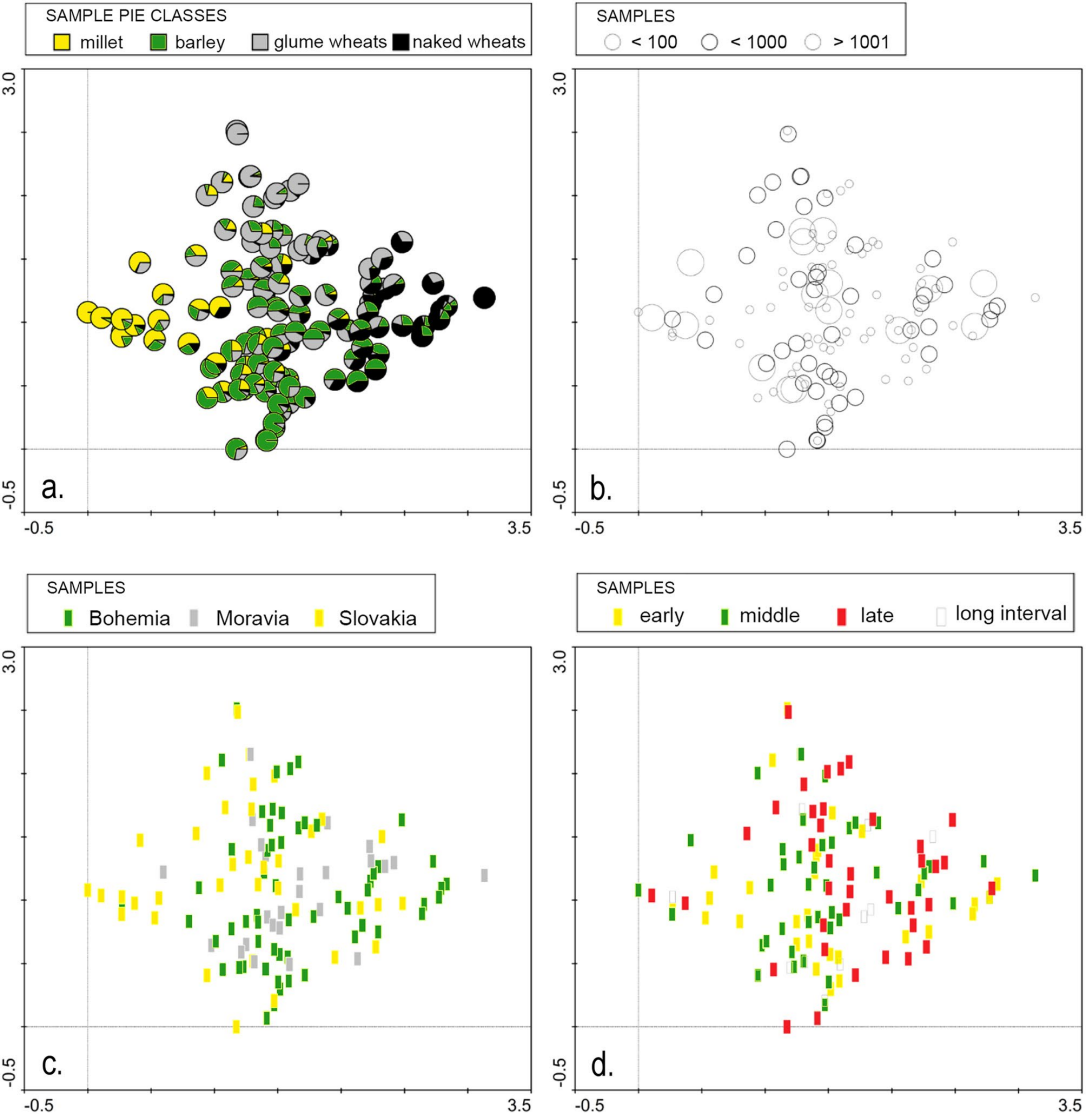
DCA plot of cereal crop assemblages dated to the La Tène period, showing cereal proportions (**a**), determined cereal finds (**b**), regional distribution (**c**), and chronological phase (**d**), (Graphic by M.H.).

The Detrended Correspondence Analysis (DCA) performed on various cereal data matrices (original, transformed, and reduced) did not reveal any definitive temporal or regional trends. In the DCA plot based on percentage values (Fig. 2), millet-rich samples were associated with low positive values on the horizontal axis (left side of the scatter), whereas free-threshing wheat-rich samples clustered at the opposite positive end. Coding samples by region and dating did not demonstrate clear separations or trends, although millet-rich samples were primarily associated with Slovakia and, to a certain extent, also Moravia.

The statistical testing using parametric one-way ANOVA was conducted using two matrices; one based on ubiquity, representing the frequency of millet occurrence across sites, and the other on percentage, calculated as the minimum number of individual (MNI) millet remains per site in relation to the overall crop spectrum. In each analysis the results are based on testing four matrices in total: two based on the original data and two on the modelled data. All the results and *p* values are listed in Table 1.

**Table 1:** Results of a series of one-way ANOVA tests with post-hoc Tukey’s Honest Significant Difference and Bonferroni-corrected tests run on various matrices of *Panicum* data.

Analysis run no	1	2	3	4	5	6	7	8	9	10	11	12	13	14	15	16	17	18
Data	Ubiquity								Percentage									
Observed	Phase				Region				Phase				Region					
Values / Matrix	Original		Modeled		Original		Modeled		> 50 mni		> 30 mni	All	> 50 mni			> 30 mni	All	All
Inclusion rule	Well dated	Dated	Well dated	Dated	Well dated	Dated	Well dated	All	Well dated	Dated	Dated	Dated	Well dated	Dated	All	Dated	Dated	All
Observations	36/35/39	38/44/41	36/35/39	38/44/41	60/23/27	63/24/36	60/23/27	68/27/41	23/20/20	23/26/22	25/32/28	38/43/42	36/11/16	37/12/22	39/13/27	43/17/26	63/24/36	71/27/45
df between groups	2	2	2	2	2	2	2	2	2	2	2	2	2	2	2	2	2	2
df within groups	107	120	107	120	107	120	107	133	60	68	82	120	60	68	76	83	120	140
F-statistics	3.66	3.21	3.93	2.67	4.41	8.54	5	11.32	0.109	0.4	0.36	1.38	2.44	2.6	2.39	1.99	6.32	5.61
p-value	**0.029**	0.043	**0.023**	0.07	**0.014**	**0.0003**	**0.008**	**0.0000**	0.89	0.96	0.71	0.26	0.09	0.08	0.09	0.14	**0.002**	**0.005**
Effect size*: ω2	0.046		0.051		0.058	0.11	0.07	0.13									0.079	0.061
Mean 1 (Early LT/Czechia)	**0.19**		**0.17**		**0.16**	**0.16**	**0.12**	**0.16**									**7.3**	**6.76**
Mean 2 (Middle LT/Moravia)	**0.36**		**0.33**		0.29	0.3	**0.33**	0.28									12.3	11.3
Mean 3 (Late LT/Slovakia)	**0.18**		0.22		**0.37**	**0.43**	**0.34**	**0.46**									**23.1**	**20.1**
SD of mean 1 (Early LT/Czechia)	0.05		**0.04**		**0.03**	**0.03**	**0.03**	**0.31**									**1.85**	**1.68**
SD of mean 2 (Middle LT/Moravia)	**0.06**		**0.05**		0.06	0.06	**0.06**	0.06									3.18	2.89
SD of mean 3 (Late LT/Slovakia)	**0.04**		0.04		**0.08**	**0.07**	**0.06**	**0.07**									**5.29**	**4.56**
Tukey HSD* p*-value 1	0.045 (2vs3)		0.018		0.015	0.001	0.02 (1vs3)	0.001									0.002	0.003
Tukey HSD* p*-value 2							0.049 (1vs2)											
Benferroni *p*-value	0.048 (1vs2)		0.02		0.016	0.002	0.03 (1vs3)	0.0000									0.001	0.003

Bold values represent significant results.

*Tresholds: 0.1 small; 0.059 medium; 0138 large.

The results on ‘ubiquity’ data, where data reduction was based on dating accuracy of sites, revealed statistically significant chronological differences either between the early (500–330 BCE) and the middle (330–180 BCE) phase or between the middle or the late (180 – 0 BCE/CE) phase, depending on which matrix was tested. Testing for geographical variability revealed statistically significant differences in all matrices used. Post hoc tests revealed that the variation lay between Bohemia and Slovakia, while in the case of modelled data, differences were also observed between Bohemia and Moravia. In summary, the analysis of ubiquity data indicates that geographical differences have a stronger influence on the patterns observed than chronological variation. *Panicum* is more frequently found in samples from Slovakia, and to a certain extent also from Moravia, compared to Bohemia. However, from the statistical point of view, the frequency or abundance of millet does not significantly change over established chronological phases within the individual regions. The absence of chronological variation when more sites are included suggests that unrecognised biases, such as uneven sample distribution, may be affecting the results.

The results of the one-way ANOVA on ‘percentages’ data, where data reduction was based both on MNI values (> 30 and 50 determined cereal finds, respectively) and dating accuracy, revealed no significant chronological differences suggesting that the proportion of millet relative to other cereal crops does not change significantly over time when the whole regions are analysed. Statistically significant differences between the regions were observed only when unreduced data were tested, however, these results are likely biased due to the inclusion of samples with low find counts. Consequently, it is likely that the percentage or abundance of *Panicum* does not differ significantly across regions when using the reduced data matrices. In general, while geographical patterns emerge more clearly in ubiquity data, the percentage-based analysis suggests that regional differences in millet abundance are not statistically significant. For percentage data, the lack of clear trends may stem from the relative nature of percentages, which are influenced by sample size. Percentages do not differentiate between actual changes in millet abundance and apparent changes caused by varying proportions of other cereals.

In conclusion, the statistical analysis of archaeobotanical data indicates minimal or no statistically significant difference in the ubiquity—frequency of occurrence—of millet between the individual chronological phases. On the other hand, a geographical gradient is evident, with millet appearing more frequently in Slovakia in the East than in Bohemia in the West, while Moravia occupies an intermediate position. No statistically significant differences were observed in the proportions of millet within site assemblages, as millet-rich and millet-poor samples were present across all chronological phases and throughout the entire geographical gradient. Future studies should address potential biases and use more refined metrics, such as seed density per litre of sediment, to improve data reliability and resolution. Additionally, site-specific or more detailed sub-regional resolution is essential, as demonstrated by spatial plots for each site (Fig. S5–S12). At this refined scale, geographical and chronological variation becomes evident, revealing differences on a local scale. The ubiquity data indicate a peak in millet presence during the middle period, with percentage values highlighting this intensity particularly in central Bohemia and Moravia, and its continuation into the late period in Slovakia.

**Fig. 3:**
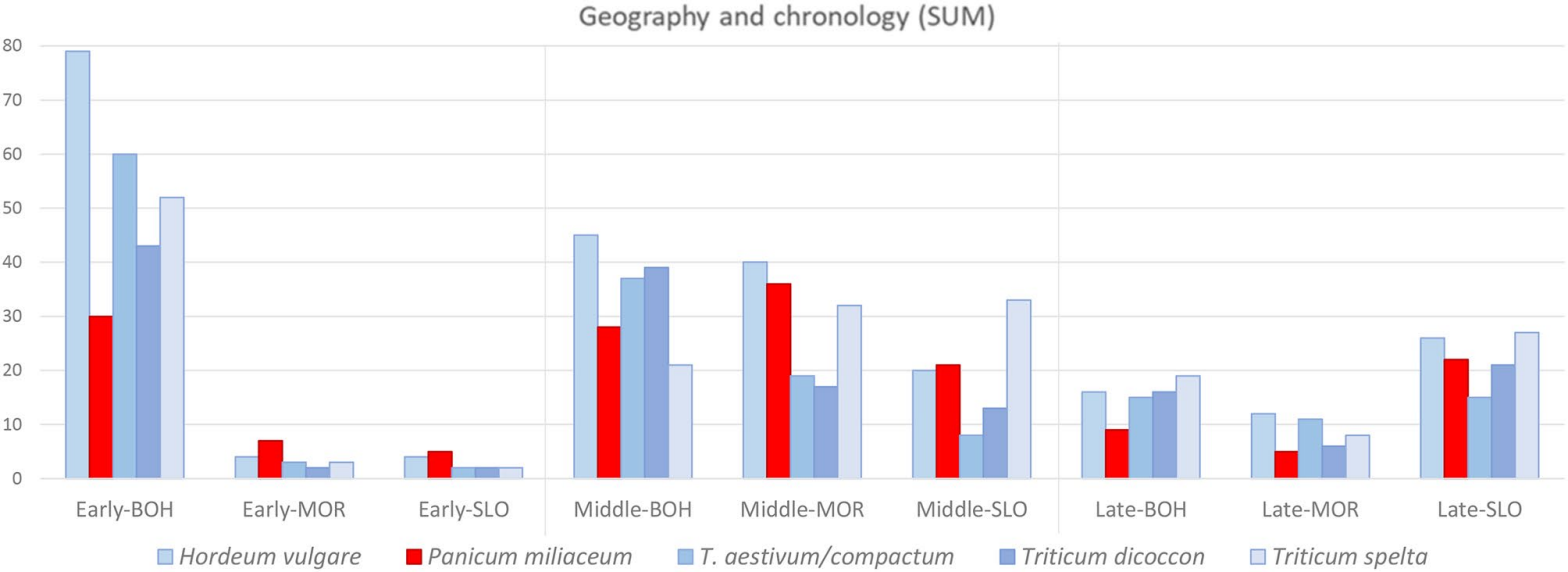
Results of the Representativeness index categorised by chronological period and geographical region and calculated for five principal staple cereals in the analysed territory. Differences in the abundance of archaeobotanical data are particularly evident for the early period between Bohemia compared to Moravia with Slovakia, (graphic by A.D., A.P.).

Results from the Representativeness index (RI) analysis (Table S2) align with findings derived from ubiquity analysis. When comparing aggregated data by individual territories, millet shows the highest RI value in Bohemia, with Moravia and Slovakia both at comparable, much lower levels. When examining millet in relation to other crops, it has a relatively smaller role in Bohemia compared to the two eastern regions, where the RI for millet is similar to barley and spelt. This apparent discrepancy largely arises from the greater abundance of archaeobotanical data from Bohemia (Fig. 3). Thus, in this particular context, the RI primarily reflects data robustness. Aggregated data categorised by chronology indicate that the highest RI values for millet occur during the middle period. This is chiefly because the middle period dataset is statistically the most robust, with relatively balanced contributions from each of the three studied regions.

A more detailed chronological and geographical comparison, evaluating the position of millet among the five principal staple cereals (Fig. 3), reveals a comparatively diminished role for millet in the early period crop spectrum in Bohemia. In contrast, millet appears relatively more prominent in Moravia and Slovakia during the same phase, although the small sample sizes limit the reliability of this observation. For the middle period, millet emerges as the second most important cereal after barley in Moravia. In Bohemia, it ranks behind barley and the wheat species (except spelt), whereas in Slovakia millet and barley both follow spelt in importance. During the late period, Slovakia provides the most abundant data, indicating millet’s importance to be broadly comparable with other staple cereals. Conversely, in Bohemia and Moravia, the available evidence suggests a notably diminished role for millet, placing it behind all other staple crops, although this conclusion remains tentative due to limited data.

### Isotope analysis

**Fig. 4:**
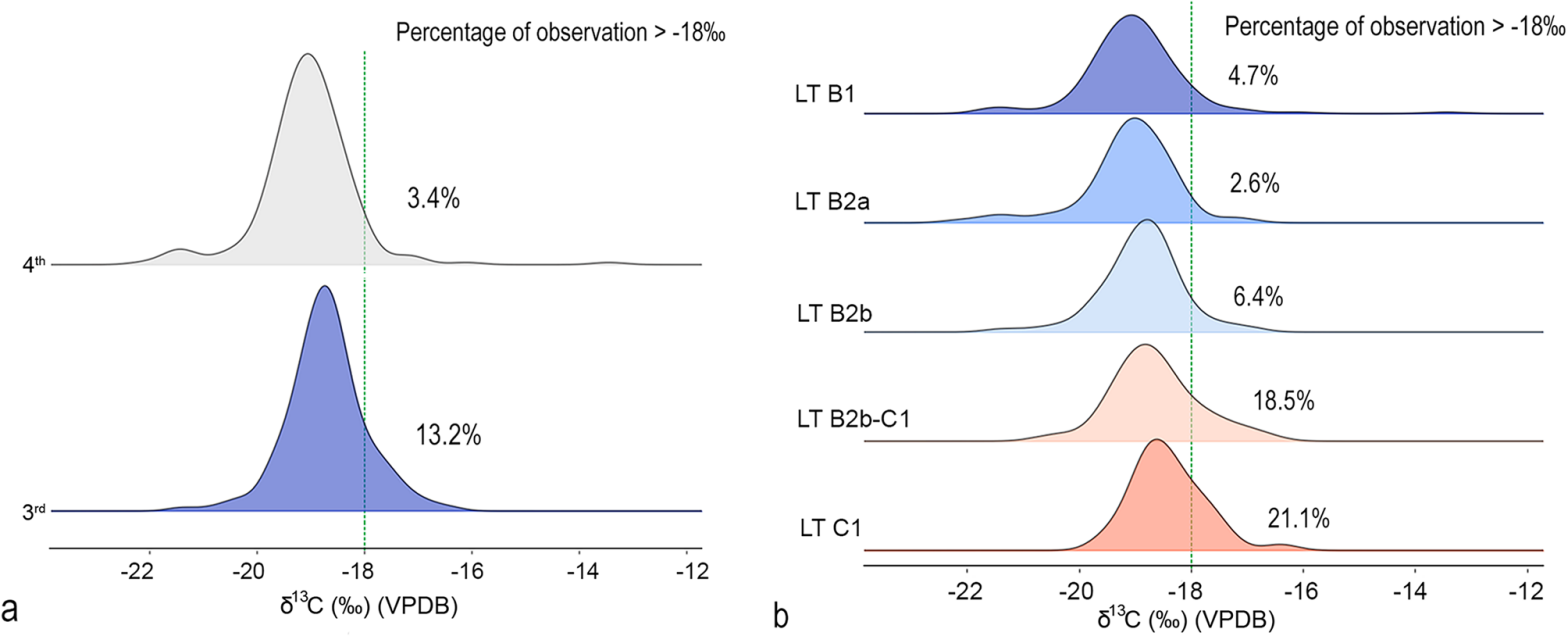
Chronological differences in δ^13^C values between the 4th and the third century BCE for the human dataset represented by KDE plots: per century (**a**) per relative chronological phase (**b**). (graphic by S.K., A.D.).

**Fig. 5:**
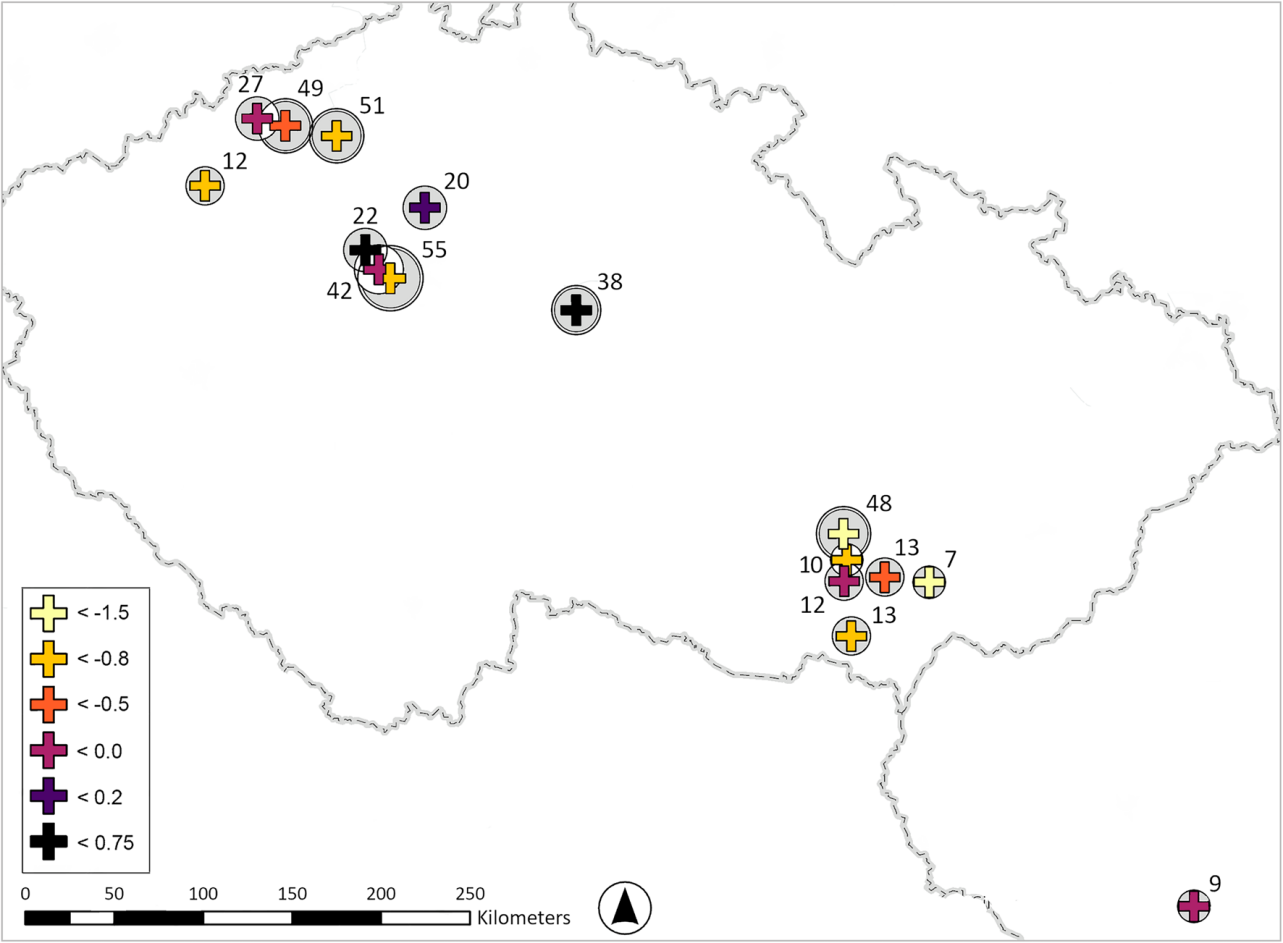
Differences in the shift towards higher mean δ^13^C values between the earliest and latest chronological phases (LT B1 vs. LT C1) for individual cemeteries. The colour legend indicates median Δ^13^C_early-late_ values (positive values mean positive shift, while negative values mean the negative shift), while circles and labels denote site sizes and the number of data per site. Double circles mark sites with *n* > 30 graves (graphic by A.D.).

For the entire human dataset (Fig. 4, 5; Table S9, descriptive statistics in Table S11, for a detailed report on sample categorisation and data analysis, see Supplementary material “SI_analysis”), the difference in average δ^13^C values between the beginning and end of the investigated period (400–180 BCE) is Δ^13^C = 0.61‰. This difference becomes more pronounced when comparing the first and third quartiles (Δ^13^C q_1_ = 0.622‰, Δ^13^C q_3_ = 0.652‰). These observations are statistically significant (Mann–Whitney U test, *p* = 0.000001).

The dataset structure reveals that the early period (400 – 300 BCE) is distinctively variable, including numerous outliers extending to lower δ^13^C values especially in Moravia (Fig. S1, S1.2), where the lowest outliers observed are < -21‰. On the contrary, a group of higher δ^15^N values and a few distinctively positive carbon outliers (> -16‰) are observed in Bohemia (Fig. S1, S1.1). Bohemian sites exhibit greater number of outliers (usually from two to six per site), while Moravian sites have wider ranges of values. This heterogeneity indicates diverse dietary practices and possibly also increased territorial mobility typical for this period^68^. The δ^13^C values become increasingly homogeneous (with non-outlier range about 2.5‰, from − 19.5 to − 17.2‰; Fig. S13, S14, Tables S13, S14) towards the later (´middle´—300 – 180 BCE) period (Mann–Whitney U test, p =  < 0.00005), suggesting perhaps a more structured approach to plant consumption. Later period outliers are almost exclusively in higher δ^13^C direction (with values > -17‰). Especially in the territory of Moravia, the gradual positive shift is more pronounced than in Bohemia (no data are available for Slovakia), (Fig. S14, S15, Tables S15, S16). A statistically significant difference is observed between the latest (LT C1) phase and all the older phases (Kruskal–Wallis test: LT B1–C1, p = 0.000001; LT B2a–C1, p = 0.00017; LT B2b–C1, p = 0.007, Table S14), though the change is visually observable throughout the third century BCE (Fig. S13C). However, this trend is not universally observed across regions (Figs. 5, S1, S14, S15, Tables S16, S17) and varies significantly between individual sites (Fig. S1.1–S1.3).

Geographically, the largest chronological shift towards higher δ^13^C values is particularly evident in the three largest cemeteries of Bohemia and Moravia (Fig. 5, S1.1.2, S1.1.5, S1.2.4). Results from other sites show considerable variability, although a general trend towards greater shifts is apparent in Moravia (Fig. 5). There, the patterns of change are more consistent compared to Bohemia, where some sites even exhibit negative shifts. On average, the positive shift in δ^13^C values at sites where a change is recorded ranges between 0.5‰ and 1.9‰. However, even at sites showing the strongest positive shifts, maximum values do not systematically exceed − 18‰ (VPDB), the threshold generally indicating more significant dietary reliance on millet. The concentration of positive δ^13^C shifts at large cemeteries may equally be influenced by sampling bias, as smaller sites provide fewer data points, however, it is important to note that even among large cemeteries (*n* > 30), δ13C values remain variable, with only three sites exhibiting a clear positive shift of around 1‰ (Figs. 5, S1.1.2, S1.1.5, S1.2.4).

Statistical analysis of demographic age and biological sex groups (Fig. S16–S19, Table S18–S22) indicated that these factors generally had no significant influence on δ^13^C values. The sole exception was a statistically significant difference between biological males and females in the third century BCE (Mann–Whitney U test, p = 0.0076), with females exhibiting slightly higher δ^13^C values, suggesting greater consumption of C₄ plant protein (mean = –18.5‰ for females and –18.9‰ for males). However, this pattern was not consistent across relative chronological phases, and the high proportion of individuals with undetermined sex introduces potential bias into the analysis.

The subsequent analysis focused on potential dietary differences between social groups, as defined by the categorisation of grave good assemblages (SI_analysis). The results (Fig. S20–S23, Table S23–S25) indicate that the chronological shift in δ^13^C values is particularly pronounced in Group 2 (the “common group”). A statistically significant increase in δ^13^C values was observed for this group (Mann–Whitney U test, *p* = 0.004), with values regularly exceeding –17‰ from the LT B2b–C1 phase onwards (mid-third century BCE). Although Group 2 retains the widest overall range of δ^13^C values, the upward shift suggests increased consumption of C₄ plants during this period. In contrast, Groups 1A and 1B display more conservative dietary patterns, with δ^13^C values consistently remaining below the –18‰ threshold, indicating a continued reliance on C₃ plant protein. While a slight increase in δ^13^C values is visible in graphical outputs (Fig. S23), the differences are not statistically significant (Mann–Whitney U test: Group 1A, *p* = 0.102; Group 1B, *p* = 0.1). Social group differences become statistically significant in the LT C1 phase for Group 2 (Kruskal–Wallis test, *p* = 0.01), although the most visually apparent divergence begins in the preceding LT B2b–C1 phase (Fig. S22, S23, Table S24). It should be noted, however, that a substantial number of undated samples in Group 2—due to the absence of datable grave goods—may introduce bias into the analysis.

Finally, available animal isotopic dataset (Table S10) revealed that animal δ^13^C values typically peaked below -19‰, often reaching − 21‰, with the lowest recorded value at -22.2‰. This pattern indicates that animals were predominantly fed a C₃-based diet. Considering the trophic shift, differences of around 2‰ between the mean human and animal δ^13^C values across individual sites (Δ^13^C_humans-animals_) suggest that millet was predominantly part of the human diet and not regularly used as animal fodder (Fig. S2A, B). The variability in animal isotopic values observed across individual sites suggests differences in livestock foddering practices, such as the proportion of forest versus open pastures or varied winter fodder types, reflecting considerable site-to-site variation. No clear geographical trend emerges at the regional scale, though the available data for Moravia remain limited.

In summary, carbon isotopic values from Iron Age cemeteries demonstrate considerable inter-site variability. Although there is an overall trend towards higher δ^13^C values in the later phases (LT B2b—LT C1, before mid-third century BCE), especially in the territory of Moravia, in terms of individual sites, a significant shift occurs only at part of the largest cemeteries. The most pronounced positive δ^13^C shift occurs predominantly among the common group (Group 2), reflecting higher dietary variability within this social cohort. For both rich grave goods groups, δ^13^C values predominantly remain below the –18‰ (VPDB) threshold, along with still a substantial portion of Group 2, indicating that, despite the visibly increased role of millet, C₃ plants continued to form the dietary staple for the studied populations.

## Discussion

### Socio-economic and cultural implications of shifting millet reliance

In this study, the positive carbon isotopic shift, observed for the third century BCE, not accompanied by evidence of statistically significant supra-regional increase in millet cultivation, suggests intensified millet consumption without a corresponding systematic agricultural investment in the crop. This scenario is plausible under several conditions discussed below, highlighting the necessity for critical analysis when integrating multi-proxy data. The irregularity of the isotopic dietary shift observed among the studied sites also necessitates a more nuanced interpretative approach. Recent studies have often linked positive shifts in δ^13^C values to climatic fluctuations, particularly periods of drought^37,38,57,67,69^. While several studies support this interpretation^38,57^, climate models for the late Iron Age period instead indicate cooling conditions and increased precipitation^70^. Furthermore, most Iron Age settlements were established on humus-rich chernozem soils within the traditional agricultural zones of Central Europe^71^, which are more challenging to cultivate using traditional tillage methods during drought conditions. This evidence, considered alongside the broader societal transformations of the third century BCE, suggests that rather than coping with drought, Iron Age communities adopted multiple agricultural strategies aimed at enhancing overall productivity, diversity, and resilience. More importantly, the results of the archaeobotanical analysis show that this fluctuation occurred without millet ever overtaking the other staple crops in cultivation, suggesting that it was a stable part of the staple crops spectrum that only provided resilience if needed. Therefore, even if millet functioned exclusively as a buffer crop during periods of unfavourable climatic conditions, its use in the Iron Age does not appear to have been a uniform or widespread adaptive strategy.

An important factor for the interpretation of the temporary surge of millet consumption is therefore its selective and variable character. It underscores the capacity of Iron Age communities to flexibly adjust agricultural strategies according to emerging socio-economic needs on a more complex level; a dynamic relevant for understanding adaptive governance in contemporary production systems^72^. Where the positive isotopic shift was recorded within the analysed area, it was typically associated with larger communities (Fig. 5); only in Moravia is the shift detectable more broadly across the region. The larger cemeteries are predominantly situated within the most agriculturally productive zones, where the industrial agglomerations were established during the course of the third century BCE. The concentration of population within these settlements, coupled with growing economic specialisation, and proximity to long-distance communication networks, required corresponding developments in agriculture aimed at increasing yields and ensuring a consistent surplus. Evidence for intensification in the animal husbandry with focus on working animals in the case of agglomerations in the Middle Danube region^12^, implies an expansion of cultivation areas within their hinterlands to enhance crop production. Under these conditions, millet would have been particularly attractive due to its agronomic characteristics, including flexibility, short growing season, high yield potential, and suitability for long-term storage^17,49^. Its fast maturation and summer planting cycle meant it could be slotted into agricultural schedules with relative ease and could serve as a catch-crop or a second harvest or be cultivated on soils and in microclimates less suitable for barley and wheat. These attributes offered farming communities considerable flexibility, enabling them to adapt effectively to fluctuating agricultural needs. Furthermore, millet cultivation may represent a deliberate strategy of agricultural intensification—broadening crop diversity and reinforcing the role of spring-sown cereals alongside traditional staples—to support growing populations in nucleated settlements without requiring a complete shift in dietary practices. This interpretation is reinforced by archaeobotanical evidence from several middle period agglomerations in Western-Central Europe, where millet ranks second in importance to barley^10,13,73^, a position also similar to ubiquity ratios, MNI, and Representativeness index of millet from contemporaneous Moravian sites (Figs. S7, S11). Conversely, settlements outside these zones may have continued cultivating a staple crop spectrum adapted to local conditions, with variable emphasis on individual crops and local livestock farming. These data collectively point towards substantial flexibility and autonomy among farming communities, each employing subsistence strategies shaped by specific local contexts and preferences. Only during the third century BCE, within emerging agglomerations where economic practices likely became more structured, does the positive δ^13^C shift appear more systematic—particularly in Moravia, which by this time had become integrated into the wider network of economically progressive Middle Danube zone. In contrast, the adoption of millet-enhanced dietary patterns appears generally more irregular in Bohemia, as visible also in the millet ubiquity ratios and MNI in the archaeobotanical data (Fig. S7, S11). This variability may reflect the peripheral position of Bohemian communities relative to agricultural innovations emerging in the Middle Danube region, with only a few larger sites being integrated into this emerging network.

Another factor shaping variability in dietary patterns is the role of social structures, cultural preferences, and possibly mobility from regions where millet consumption was more common. Previous studies document intensive interaction between the study area and southeastern regions during the third century BCE, involving shared trade networks, technological exchange, and cultural influences^5^. Demonstrating the presence of direct migrants from these areas, however, requires additional evidence. Strontium isotope data from the Czech Republic, where available alongside dietary isotopes^68,74,75^, show no consistent link between higher δ^13^C values with ^87^Sr/^86^Sr ratios typical of the Carpathian Basin^76^. The overall similarity of strontium signatures across loess-dominated lowland zones in Central Europe^77^ complicates precise identification of migrants and limits the ability to directly attribute dietary shifts to mobility. This is supported by the absence of consistent correlations between δ^13^C outliers and strontium isotope values. Instead, the variability appears to reflect local dynamics. Most δ^13^C outliers belong to biological females or individuals from the “common population” (Group 2), whereas groups 1A and 1B are rarely represented (Fig. S21, S22). This suggests that dietary change was primarily an endogenous process affecting the broader population. The two other groups, while following the general trend, retained more conservative and homogeneous dietary profiles, showing only modest increases in δ^13^C values and continued preference for C₃ plants (Fig. S23). This may reflect socio-cultural preferences among some, perhaps better-provisioned, households, which allowed them to sustain distinct dietary standards regardless of broader shifts in the available food spectrum.

It is noteworthy that during the late La Tène phase (180 BCE – 0 BCE/CE), millet’s role within the staple crop spectrum markedly declined (Fig. S8, S12). This suggests that millet cultivation was no longer a key component of the systematic agricultural strategies adopted by the emerging socio-economic centres in Central Europe from the second century BCE onward. One possible explanation is that oppida—often established at higher elevations—required different cultivation strategies adapted to local environmental conditions. However, archaeobotanical evidence also shows a decline in millet at lowland rural settlements, indicating that its earlier prominence may have been specific to lowland agglomerations. Following the abandonment of these sites, rural communities appear to have reverted to locally adapted agricultural practices, while the oppida developed distinct cultivation regimes, often placing greater emphasis on livestock-based economies and more resilient crop types^78–83^.

All these findings was not a universally adopted agricultural innovation, but rather a preferential dietary choice shaped by geography, economic strategy, cultural tradition, and social organisation. This interpretation highlights how prehistoric communities either adapted or conservatively maintained their foodways in response to environmental and socio-cultural dynamics.

### Integrating archaeobotanical and isotopic evidence

Combining archaeobotanical data with stable isotope analysis enables a more comprehensive reconstruction of ancient dietary practices than either approach can achieve alone. Isotopic analysis can detect broad dietary trends (such as the relative importance of C₃ versus C₄ plants or trophic levels) while archaeobotanical evidence provides direct, physical records of specific cultivated plants and their local management. Nevertheless, integrating these two approaches is not always straightforward. Macro-remains typically reflect short-term or site-specific food availability due to their context-dependent preservation. In contrast, stable isotope data from bone collagen represent dietary intake averaged over extended periods of adolescence due to the variable turnover rate of collagen especially in various types of bones^84–86^. Aligning these datasets chronologically and contextually thus presents challenges, particularly when isotopic and archaeobotanical data originate from different sites. Where direct comparisons are not feasible, interpretations should minimise potential errors by carefully selecting assemblages from burial and settlement sites that are as closely matched in time and space as possible. Additionally, developing a detailed understanding of regional archaeological and archaeobotanical patterns is crucial. Therefore, significant attention must be paid to precise dating, classification and contextualisation of the data, enabling meaningful comparisons between individual sites at both local and regional scales. As highlighted in the results, dietary patterns identified from isotopic evidence varied considerably among individual sites; thus, comparisons with archaeobotanical data at a local scale were essential to determine whether millet proportions within local crop spectra diverged from broader, supra-regional trends. However, even when working with datasets compatible chronologically and spatially, the presence of particular plant species within a crop assemblage does not have to necessarily reflect their dietary importance. Beyond simple quantification of macro remains, it is therefore essential to incorporate additional measures such as ‘ubiquity ‘ and the ‘Representativeness index ‘. Only after establishing the ‘economic importance’ of a plant taxon through rigorous archaeobotanical methods, the potential discrepancies between archaeobotanical and isotopic evidence can be approached from the perspective of the true dietary significance of that plant within the studied human populations.

## Conclusion

This study demonstrates that the increased reliance on millet during the third century BCE in La Tène Central Europe was a context-dependent, socially stratified, and regionally variable process. Isotopic and archaeobotanical evidence reveal that this dietary shift was not uniform across time or space: the most significant increase occurred in lowland agglomerations during the mid-La Tène period, while millet remained marginal in upland oppida and declined again after 180 BCE. It was also revealed that while millet consumption intensified among certain segments of the population, particularly individuals with simpler grave goods, those buried with warrior or rich female attire maintained more conservative diet with limited millet intake. These developments likely reflect the emergence of nucleated, industrial-style agglomerations, accompanied by population growth and a resulting need to adapt agricultural production towards more efficient and sustainable practices. The observed patterns, both systemic and locally variable, highlight the value of multi-proxy approaches for understanding human adaptability. Only by informed integrating archaeobotanical and isotopic evidence can the nuanced role of millet in Iron Age diets be fully understood.

More broadly, the findings suggest that past dietary practices were shaped not only by environmental conditions, but also by cultural preferences and technological change, resulting in locally adapted strategies. This interplay between resilience, adaptability, and social identity offers meaningful comparative insights for modern societies striving toward more flexible and sustainable food systems.

## Methods

Both the isotopic and archaeobotanical analyses followed a chronological and geographical structure, dividing the study area into three main regions (Bohemia, Moravia, Slovakia) and three chronological periods. To mitigate potential biases in regional relative dating, used by archaeologists, when defining phases, absolute date ranges were used for the chronology. This approach allowed for a more accurate consideration of the site’s beginning and end, particularly for settlements where the precise duration of occupation is often difficult to determine. Isotopic data are available only for certain parts of the early and middle phases (400–180 BCE) and are accordingly grouped into two phases corresponding to the fourth century and the third century BCE, respectively. In addition to the division into the main chronological periods, the datasets and statistical outputs also incorporate relative chronological phases as defined in the Central European Iron Age chronology allowing for more detailed chronological overview^87^. The complete chronological framework used for this study is as follows:

- Botanical data:
- Early phase (500–330 BCE)
- Middle phase (330–180 BCE)
- Late phase (180 BCE–0 BCE/CE)
- Isotopic data:
- Early phase (400–300/290): LT B1, LT B2a
- Middle phase (300/290–200/180 BCE): LT B2b, LTB2b-C1, LT C1

The phases in the archaeobotanical dataset are extended beyond chronological delimitation of periods, because the settlements are dated in longer chronological intervals.

### Botanical analysis

The archaeobotanical data were collected from both published and unpublished sources and originated from 99 archaeological sites providing 143 analytical units including sites or site-related chronological-phase assemblages (Table S3). The archaeobotanical dataset comprises all available data on cultivated and wild plant seeds accessible to the authors from Slovakia (Table S4). For Moravia and Bohemia, both published and unpublished archaeobotanical data from the Czech Republic, compiled in the Czech Archaeobotanical Database, were collected. Plant macro-remain data were assembled for all cultivated plants (cereals, pulses, and oil/fibre crops), potentially edible wild fruits and nuts, as well as the total number of weed seeds. For each taxon, the total number of identified specimens (NISP) of grains, seeds, and chaff, along with the number of samples in which the taxon was recorded, were documented for each site and chronological phase (Tables S5, S6). Grain and chaff data were combined to calculate the MNI (minimum number of individuals) of potential grains per site. Ratios of grain to chaff components were applied as follows: einkorn (1:2), emmer, spelt, and timopheevii (2:2), naked wheat and barley (3:1), rye (2:1), and oats (1:1) (Table S7). Additionally, presence/absence and ´ubiquity´ ratios (the number of samples in which a taxon occurred divided by the total number of samples from a site) were used to mitigate biases from differing methodologies, researchers, or geographical contexts. To address data variability, the data were standardised by calculating taxon percentages relative to the total (MNI values) of identified cereal finds, excluding indeterminate grain fragments. For sites with fewer than 5 samples, modelled ubiquity data (calculated in two ANOVA runs) were employed to address small sample size constraints. For millet, values ranging from zero to the phase average were replaced with the lower quartile, while values above the average were replaced with the upper quartile of the phase value (Table S8). Sites with fewer than 50/30 finds (depending on percentage calculations) or those with broad chronological ranges were excluded when evaluating ubiquity ratios.

The statistical evaluation aimed to identify chronological and spatial patterns of millet in the study area. Archaeobotanical data were analysed at the site or site´s chronological phase level. A Detrended Correspondence Analysis (DCA) was conducted to investigate variations in cereal crop composition across sites from different chronological periods or regions. This analysis utilised two standardised matrices of cereal crops: (a) percentages and (b) ubiquities (ratios) per site. DCA was performed using Canoco for Windows^88^. Differences in central values and distributions among groups were tested using the non-parametric Kruskal–Wallis test and the parametric one-way ANOVA. Additionally, the Representativeness index (RI) was calculated, which assesses both the abundance and dominance of individual taxa in relation to the total macrofossil count and the number of analysed samples from each site. The RI was calculated using the approach of Stika and Heiss^89^. By combining frequency, ubiquity, and RI measures, this approach provides the most reliable approximation of the economic importance of millet within the given time period and study area.

The cartographical distribution of the data (ArcGIS Pro, ESRI Inc.) includes information on both the percentage and ubiquity of millet at the analysed sites (Fig. S5–S12). The graphic outputs utilise pie charts to represent the proportion of millet in crop spectrum per site. The size of the pie charts is normalised according to the total cereal counts at each site for percentage charts and number of samples for ubiquity charts.

### Isotope analysis

Data on the isotopic composition of collagen carbon and nitrogen from La Tène cemeteries in the region, along with archaeological contextualisation regarding dating and grave goods, were compiled into a single dataset containing 429 samples from 16 burial sites, with available δ^13^C and δ^15^N collagen values and archaeological contextualisation. The data were compiled from the available publications and also include 79 new isotopic data from the Moravian sites of Blučina, Nechvalín, Lovčičky, and Brno Maloměřice that were recently analysed (Table S9, S12, SI_protocol). Where available, the isotopic data from animals were included in the dataset; this information was available for 9 sites and included 87 samples (Table S10). Chronological variations were examined between the fourth and third centuries BCE (the latter period being of particular interest for identifying socio-economic transformations) as well as across relative chronological phases.

The statistical analysis of the isotopic dataset (SI_analysis) focused on identifying chronological, social, and geographical patterns in the study region. Individual sites were analysed separately, examining carbon and nitrogen isotopic compositions across chronological phases and associating them with defined main social categories, grouped as “warrior equipment” (group 1A), “rich female attires” (group 1B), and “general population” (group 2). To account for environmental and dietary variations among sites and regions, isotopic data from animals were also collected where possible and compared with human data.

Statistical differences among group central values and distributions were tested using non-parametric tests (Mann–Whitney U, Kruskal–Wallis) as they are more efficient for analysing subsets of data and non-normal distributions. The resulting *p*-value was subsequently assessed in conjunction with data visualisation. Individual groups were also compared using kernel density estimation (KDE). KDE is a non-parametric method for estimating the probability density function of continuous variables, providing a smooth, distribution-independent representation of the data. Studies have demonstrated that KDE provides a more precise and flexible method for analysing isotope data^90^. This approach allows for capturing the potential trends in millet consumption. The cartographical distribution of the data (ArcGIS Pro, ESRI Inc., https://www.arcgis.com), used both in the manuscript and supplementary files (Fig. S3−S12) includes spatial information on recorded isotopic shifts. If not stated explicitly, for all the background maps the World Topographic Map by © Esri and NASA, NGA, USGS, GUGiK, ŠOP SR, Esri TomTom, Garmin, FAO, METI/NASA, was used.

## Supplementary Information

Supplementary Information 1.

Supplementary Information 2.

Supplementary Information 3.

Supplementary Information 4.

Supplementary Information 5.

## Data Availability

All data reported in this article are provided in the Supplementary Electronic Materials (SEM). Specifically, archaeobotanical date are provided in SEM Table 4, stable isotopic data (δ13C, δ15N) are provided in SEM Tables 9 and 10 (with references), the values and quality indicators of the stable isotope analyses conducted for this paper are provided in SEM Protocol, detailed report on statistical evaluation of stable isotopic data is provided in SEM SI_analysis.
