# Supplementary material for: Multiproxy evidence of millet reliance and selective dietary change during iron age transformation in Central Europe
Source: Sci Rep. 2025 Nov 21;15:41364. doi: 10.1038/s41598-025-25274-z (PMC12638749; doi:10.1038/s41598-025-25274-z)

Figure S1.

Scatterplots of δ^{13}C (‰) (VPDB) and δ^{15}N (‰) (VAIR) values for the stable isotopic datasets in the study area categorised by region and chronology.

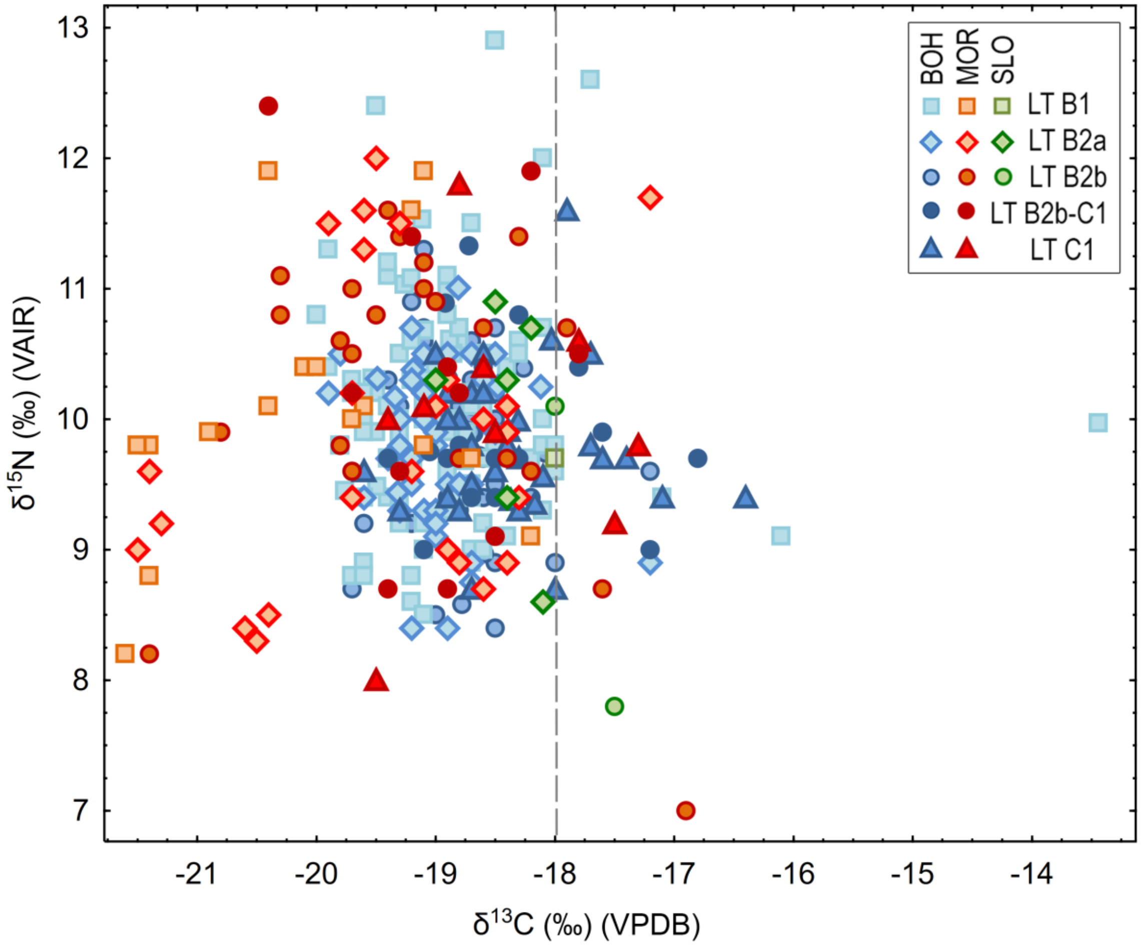

Figures S1.1.-1.3. – individual burial sites

For the location of burial sites, please see the SEM map 2.

Legend:

A) Dating

- LT B1
- LT B2a

} 4th century BC

- LT B2b
- LT B2b-C1
- LT C1

} 3rd century BC

B) Social group

- Group 1A (warrior equipment)
- Group 1B (rich costume attire and/or grave goods)
- Group 2 (common population)
  - common or poor costume attire and/or grave goods
  - no grave goods
- Group 1S (non-standard combination of grave goods)

Dashed line marks -18‰ treshold

Figure S1.1. - Bohemia

(S1.1.1.) JENIŠŮV ÚJEZD

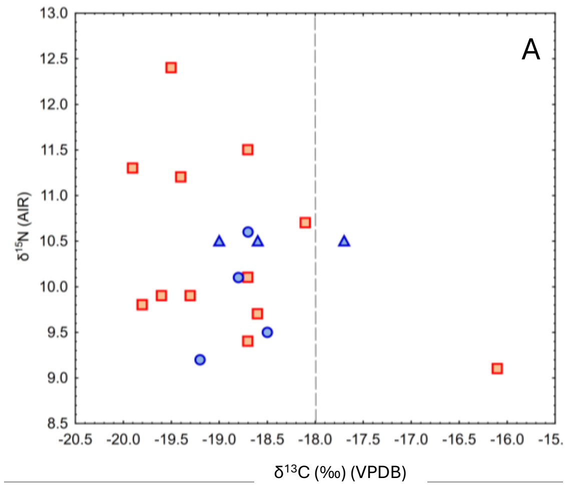

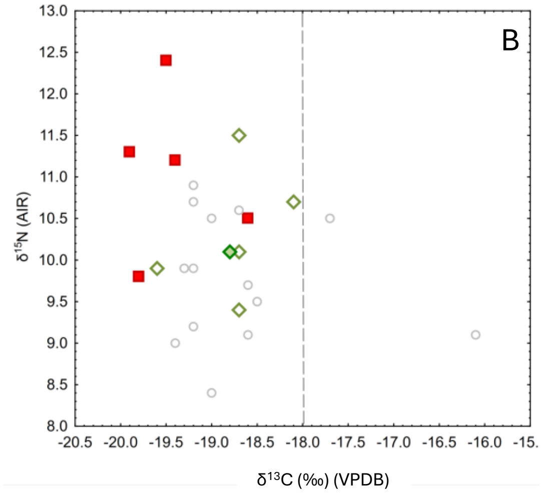

(S1.1.2.) JINONICE

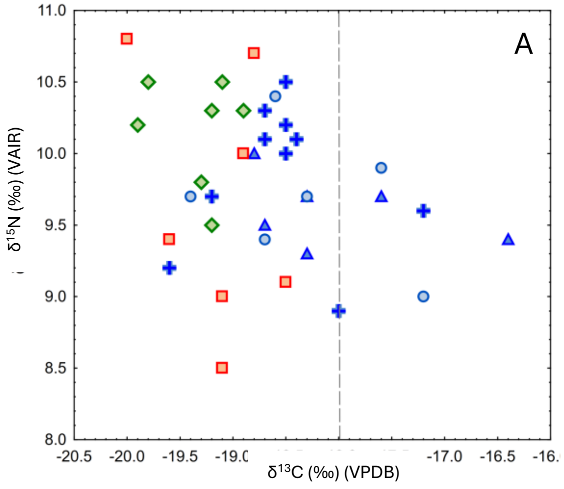

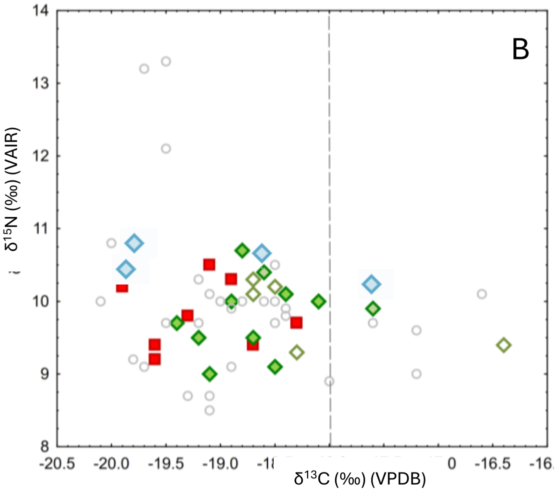

(S1.1.3.) KUTNÁ HORA

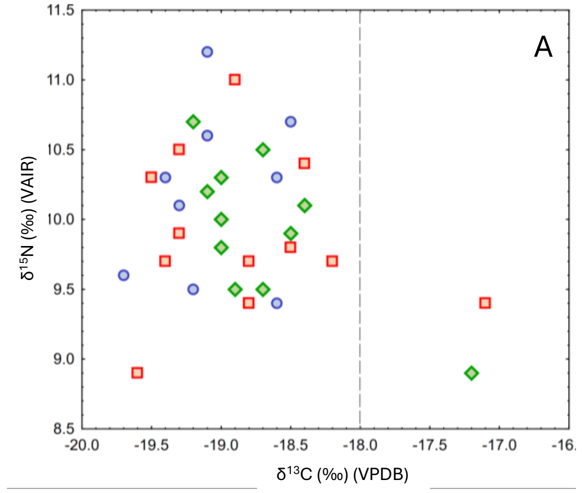

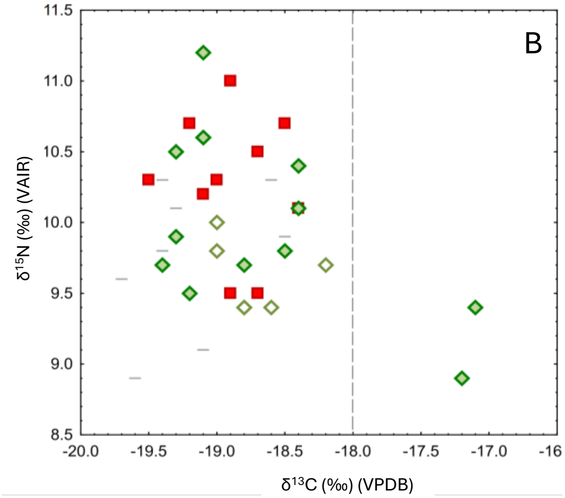

(S1.1.4.) MAKOTŘASY

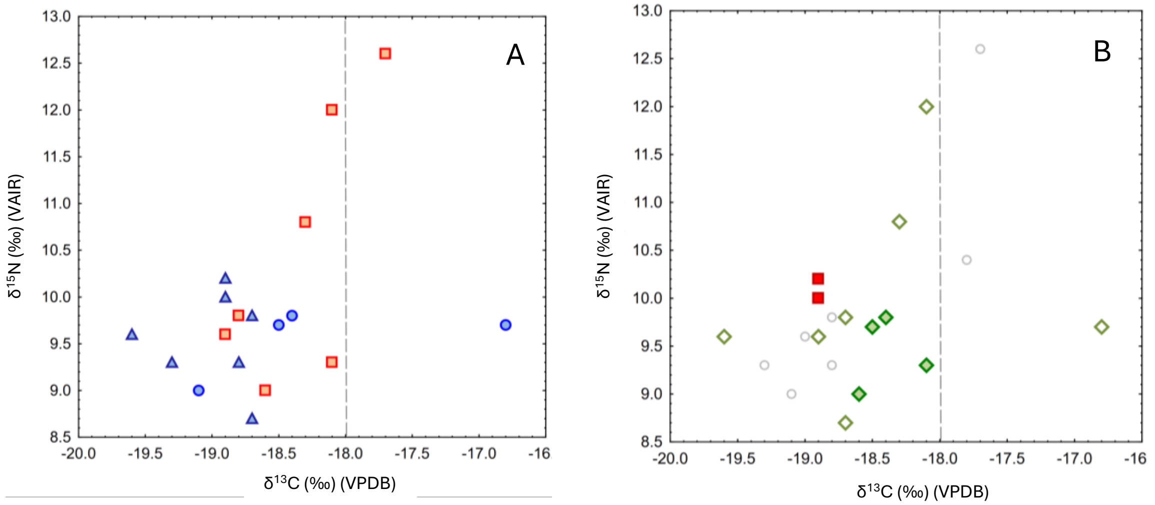

(S1.1.5.) PROSMYKY

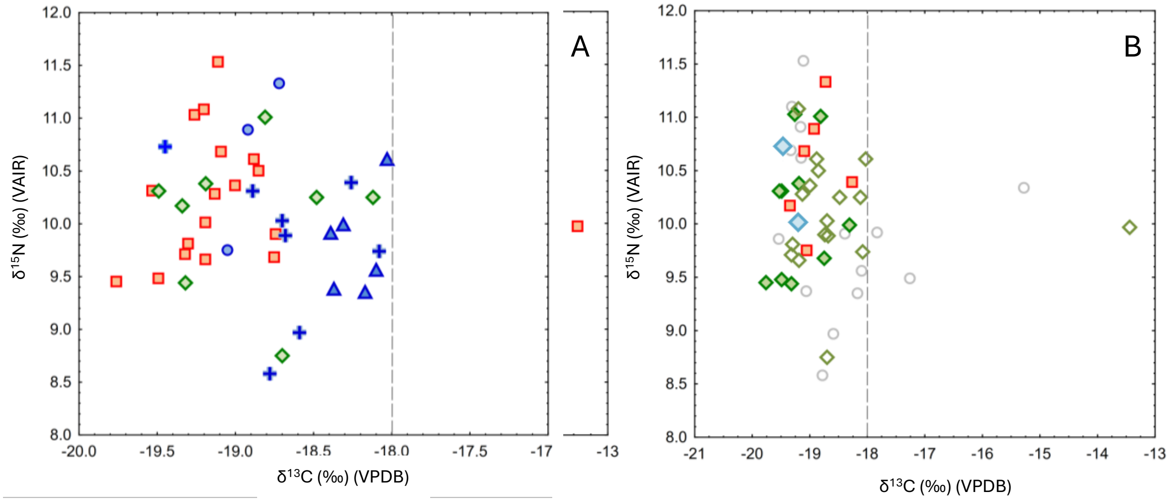

(S1.1.6.) RADOVESICE 1, 2

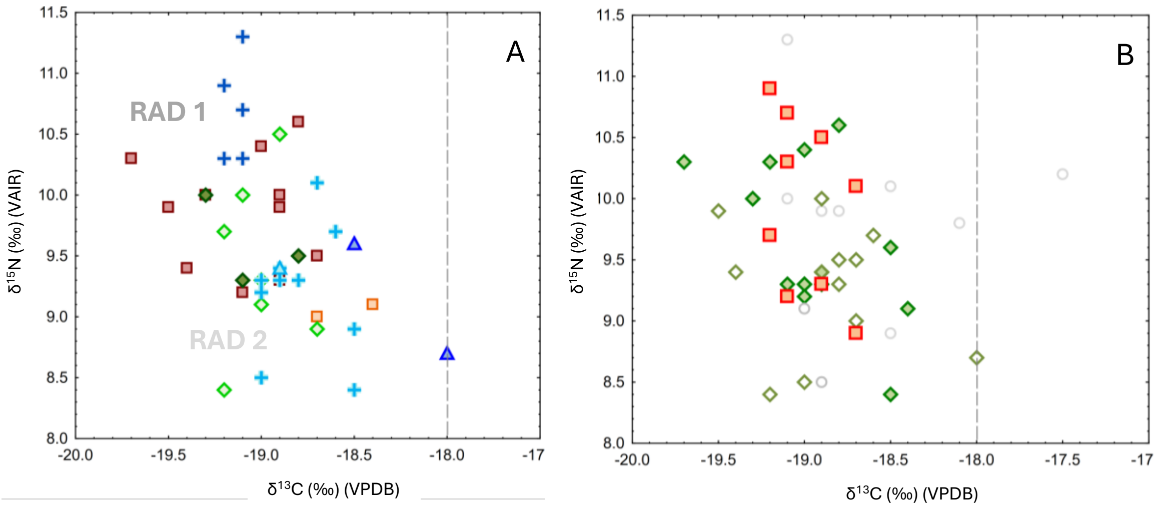

(S1.1.7.) RUZYNĚ

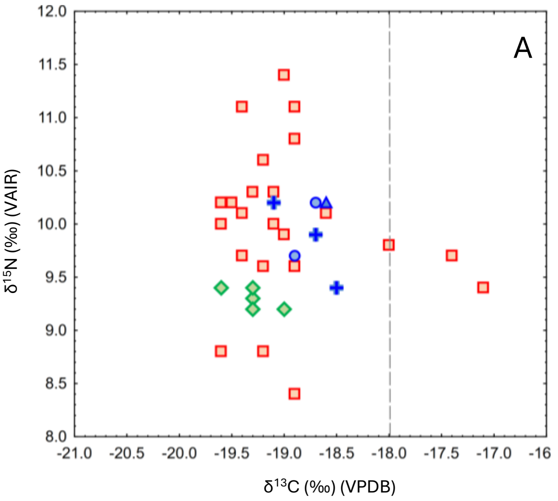

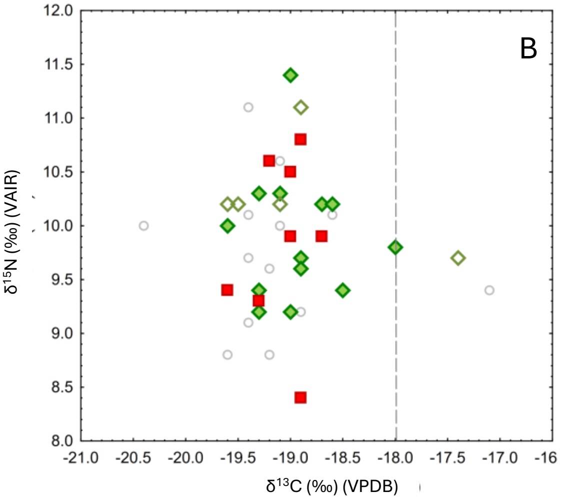

(S1.1.8.) TIŠICE

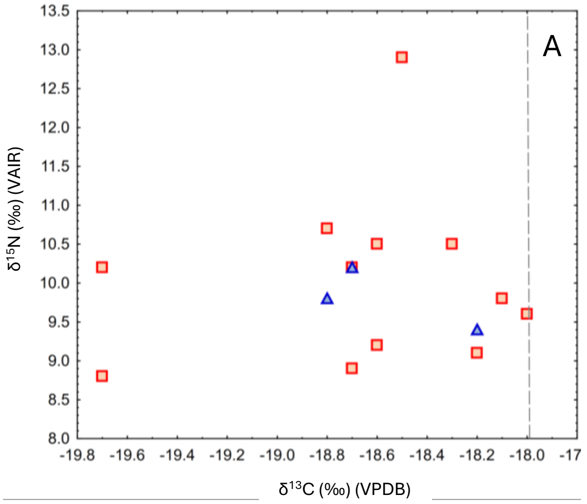

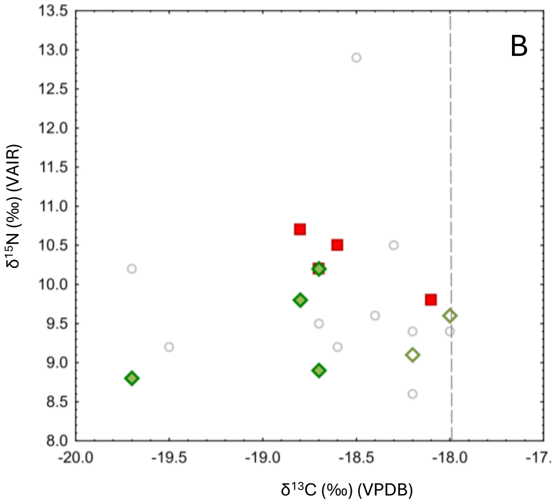

Figure S1.2. - Moravia

(S1.2.1.) BLUČINA

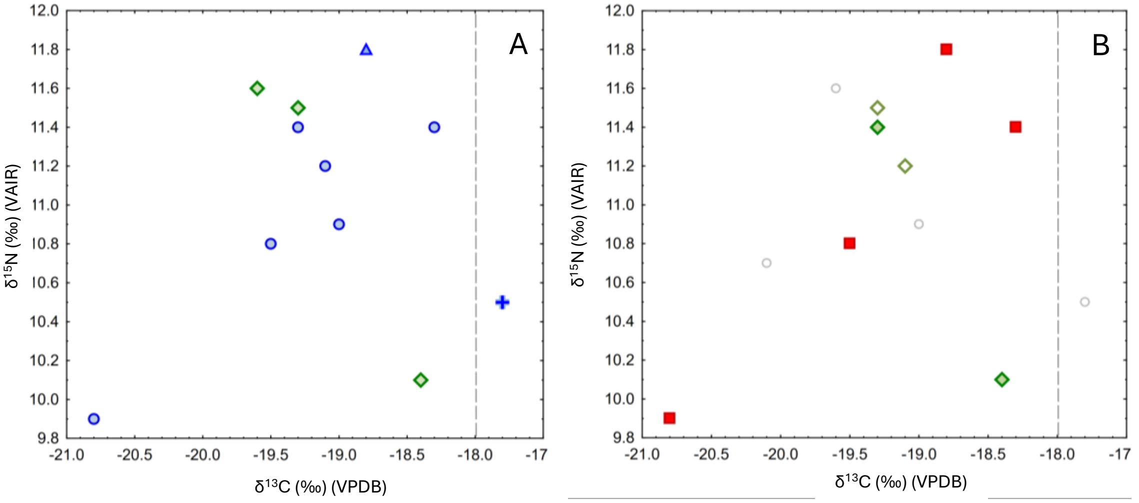

(S1.2.2.) BRNO - CHRLICE

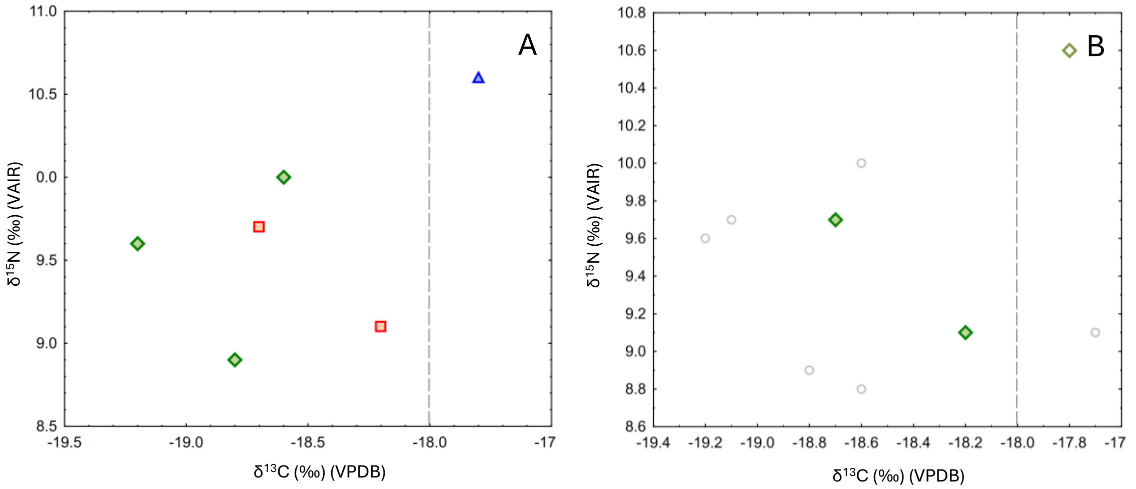

(S1.2.3.) LOVČIČKY

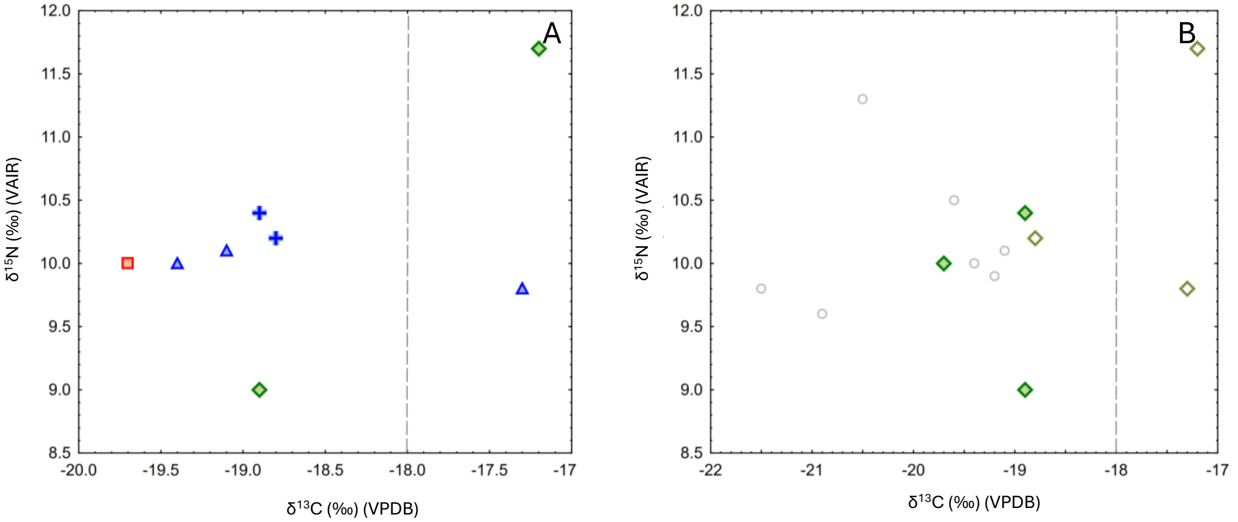

(S1.2.4.) BRNO MALOMĚŘICE

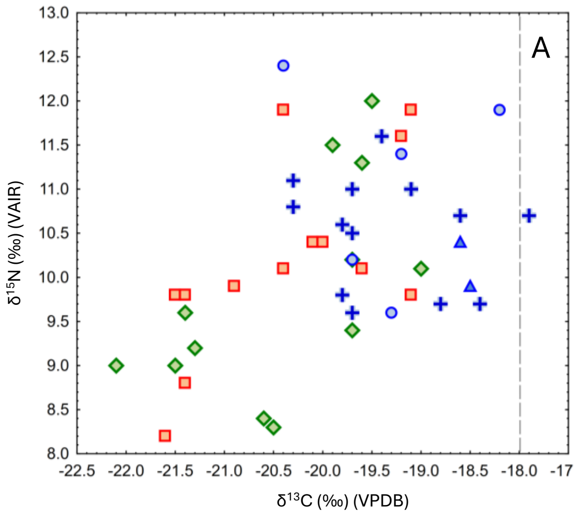

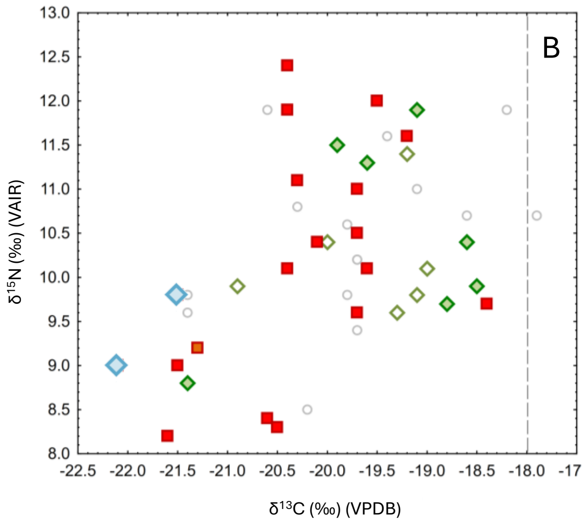

(S1.2.5.) NECHVALÍN

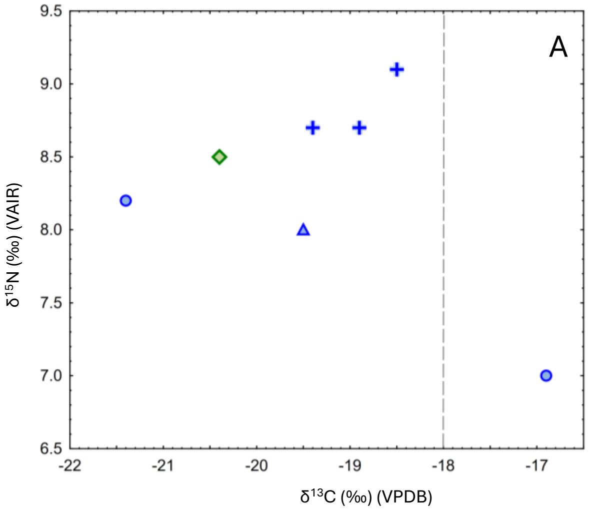

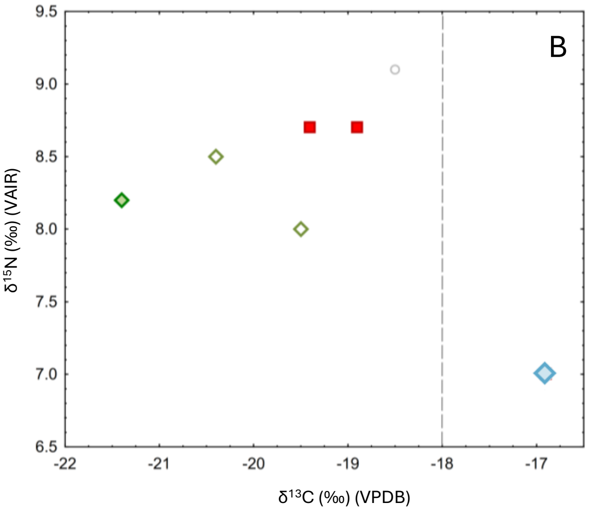

(S1.2.6.) PAVLOV

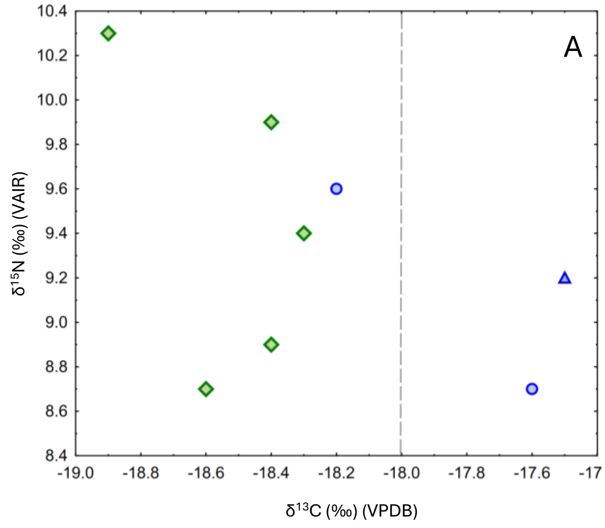

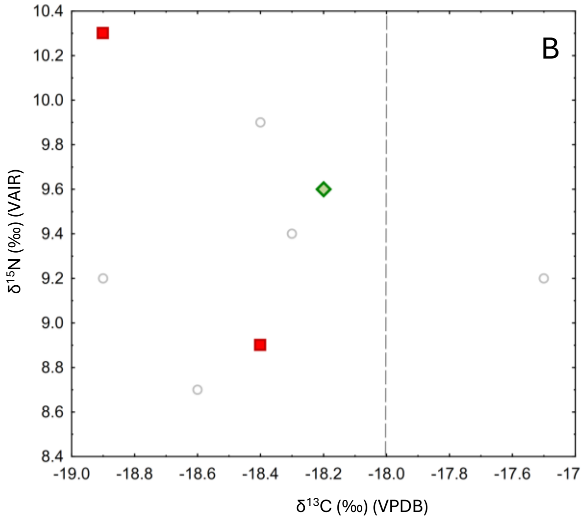

Figure S1.3. - Slovakia

(S1.3.1.) DUBNÍK

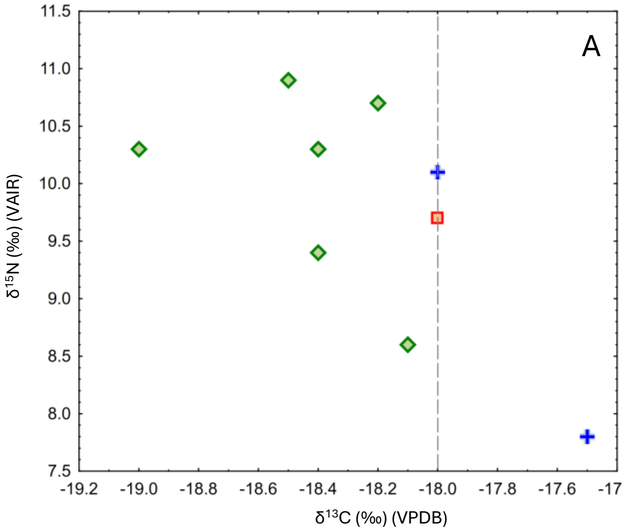

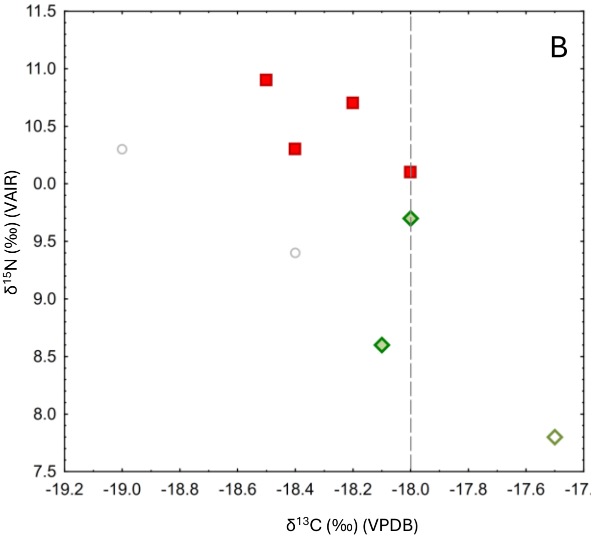

Figure S2.A: δ^{13}C and δ^{15}N values of animal datasets at individual cemeteries.

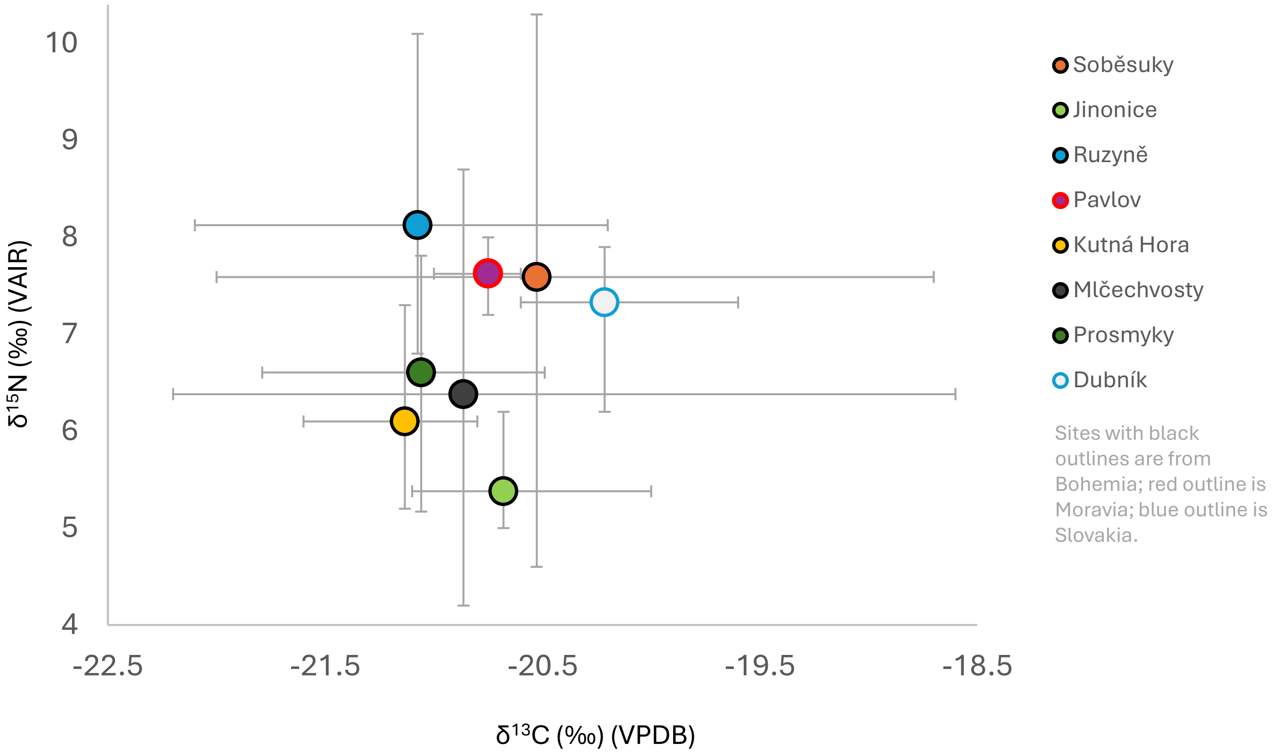

Figure S2.B: Differences in mean carbon isotopic values between humans and animals ($\Delta^{13}C_{humans\text{-}animals}$) at individual sites.

As animal data lack chronological resolution, the comparison is applied to the entire dataset regardless of chronology. Circles and labels indicate site sizes and the number of graves per site.

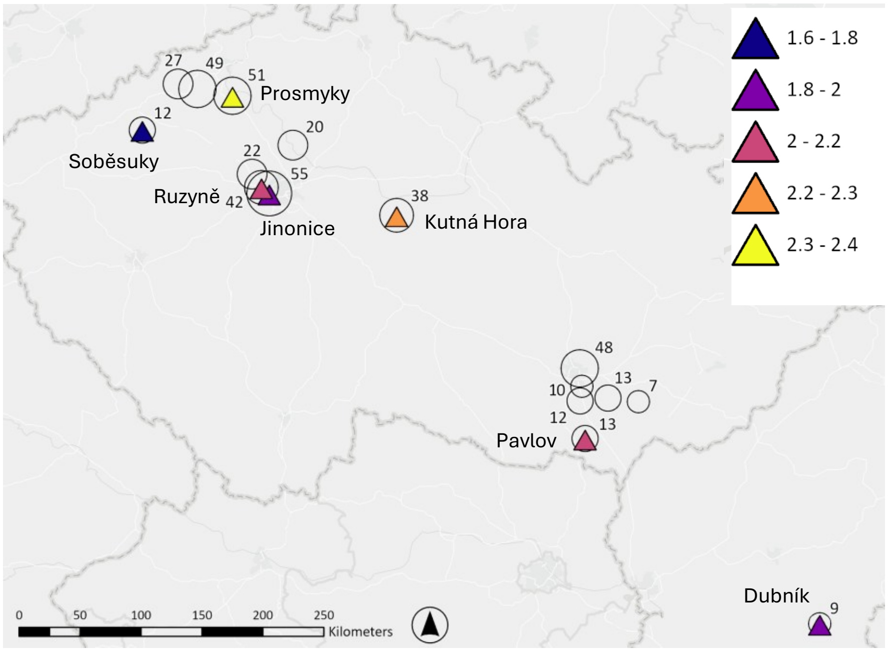

Supplement: Supplementary file 1 — Supplementary Information 1. [file 41598_2025_25274_MOESM1_ESM.pdf]
