# Supplementary material for: Multiproxy evidence of millet reliance and selective dietary change during iron age transformation in Central Europe
Source: Sci Rep. 2025 Nov 21;15:41364. doi: 10.1038/s41598-025-25274-z (PMC12638749; doi:10.1038/s41598-025-25274-z)
Supplement: Supplementary file 2 — Supplementary Information 2. [file 41598_2025_25274_MOESM2_ESM.pdf]

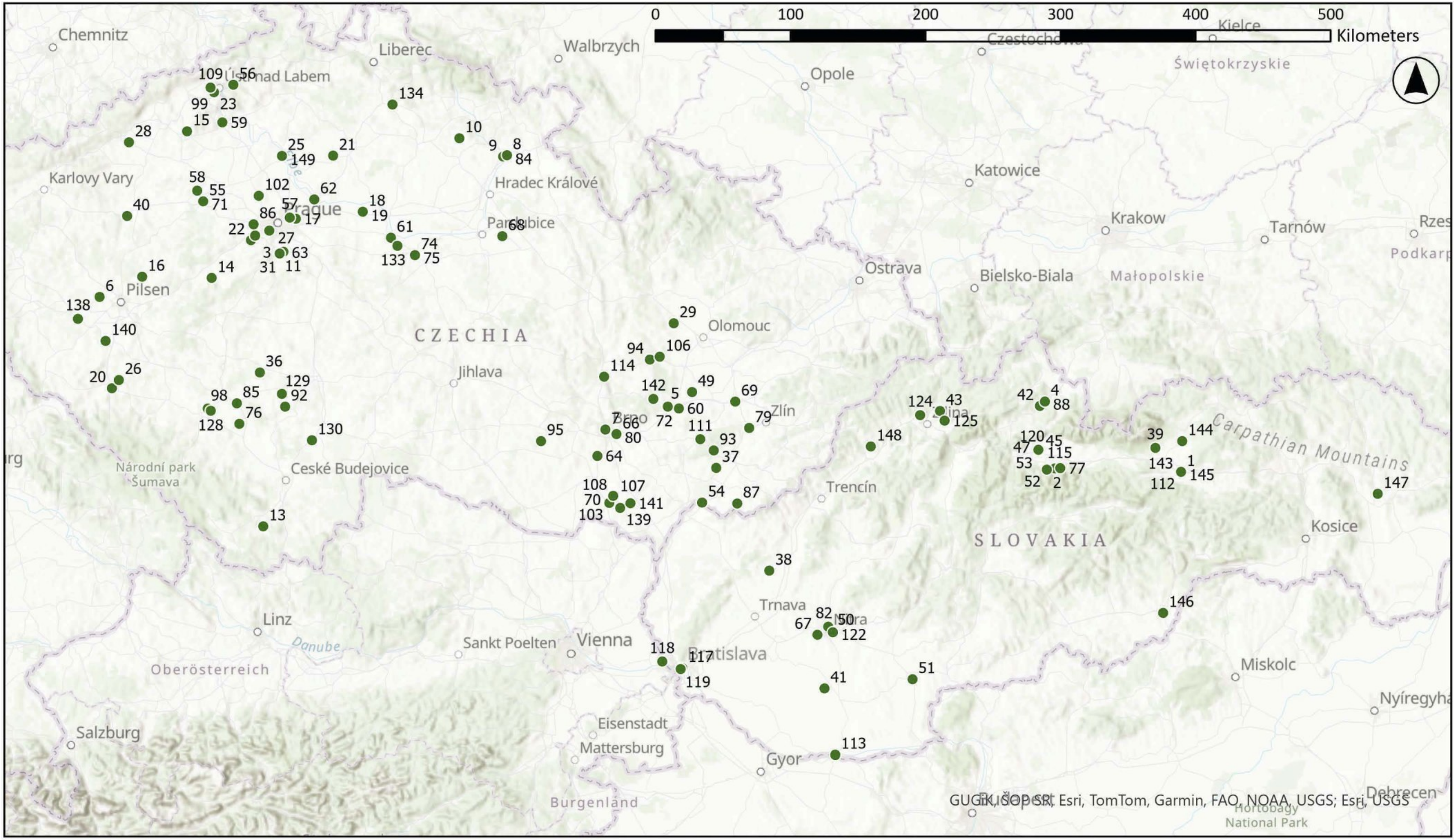

Figure S3. Sites with archaeobotanical data from the study area (Bohemia, Moravia, Slovakia). For the site names, see SEM Table 4.

Image created with software AcrGIS Pro, www.arcgis.com. Background map: World Topographic Map by © Esri and NASA, NGA, USGS, GUGiK, ŠOP SR, Esri TomTom, Garmin, FAO, METI/NASA

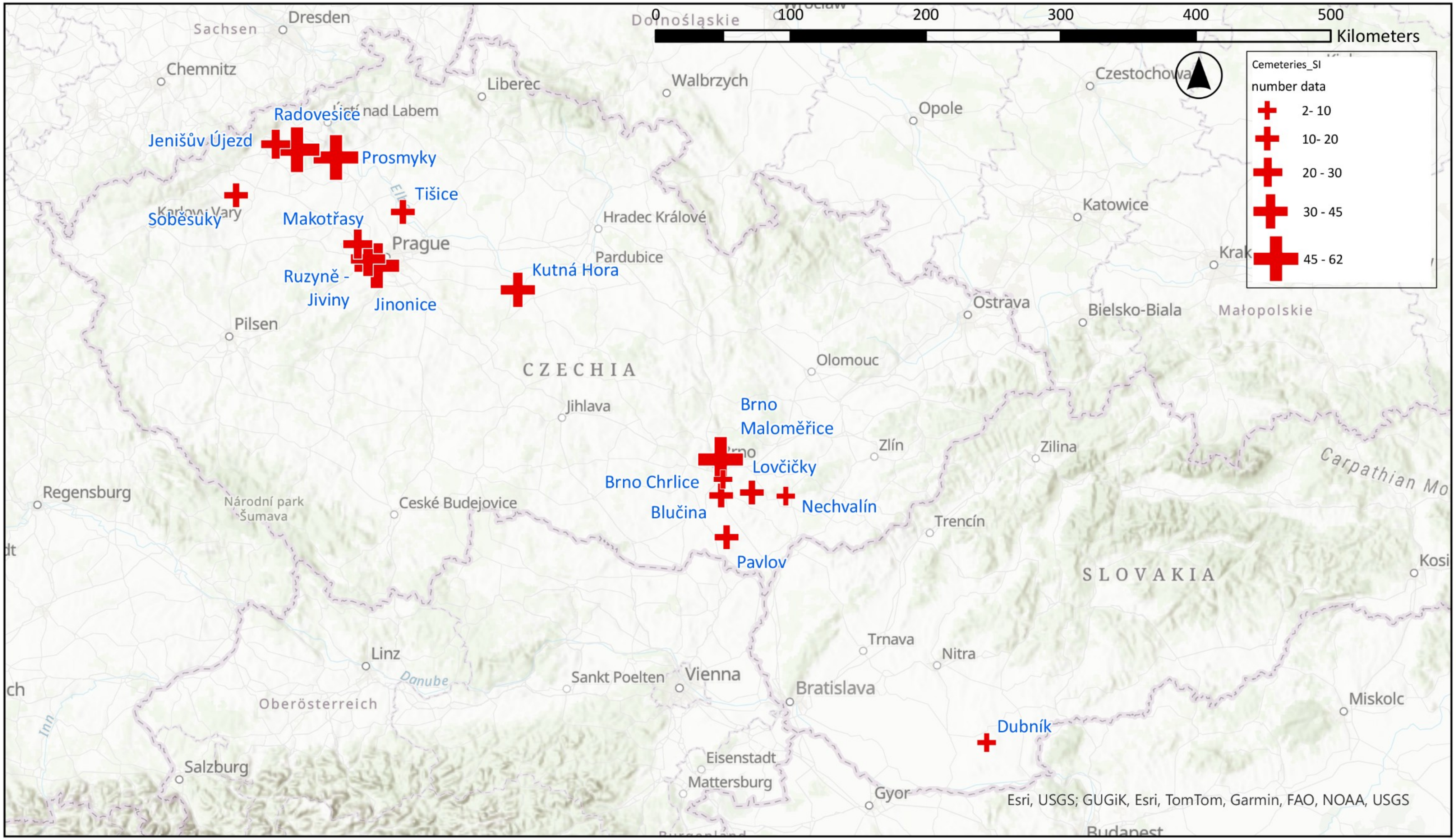

Figure S4. La Tène period cemeteries (400 – 180 BCE) in the study area (Bohemia, Moravia, Slovakia) with available isotopic data. The symbol size is normalised by the number of burials (i.e. the size of the cemetery; „number data“ in the Legend inset). For the isotopic dataset, see SEM Table 9, 10.

Image created with software AcrGIS Pro, www.arcgis.com. Background map: World Topographic Map by © Esri and NASA, NGA, USGS, GUGiK, ŠOP SR, Esri TomTom, Garmin, FAO, METI/NASA

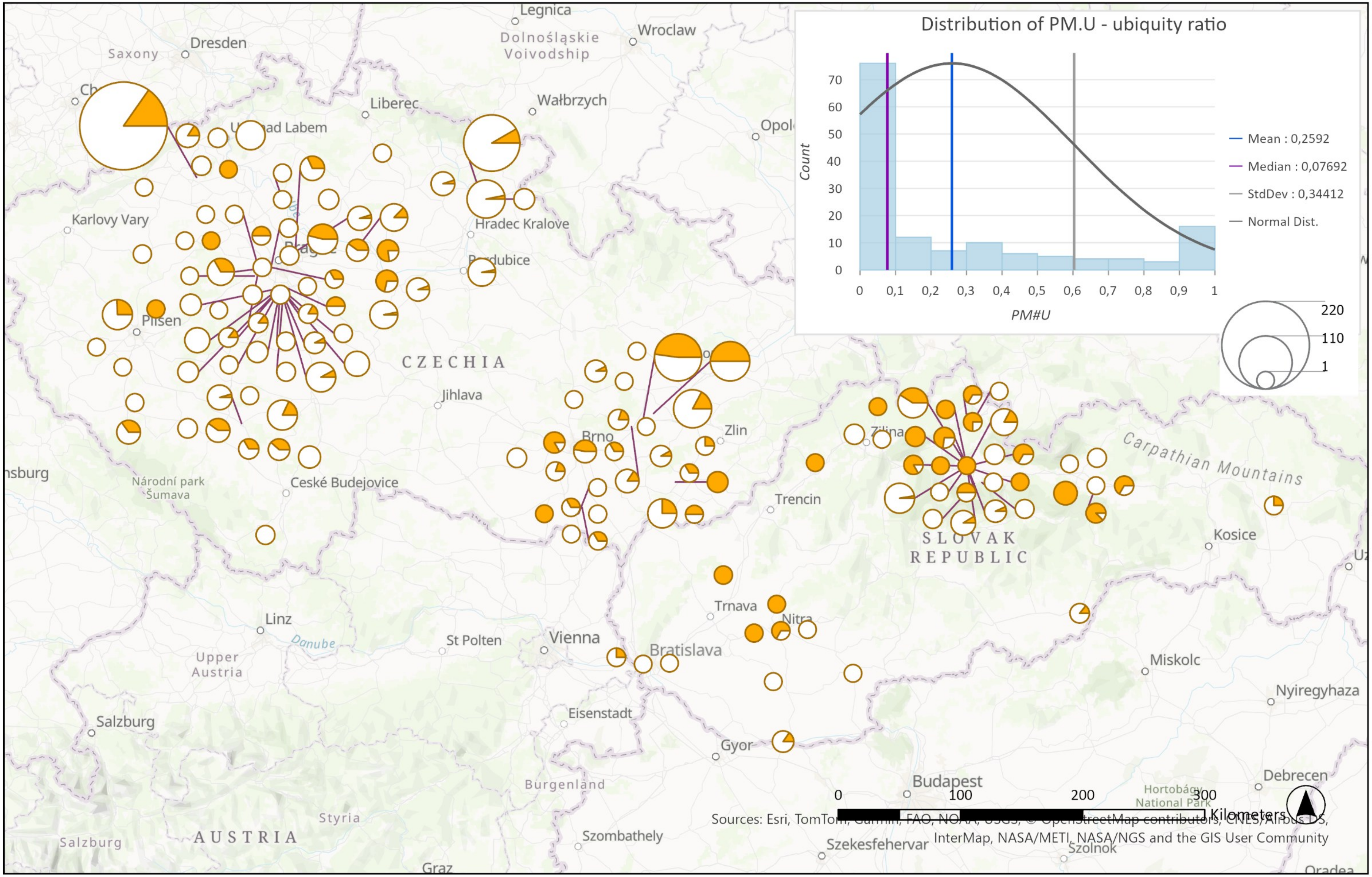

Figure S5. Pie charts for the **ubiquity ratio** of millet at sites in the study area (Bohemia, Moravia, Slovakia) during the **whole period (500 - 0 BCE/ CE).** Chart sizes are normalised by the number of samples per site. Inset - histogram of the ubiquity ratio values with basic statistics.

Image created with software AcrGIS Pro, www.arcgis.com. Background map: World Topographic Map by © Esri and NASA, NGA, USGS, GUGiK, ŠOP SR, Esri TomTom, Garmin, FAO, METI/NASA

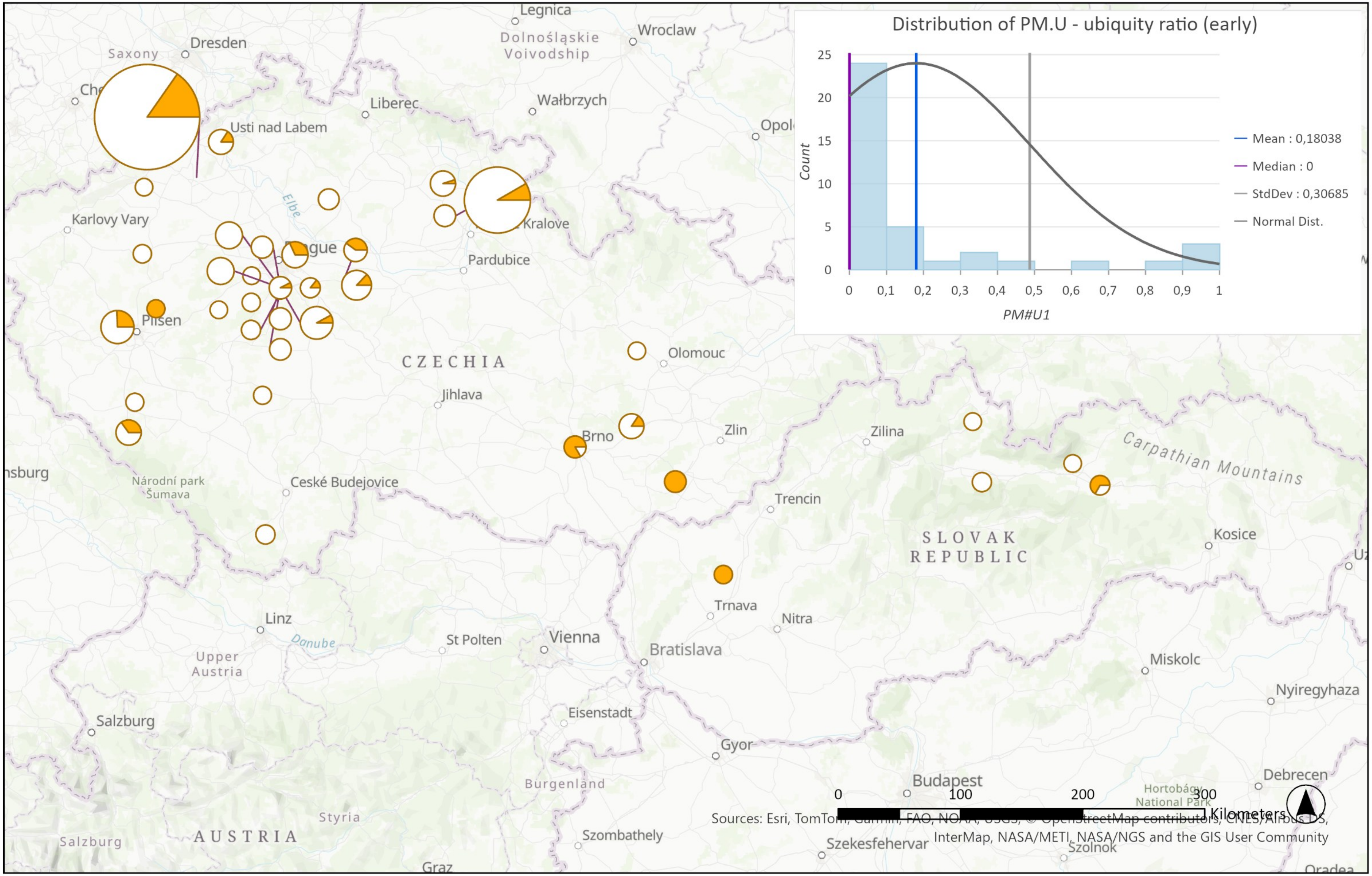

Figure S6. Pie charts for the **ubiquity ratio** of millet at sites in the study area (Bohemia, Moravia, Slovakia) during the **early period (500 - 330 BCE)**. Chart sizes are normalised by the number of samples per site. Inset - histogram of the ubiquity ratio values with basic statistics.

Image created with software AcrGIS Pro, www.arcgis.com. Background map: World Topographic Map by © Esri and NASA, NGA, USGS, GUGiK, ŠOP SR, Esri TomTom, Garmin, FAO, METI/NASA

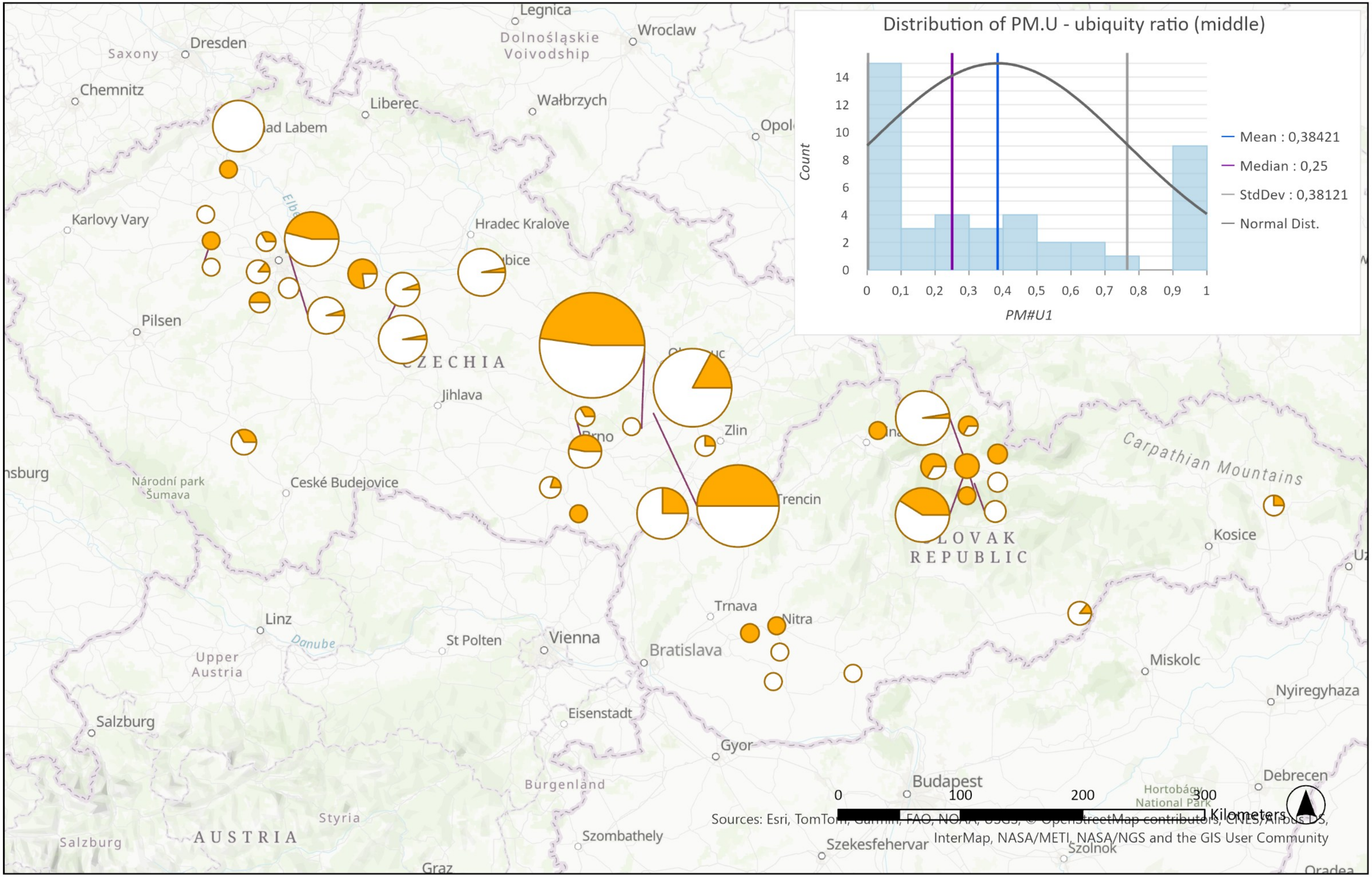

Figure S7. Pie charts for the **ubiquity ratio** of millet at sites in the study area (Bohemia, Moravia, Slovakia) during the **middle period (330 - 180 BCE)**. Chart sizes are normalised by the number of samples per site. Inset - histogram of the ubiquity ratio values with basic statistics.

Image created with software AcrGIS Pro, www.arcgis.com. Background map: World Topographic Map by © Esri and NASA, NGA, USGS, GUGiK, ŠOP SR, Esri TomTom, Garmin, FAO, METI/NASA

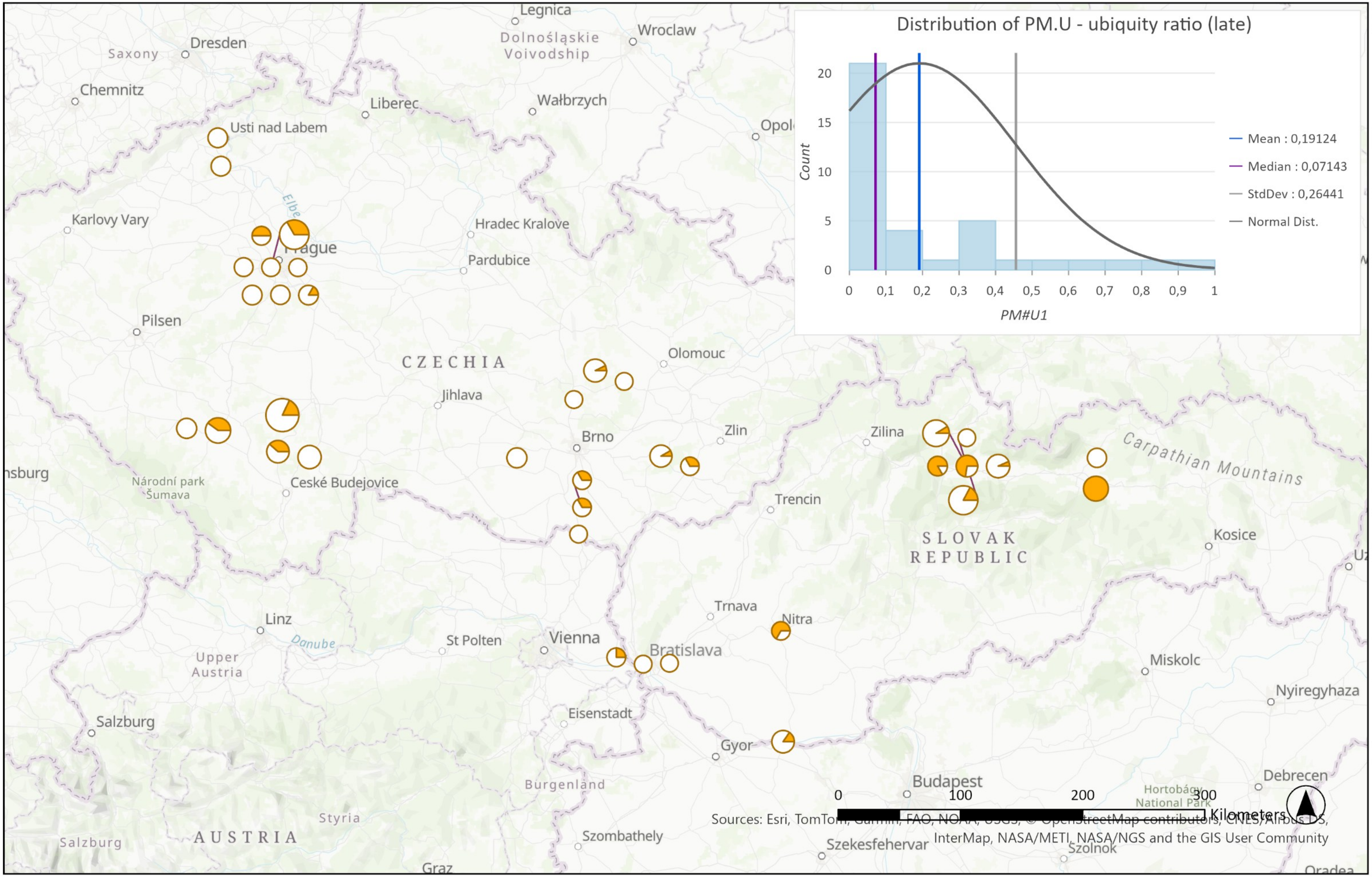

Figure S8. Pie charts for the **ubiquity ratio** of millet at sites in the study area (Bohemia, Moravia, Slovakia) during the **late period (180 - 0 BCE/ CE).** Chart sizes are normalised by the number of samples per site. Inset - histogram of the ubiquity ratio values with basic statistics.

Image created with software AcrGIS Pro, www.arcgis.com. Background map: World Topographic Map by © Esri and NASA, NGA, USGS, GUGiK, ŠOP SR, Esri TomTom, Garmin, FAO, METI/NASA

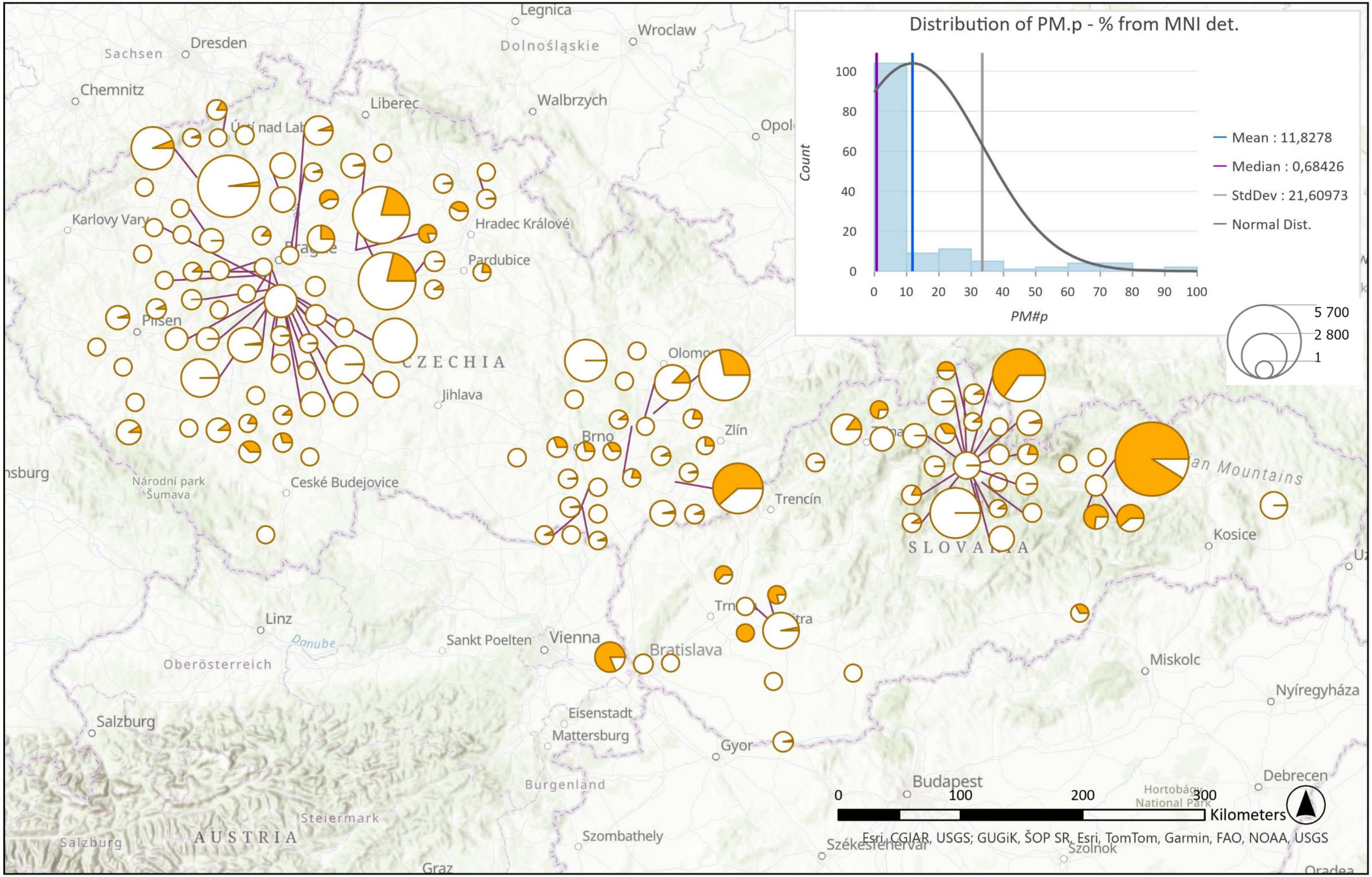

Figure S9. Pie charts for the **% from MNI determined** of millet at sites in the study area (Bohemia, Moravia, Slovakia) during the **whole period (500 - 0 BCE/ CE).** Chart sizes are normalised by the number of cereal species finds (including fragments). Inset - histogram of the % from the MNI determined values with basic statistics.

Image created with software AcrGIS Pro, www.arcgis.com. Background map: World Topographic Map by © Esri and NASA, NGA, USGS, GUGiK, ŠOP SR, Esri TomTom, Garmin, FAO, METI/NASA

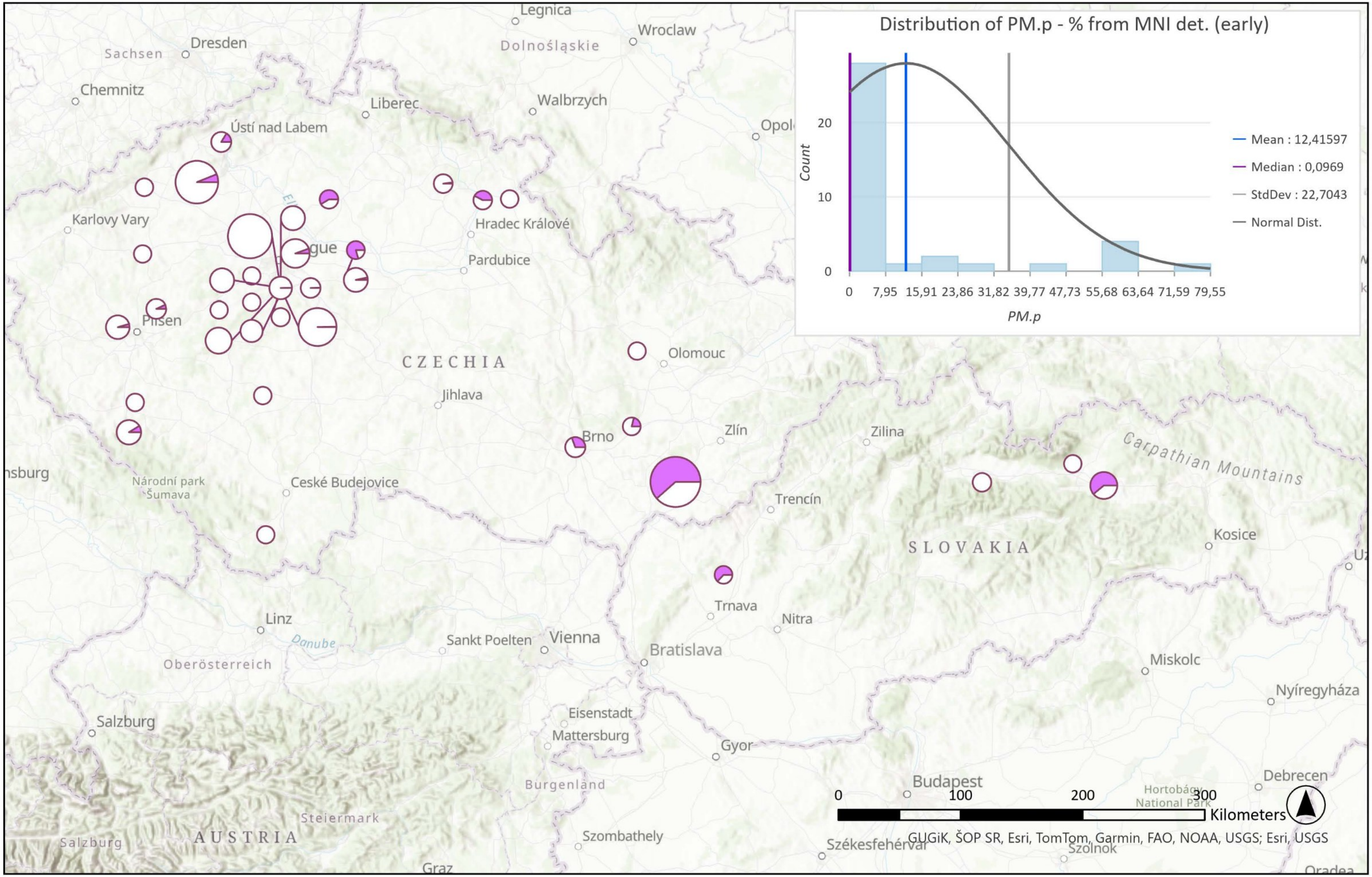

Figure S10. Pie charts for the **% from MNI determined** of millet at sites in the study area (Bohemia, Moravia, Slovakia) during the **early period (500 - 330 BCE).** Chart sizes are normalised by the number of cereal species finds (including fragments). Inset - histogram of the % from the MNI determined values with basic statistics.

Image created with software AcrGIS Pro, www.arcgis.com. Background map: World Topographic Map by © Esri and NASA, NGA, USGS, GUGiK, ŠOP SR, Esri TomTom, Garmin, FAO, METI/NASA

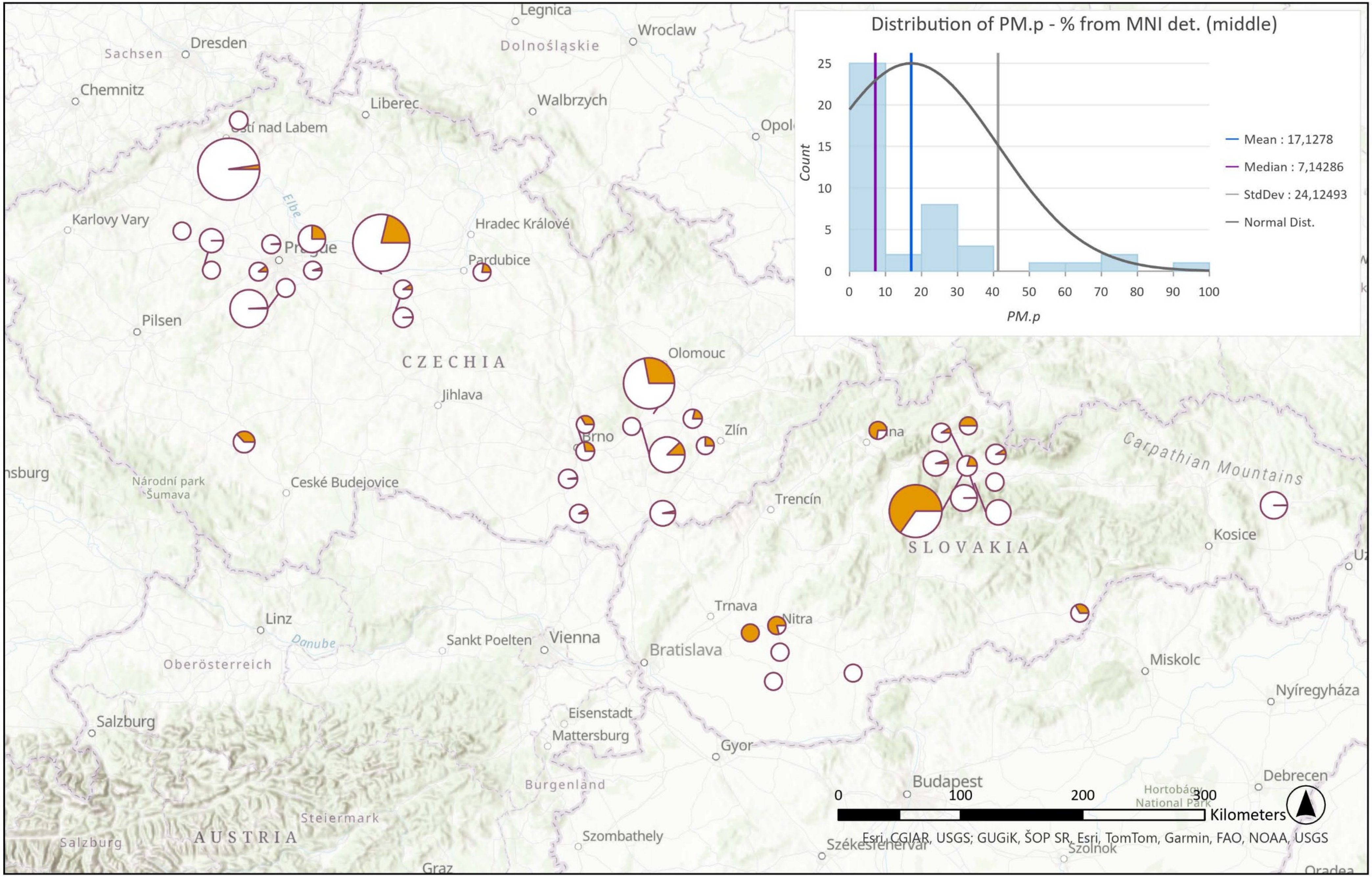

Figure S11. Pie charts for the **% from MNI determined** of millet at sites in the study area (Bohemia, Moravia, Slovakia) during the **middle period (330 - 180 BCE).** Chart sizes are normalised by the number of cereal species finds (including fragments). Inset - histogram of the % from the MNI determined values with basic statistics.

Image created with software AcrGIS Pro, www.arcgis.com. Background map: World Topographic Map by © Esri and NASA, NGA, USGS, GUGiK, ŠOP SR, Esri TomTom, Garmin, FAO, METI/NASA

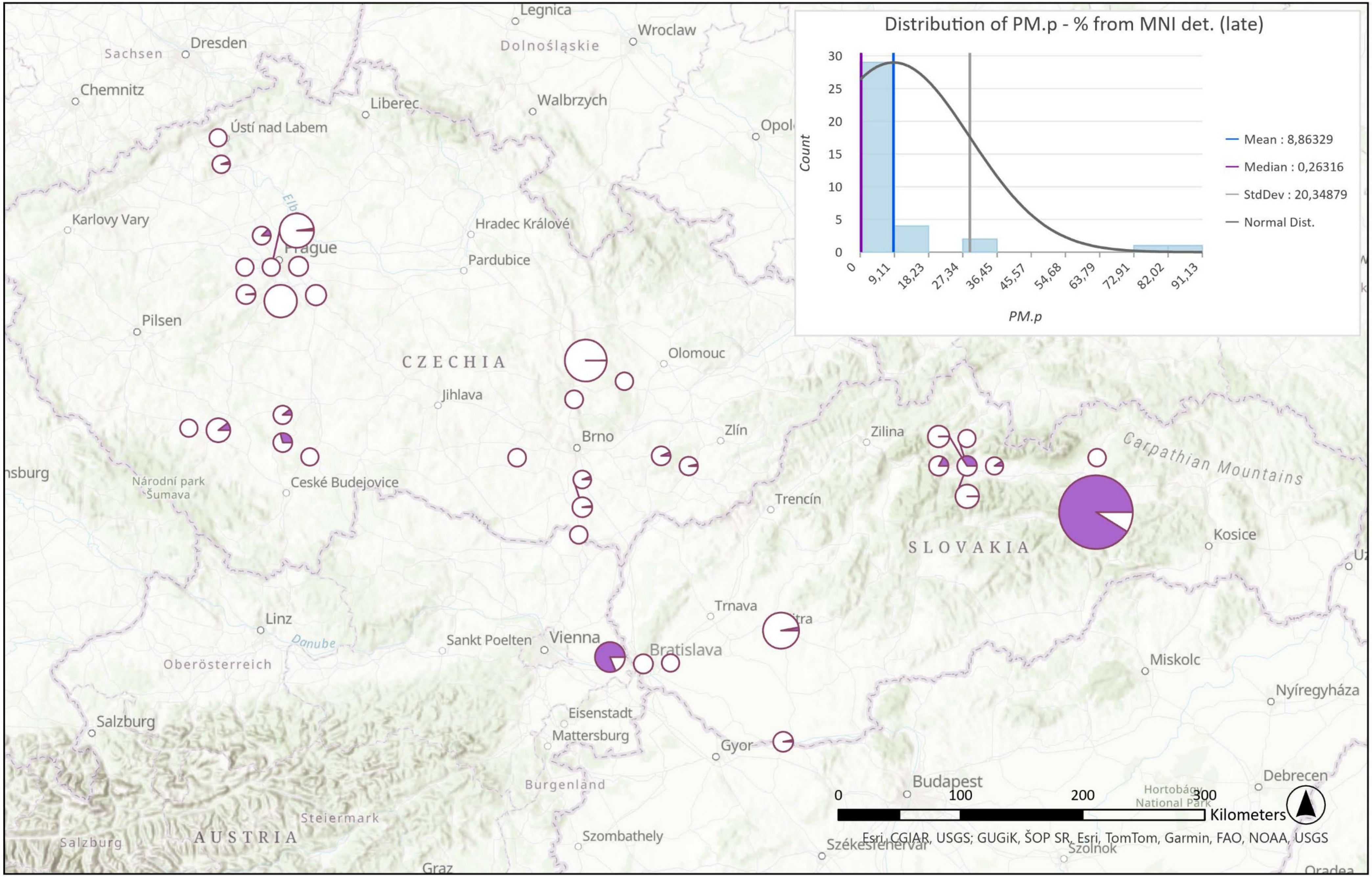

Figure S12. Pie charts for the **% from MNI determined** of millet at sites in the study area (Bohemia, Moravia, Slovakia) during the **late period (180 - 0 BCE/CE).** Chart sizes are normalised by the number of cereal species finds (including fragments). Inset - histogram of the % from the MNI determined values with basic statistics.

Image created with software AcrGIS Pro, www.arcgis.com. Background map: World Topographic Map by © Esri and NASA, NGA, USGS, GUGiK, ŠOP SR, Esri TomTom, Garmin, FAO, METI/NASA
