# Supplementary material for: Multiproxy evidence of millet reliance and selective dietary change during iron age transformation in Central Europe
Source: Sci Rep. 2025 Nov 21;15:41364. doi: 10.1038/s41598-025-25274-z (PMC12638749; doi:10.1038/s41598-025-25274-z)
Supplement: Supplementary file 3 — Supplementary Information 3. [file 41598_2025_25274_MOESM3_ESM.pdf]

Results of Stable Isotope Analysis of Nitrogen and Carbon

Collagen Preparation Procedure

Institute of Archaeology of the CAS, Prague

Collagen was extracted from altogether 86 skeletal elements (79 samples + 7 in house standards) and analysed for $\delta^{13}C$, $\delta^{15}N$, C%, and N% values. Sampled bones were mostly ribs; only in limited number of cases, femurs or humeri were sampled in case ribs were not preserved. Collagen extraction followed Longin [1971], and Bocherens et al. [1997].

Czech Radiocarbon Laboratory
Upon arrival, collagen samples were free dried in the laboratory for 40 hours minimum, and each sample was weighed three times into tin capsules.

Sample Measurement
Measurements were conducted using a Thermo Delta V isotope ratio mass spectrometer coupled with a Flash IRMS elemental analyser.
The mass proportions of nitrogen and carbon were calibrated using glycine (18.66% N, 32.00% C) and IAEA600 (28.85% N, 49.48% C). The abundance of stable nitrogen and carbon isotopes was calibrated relative to VPDB and air using international standards IAEA600, IAEAN1, IAEAN2, and IAEACH3. Reference materials used as control samples included: Casein ($\delta^{15}N = 6.3 \pm 0.3$, $\delta^{13}C = -25.2 \pm 0.2$), USGS89 (pig collagen, $\delta^{15}N = 6.3 \pm 0.1$, $\delta^{13}C = -18.12 \pm 0.1$), STDS (pig collagen, $\delta^{15}N = 4.7 \pm 0.2$, $\delta^{13}C = -13.2 \pm 0.3$), STDBR1 (bovine collagen, $\delta^{15}N = 8.4 \pm 0.7$, $\delta^{13}C = -10.2 \pm 0.3$) [Stabilisotopenanalytik 2018]. Overall analytical uncertainty was established based on measurements of calibration standards, reference materials, and repeated sample measurements [Szpak et al. 2017]. Based on samples analysed multiple times, as well as repeated extractions and measurements of matrix-matched in-house reference materials (modern camel and ibex bone powder), the external reproducibility (1σ) was better than ± 0.29‰ for $\delta^{13}C$, and ± 0.25‰ for $\delta^{15}N$ values.

References:

Bocherens H, Billiou D, Patou-Mathis M, Bonjean D, Otte M, Mariotti A (1997) Paleobiological implications of the isotopic signatures (13C,15N) of fossil mammal collagen in Scladina Cave (Sclayn, Belgium). Quatern Res 48(03), 370–380. https://doi.org/10.1006/qres.1997.1927

Longin R (1971) New method of collagen extraction for radiocarbon dating. Nature, 230, 241–242. doi:10.1038/230241a0

Stabilisotopenanalytik (2018) Intra and inter laboratory reference materials for multi element stable isotope analysis in food authentication. Lebensmittelchemie, 72, 73 – 104.

Szpak P. et al. (2017) Best practices for calibrating and reporting stable isotope measurements in archaeology. Journal of Archaeological Science: Reports, 13, 609 – 616.

Table S12: Mass percentages of N and C, calculated atomic C:N ratio, and $\delta^{15}N$ and $\delta^{13}C$ values in per mil (‰). The result for each sample represents the average of three measurements. Atomic C:N ratio values falling outside the range of 2.7–3.7 may indicate lower collagen quality. Samples marked by * represent in house standards. The overall analytical uncertainties were determined to be ±0.3% for nitrogen mass %, ±0.8% for carbon mass %, ±0.3‰ for $\delta^{15}N$, and ±0.3‰ for $\delta^{13}C$.

Lab. number			%N	%C	C:N	$\delta^{15}N$	$\delta^{13}C$
CRL	24_1866	BLU 4	14.9	42.5	3.3	10.5	-17.8
CRL	24_1867	BLU 6	15.6	44.0	3.3	10.9	-19.0
CRL	24_1868	BLU 8	13.2	31.1	2.8	10.8	-19.5
CRL	24_1869	BLU 9	13.2	31.3	2.8	10.1	-18.4
CRL	24_1873	BLU 15	13.9	44.0	3.7	9.9	-20.8
CRL	24_1874	BLU 16	14.0	43.6	3.6	11.4	-18.3
CRL	24_1875	BLU 17	13.5	31.9	2.7	10.7	-20.1
CRL	24_1876	BLU 19	12.7	40.6	3.7	11.6	-19.6
CRL	24_1877	BLU 21	13.8	42.6	3.6	11.8	-18.8
CRL	24_1878	BLU 22	13.3	41.8	3.7	11.2	-19.1
CRL	24_1879	BLU 24	13.5	41.9	3.6	11.5	-19.3
CRL	24_1880	BLU 26	13.3	41.3	3.6	11.4	-19.3
CRL	24_1881	NEC 3	15.2	42.7	3.3	8.5	-20.4
CRL	24_1882	NEC 5	15.2	41.8	3.2	8.0	-19.5
CRL	24_1883	NEC 7	14.6	39.8	3.2	9.1	-18.5
CRL	24_1884	NEC 9	15.3	42.2	3.2	7.0	-16.9
CRL	24_1885	NEC 11	14.6	41.4	3.3	8.7	-19.4
CRL	24_1886	NEC 12	14.8	41.1	3.2	8.7	-18.9
CRL	24_1887	NEC 18	14.7	40.7	3.2	8.2	-21.4
CRL	24_1888	MAL 12	14.4	42.1	3.4	10.6	-19.8
CRL	24_1889	MAL 15	11.4	38.3	3.9	9.9	-20.9
CRL	24_1891	MAL 17	15.0	43.4	3.4	9.8	-19.8
CRL	24_1892	MAL 27	14.7	46.3	3.7	9.2	-21.3

Lab. number			%N	%C	C:N	$\delta^{15}N$	$\delta^{13}C$
CRL	24_1893	MAL 28	17.4	48.0	3.2	10.4	-18.6
CRL	24_1895	MAL 30	15.7	46.4	3.4	9.6	-19.3
CRL	24_1896	MAL 31	15.7	45.5	3.4	10.4	-20.1
CRL	24_1897	MAL 32	13.7	42.6	3.6	11.0	-19.7
CRL	24_1898	MAL 33	12.6	41.8	3.9	9.8	-21.5
CRL	24_1899	MAL 35	13.1	40.9	3.7	10.5	-19.7
CRL	24_1900	MAL 36	14.8	41.3	3.2	11.2	-18.7
CRL	24_1901	MAL 37	13.5	41.6	3.6	9.7	-18.8
CRL	24_1902	MAL 39	14.1	43.9	3.6	10.7	-17.9
CRL	24_1903	MAL 41	13.6	42.6	3.6	9.6	-19.7
CRL	24_1904	MAL 43	14.4	43.6	3.5	11.0	-19.1
CRL	24_1905	MAL 45	14.8	44.3	3.5	11.5	-19.9
CRL	24_1906	MAL 47	14.4	45.5	3.7	10.7	-18.6
CRL	24_1907	MAL 49	14.4	47.0	3.8	12.0	-19.5
CRL	24_1908	MAL 51	13.3	45.6	4.0	11.6	-19.4
CRL	24_1909	MAL 53	14.8	45.9	3.6	11.4	-19.2
CRL	24_1910	MAL 55	16.2	51.1	3.7	11.9	-18.2
CRL	24_1911	MAL 57	12.6	41.4	3.9	12.4	-20.4
CRL	24_1912	MAL 59	14.3	41.9	3.4	8.8	-21.4
CRL	24_1913	MAL 61	13.2	39.7	3.5	11.1	-20.3
CRL	24_1914	MAL 63	12.4	40.2	3.8	8.2	-21.6
CRL	24_1915	MAL 65	14.1	42.5	3.5	11.6	-19.2
CRL	24_1916	MAL 67	12.5	38.3	3.6	11.9	-19.1
CRL	24_1917	MAL 69	14.8	42.4	3.3	10.1	-19.0
CRL	24_1918	MAL 71	12.6	39.6	3.7	9.0	-22.1
CRL	24_1919	MAL 73	14.9	43.4	3.4	10.1	-19.6
CRL	24_1920	MAL 75	13.3	39.6	3.5	9.6	-21.4
CRL	24_1921	MAL 77	14.8	41.8	3.3	9.4	-19.7

Lab. number			%N	%C	C:N	δ^{15}N	δ^{13}C
CRL	24_1922	MAL 79	13.3	39.1	3.4	9.0	-21.5
CRL	24_1923	MAL 81	13.7	40.8	3.5	8.5	-20.2
CRL	24_1924	MAL 83	15.8	43.5	3.2	9.7	-18.4
CRL	24_1925	MAL 85	14.9	42.4	3.3	10.1	-20.4
CRL	24_1926	MAL 87	15.5	44.0	3.3	9.9	-18.5
CRL	24_1927	MAL 89	15.2	41.2	3.2	8.4	-20.6
CRL	24_1928	MAL 91	15.0	40.2	3.1	8.3	-20.5
CRL	24_1929	MAL 93	12.8	42.2	3.9	10.8	-20.3
CRL	24_1930	MAL 95	11.9	40.2	3.9	10.2	-19.7
CRL	24_1931	MAL 97	12.7	41.4	3.8	11.9	-20.4
CRL	24_1933	MAL 101	13.3	43.0	3.8	11.3	-19.6
CRL	24_1934	MAL 103	12.7	41.7	3.8	10.2	-19.7
CRL	24_1935	MAL 105	12.8	41.5	3.8	11.9	-20.6
CRL	24_1936	MAL 107	12.7	41.7	3.8	9.8	-21.4
CRL	24_1937	MAL 109	11.5	38.6	3.9	9.8	-19.1
CRL	24_1938	MAL 111	12.7	41.2	3.8	10.4	-20.0
CRL	24_1940	LOV 2	11.7	36.3	3.6	9.8	-17.3
CRL	24_1941	LOV 3	13.3	41.0	3.6	10.4	-18.9
CRL	24_1942	LOV 5	12.9	39.5	3.6	11.7	-17.2
CRL	24_1943	LOV 7	12.4	40.1	3.8	10.0	-19.4
CRL	24_1944	LOV 9	12.2	37.7	3.6	10.2	-18.8
CRL	24_1945	LOV 11	12.8	40.6	3.7	10.1	-19.1
CRL	24_1946	LOV 13	12.8	41.1	3.7	11.3	-20.5
CRL	24_1947	LOV 14	13.1	31.7	2.8	10.0	-19.7
CRL	24_1948	LOV 15	11.9	29.3	2.9	9.6	-20.9
CRL	24_1949	LOV 19	11.1	27.5	2.9	9.8	-21.5
CRL	24_1950	LOV 21	12.7	29.9	2.8	9.0	-18.9
CRL	24_1951	LOV 23	13.2	31.1	2.8	10.5	-19.6

Lab. number			%N	%C	C:N	δ^{15}N	δ^{13}C
CRL	24_1955	LOV 30	13.0	30.4	2.7	9.9	-19.2
CRL	24_1956*	IBEX 2	13.5	44.2	3.8	7.6	-25.5
CRL	24_1957*	IBEX 3	13.5	43.4	3.8	7.6	-25.2
CRL	24_1958*	IBEX 4	12.6	30.5	2.8	7.1	-25.7
CRL	24_1959*	IBEX 5	12.9	32.0	2.9	7.1	-26.0
CRL	24_1960*	CAMEL 2	13.8	42.7	3.6	10.1	-14.2
CRL	24_1961*	CAMEL 3	14.3	42.7	3.5	10.1	-13.7
CRL	24_1962*	CAMEL 5	13.4	40.6	3.5	10.4	-14.3
