# Supplementary material for: Multiproxy evidence of millet reliance and selective dietary change during iron age transformation in Central Europe
Source: Sci Rep. 2025 Nov 21;15:41364. doi: 10.1038/s41598-025-25274-z (PMC12638749; doi:10.1038/s41598-025-25274-z)
Supplement: Supplementary file 4 — Supplementary Information 4. [file 41598_2025_25274_MOESM4_ESM.pdf]

SEM SI_analysis

Classification of the population buried at middle La Tène cemeteries (400 – 180 BCE) for the $\delta^{13}C$ isotopic analysis and statistical evaluation of isotopic data.

Based on previous studies noting the C_4 isotopic shift and other published data, a classification framework for the burial populations from La Tène cemeteries was developed to address the central research question: what were the underlying causes of the increased millet consumption in La Tène diets during the 3rd century BCE?
To determine whether the observed $\delta^{13}C$ differences—indicative of varying plant intake—were typical dietary features of the La Tène period linked to sex or age, or whether they reflected broader socio-economic factors such as status or individual roles, samples with available anthropological determinations of biological sex and age were analysed for $\delta^{13}C$ values across the 4th and 3rd centuries BCE. Previous research (eg. Moghaddam et al., 2016; Vytlačil et al., 2024; Kertés, 2025) has shown no significant influence of sex or age on isotopic diet composition, related to carbon, over the middle La Tène period (400 – 180 BCE). To test this finding in the present study, all samples with demographic data were evaluated accordingly.
A major limitation of this approach was the incomplete and often unreliable nature of the sex and age determinations, primarily due to poor preservation of anthropological material. As a result, much of the demographic dataset had to be excluded from analysis. Only selected samples from the cemeteries of Prosmyky, Jinonice, Ruzyně-Jiviny, Radovesice, and Tišice have recently undergone re-evaluation of biological sex using peptide analysis, although these results remain unpublished.

The following age categories were established for the analysis:

- **Infants (0–7 years):** This group includes individuals estimated to be younger than 7 years. Although a subset of samples (n = 7) was aged below 3–4 years and could be influenced by weaning effects, the sample size was too small for a separate category. An additional four individuals were estimated to be between 1 and 6 years. Therefore, all individuals under 7 years of age were grouped together.
- **7–25 years:** This broad category encompasses later childhood and adolescence. While a narrower year range would be preferable, the available age estimates in published reports did not permit such refinement.
- **26–45 years:** Represents the adult population.
- **46+ years:** Represents the elderly population.
- **Indeterminate**: Samples lacking reliable age determination.

The biological sex categories were defined as:

- **Males**: Individuals identified as biologically male through anthropological assessment or biomolecular methods (e.g. peptide analysis, aDNA).

- **Females**: Individuals identified as biologically female through anthropological assessment or biomolecular methods.
- **Indeterminate**: Samples for which biological sex could not be determined.

Following the analysis of demographic variables, the burial population was classified according to the **nature and character of grave goods.** This classification reflects the traditional binary pattern dominating the interpretation of La Tène cemeteries—graves containing warrior equipment versus those featuring rich female attire. In Iron Age archaeology, such classification is a long-standing and widely applied convention, evident in major syntheses and case studies (Hodson, 1968; Holodňák, 1988; Krämer, 1985; Ramsl, 2002; Venclová et al., 2013; Waldhauser & others, 1978, 1987). This simple classification was supported by a large-scale quantitative study of Upper Seine Basin cemeteries, based on statistical analysis of several hundred broadly comparable Late Iron Age graves (Evans, 2004). Although the categories are not exclusive and allow for mixed or neutral cases, they consistently reveal patterned associations between artefacts and social identity.

However, recent theoretical and empirical studies caution against equating such archaeological "gender" assignments with biological sex. As Gaydarska (Gaydarska et al., 2023) emphasizes, bioarchaeologists determine sex from skeletal remains, while archaeologists assign gender based on grave goods, and only by distinguishing the two can the interplay between biology and cultural expression be understood. Recent advances in analytical techniques, such as aDNA and amelogenin peptide analysis (Buonasera et al., 2020), have significantly improved the accuracy of biological sex determination in La Tène burials. This has enabled more precise correlations between biological sex and burial assemblages, while also revealing deviations from traditional binary gender patterns. Several recent cases have challenged the conventional association between biological sex and specific grave goods, suggesting more fluid or socially constructed gender roles during the La Tène period. Although most of this data remains unpublished (safe for e.g. Patterson & al, 2022), it is increasingly clear that rich grave assemblages are not rigidly tied to biological sex. Although archaeological sex/gender assignments cannot be accepted with the same evidentiary category as biological determinations, they nonetheless reflect meaningful cultural signals. Grave assemblages—whether including warrior accoutrements, rich jewellery, or hybrid combinations—carry significant symbolic weight and can inform us about social roles, identity expressions, and communicative intent in mortuary contexts. As such, these cultural determinations must be treated with analytical seriousness, much like biological assessments, albeit interpreted within a broader, socially informed framework. In light of these findings, the present classification of social groups at La Tène cemeteries does not rely solely on biological sex. Instead, it is based on the types and combinations of grave goods, acknowledging the diversity and complexity of social identity in the archaeological record and the grave-goods based classification represents a meaningful framework of social classification analytically distinct from both biological sex and socially hierarchical determination of the individual categories.

Overview of the types of grave goods and burial attires at La Tène period cemeteries in central Europe

Warrior graves typically include a complete or near-complete set of weapons such as swords, lances, shields, and metal belts (Ramsl, 2018). These are often accompanied by costume elements and personal ornaments like bronze or iron brooches, bracelets, and armrings. Notably, no infant or child burials with weapons have been recorded. In contrast, the graves of unarmed males are harder to distinguish based on grave goods alone, as they often resemble female graves. When determinable, male graves may include one or two brooches and asymmetrically worn ring jewellery, crafted from bronze, iron, or sapropelite. Some male burials entirely lack grave goods or personal objects.

Female graves show greater variability in costume composition, which may reflect social status, cultural customs, or personal expression. The most elaborate female burials contain multiple bronze brooches, paired jewellery (e.g. bracelets, armrings, and especially anklets), and in some cases, bronze torcs. Paired jewellery is a particularly diagnostic feature of richly adorned female graves. In the latest chronological phases, complex bronze belts—often decorated with red enamel—became common, and the first glass ornaments, primarily bracelets and armrings, also appear.

Less elaborate female costumes are more variable, typically lacking paired anklets and comprising simpler versions of the complete attire. A typical La Tène female burial may include one or two brooches, belt rings, and a variable number of bronze bracelets. Iron and sapropelite ornaments are also common across the full range of female graves, including the richest ones. These material choices appear to reflect cultural norms rather than social status. As with males, female burials without preserved costume parts are not uncommon.

Unlike weapon burials, some children's graves contain relatively rich jewellery assemblages, though these often deviate from standard adult female attire. They may include anklets and asymmetrical or repurposed jewellery, but most child burials lack any grave goods, complicating interpretations of their sex and social role.

In addition to costume elements and personal ornaments, other categories of grave goods were found in La Tène burials, varying by region and chronological phase. In later periods, particularly in Bohemia, Moravia, and Slovakia, ceramic vessels became common inclusions in graves, irrespective of the individual's biological sex or the richness of their attire. These graves typically contain one vessel and one or two items of ring jewellery or a brooch. In the eastern parts of the study area, animal offerings were also frequent, though usually restricted to the wealthiest burials.

Classifying the burial population based on grave goods is inherently complex, given the number and diversity of objects and combinations present (Bujna, 1982). For the purpose of this study, a simplified classification approach was adopted—distinguishing the richest burial categories from the remainder of the population—since further subdivision within the "common grave goods" category would risk introducing classification bias[1].

[1] This classification was already used in Kertés, 2022, but was performed here again with the revision of the original data and with inclusion of newly analysed data. Based on the results presented in the thesis, the “poorer graves” and “no grave goods” category were merged into one.

Burials recorded without grave goods were also excluded from separate analysis, as the absence of preserved organic materials may have led to underrepresentation of grave furnishings, resulting in misclassification.

Accordingly, the **classification of burial assemblages**—as a proxy for the individual's social role or status—was structured into the following groups:

Classification of Grave Goods categories (Social Groups) in La Tène Cemeteries in Bohemia, Moravia, and Slovakia:

- **Group 1A** – *Graves with warrior equipment*:
  Burials containing all or most of the following: sword, lance, shield, belt, and costume elements such as brooches, bracelets, or armrings. In some regions (especially Moravia and Slovakia) also ceramic vessels and animal offerings can be present.

- **Group 1B** – *Graves with rich bronze jewellery*:
  Burials featuring elaborate adornments, including torcs, paired bracelets and anklets, armrings, belts, multiple brooches, and occasionally finger rings. In some regions (especially Moravia and Slovakia) also ceramic vessels and animal offerings can be present.

- **Group 1S** – *Graves with specific equipment or observations:*
  Burials assembled under this category represent cases where the biological sex determinations returned opposite results than expected for the given type of burial attire. It includes females buried exclusively with weapons or males in completely female associated costume. This category includes several instances where such observations could be made and it serves for the potential excluding of outlying values should these individuals show specific dietary patterns. This group also includes instances with anomalous grave goods (such as rare cases of female costume combined with weaponry).

- **Group 2** – *Graves with simpler, more modest or no preserved grave goods or personal objects*:
  Burials with simpler assemblages, typically comprising a variable number of ring jewellery, brooches, and belts, in some regions also ceramics or other objects, or no preserved grave goods at all.

Dating of samples within La Tène period relative chronology (Venclová et al., 2013)

- **4th century BCE** (400 - 300/290): LT B1 (400 – 350 BCE), LT B2a (350-300 BCE)
- **3rd century BCE** (300/290 - 200/180 BC): LT B2b (300-260 BCE), LTB2b-C1 (260-230 BCE), LT C1 (230-200/180 BCE)

Analysis

A total of 427 collagen samples from La Tène cemeteries in Bohemia, Moravia, and Slovakia (Table S9) were classified according to the defined demographic and grave goods (social group) categories. All 79 data from current measurements (Table 9, lines 340-419) were included in the analysis due to consistency, however, it is acknowledged, that samples outside the C:N ratio between 2.7-3.7 may have lower collagen quality (Table S12). Samples with indeterminate demographic, chronological or burial data were excluded from the analyses that evaluated particular sub-datasets. Isotopic patterns for the individual sites are shown in Fig. S1.

Note: all $\delta^{13}C$ values are given in ‰ and are relative to the VPDB standard.

1. <u>Chronological development (the whole population)</u>

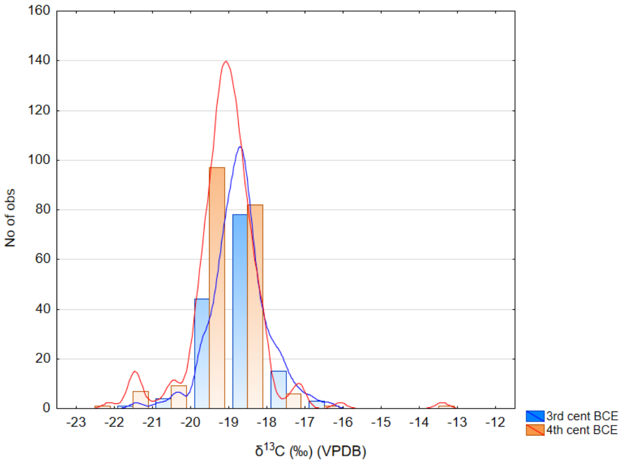

A

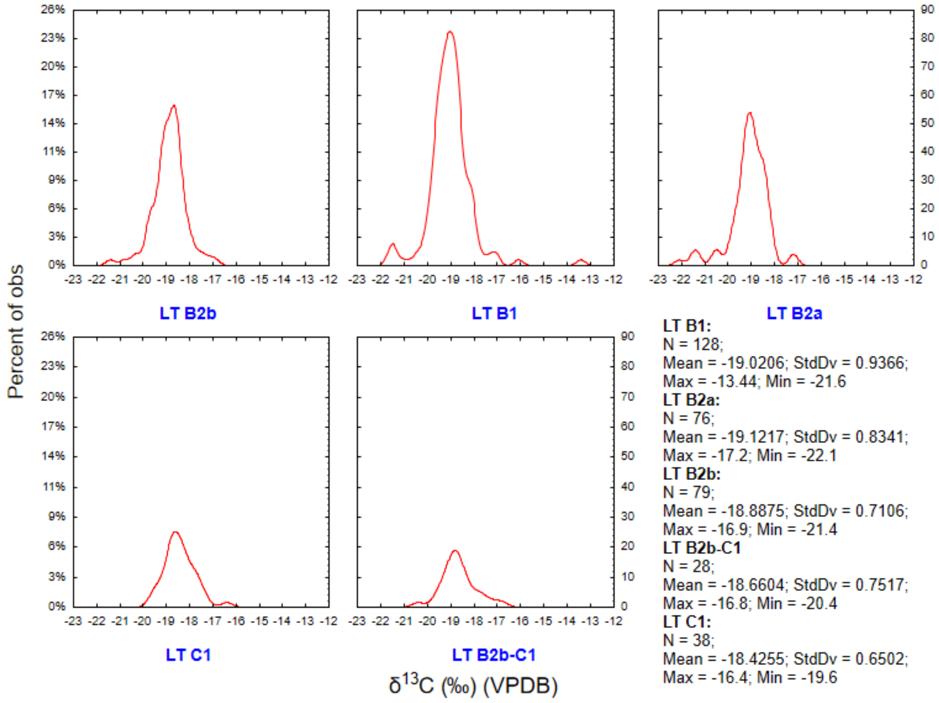

B

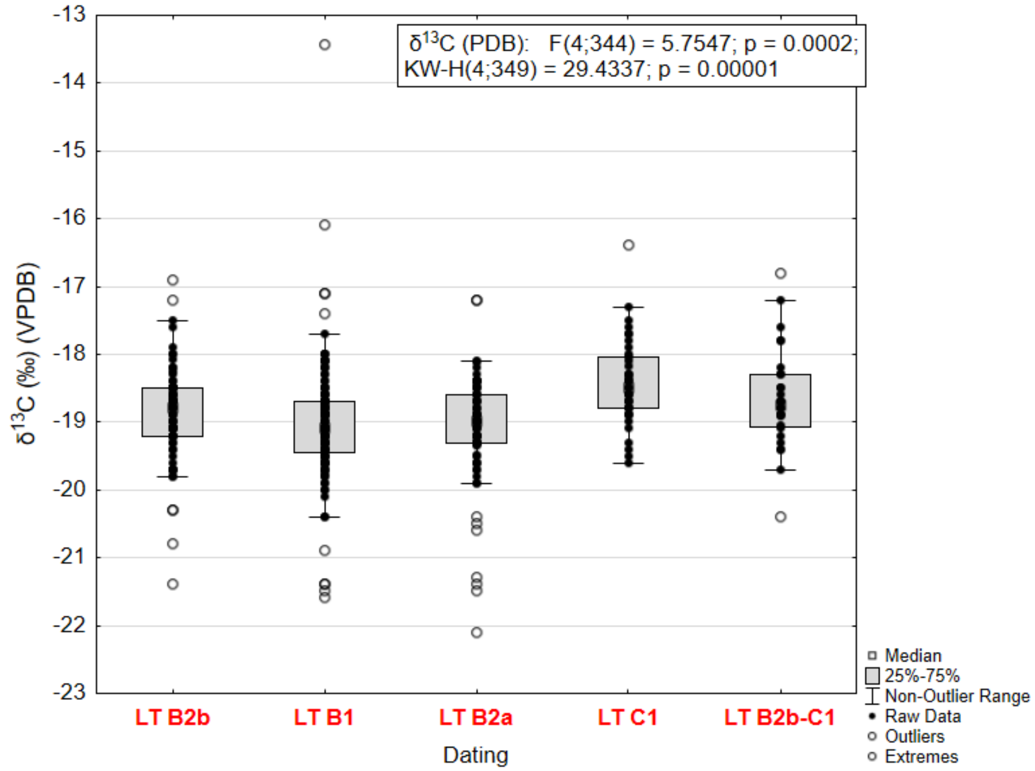

C

Figure S13. Histogram and gaussian kernel plots (see Methods section in the main text) evaluating the chronological development. A – according to centuries; B, C – according to individual relative chronological phases.

Categorisation of all samples (without further subdivision) according to chronological variables revealed that there is indeed a statistically significant difference between chronological periods both at the century level and at the level of relative chronological phases (Fig. S13, Table S13, S14).

variable	Mann-Whitney U Test (w/ continuity correction) (Bohemia+Moravia+Slovakia in C_N_S_results2)<br>By variable cent<br>Marked tests are significant at p <.05000								
	Rank Sum 3rd cent	Rank Sum 4th cent	U	Z	p-value	Z adjusted	p-value	Valid N 3rd cent	Valid N 4th cent
$\delta^{13}C$ (VPDB)	29948.00	30778.00	10275.00	4.827051	0.000001	4.831577	0.000001	146	202

Table S13. Mann-Whitney U test of the difference between the 4th century BCE and the 3rd century BCE.

Depend.: $\delta^{13}C$ (VPDB)	Multiple Comparisons p values (2-tailed); $\delta^{13}C$ (VPDB)<br>Independent (grouping) variable: **RELATIVE PHASES**<br>Kruskal-Wallis test: H (4, N= 348) =34.97737 p =.0000				
	**LT B2b**	**LT B1**	**LT B2a**	**LT C1**	**LT B2b-C1**
**LT B2b**		0.333970	0.963256	0.006905	1.000000
**LT B1**	0.333970		1.000000	0.000001	0.078096
**LT B2a**	0.963256	1.000000		0.000017	0.193820
**LT C1**	0.006905	0.000001	0.000017		0.960006
**LT B2b-C1**	1.000000	0.078096	0.193820	0.960006	

Table S14. Kruskal-Wallis ANOVA test of the difference between the relative chronological phases.

2. Regional variability (the whole population)

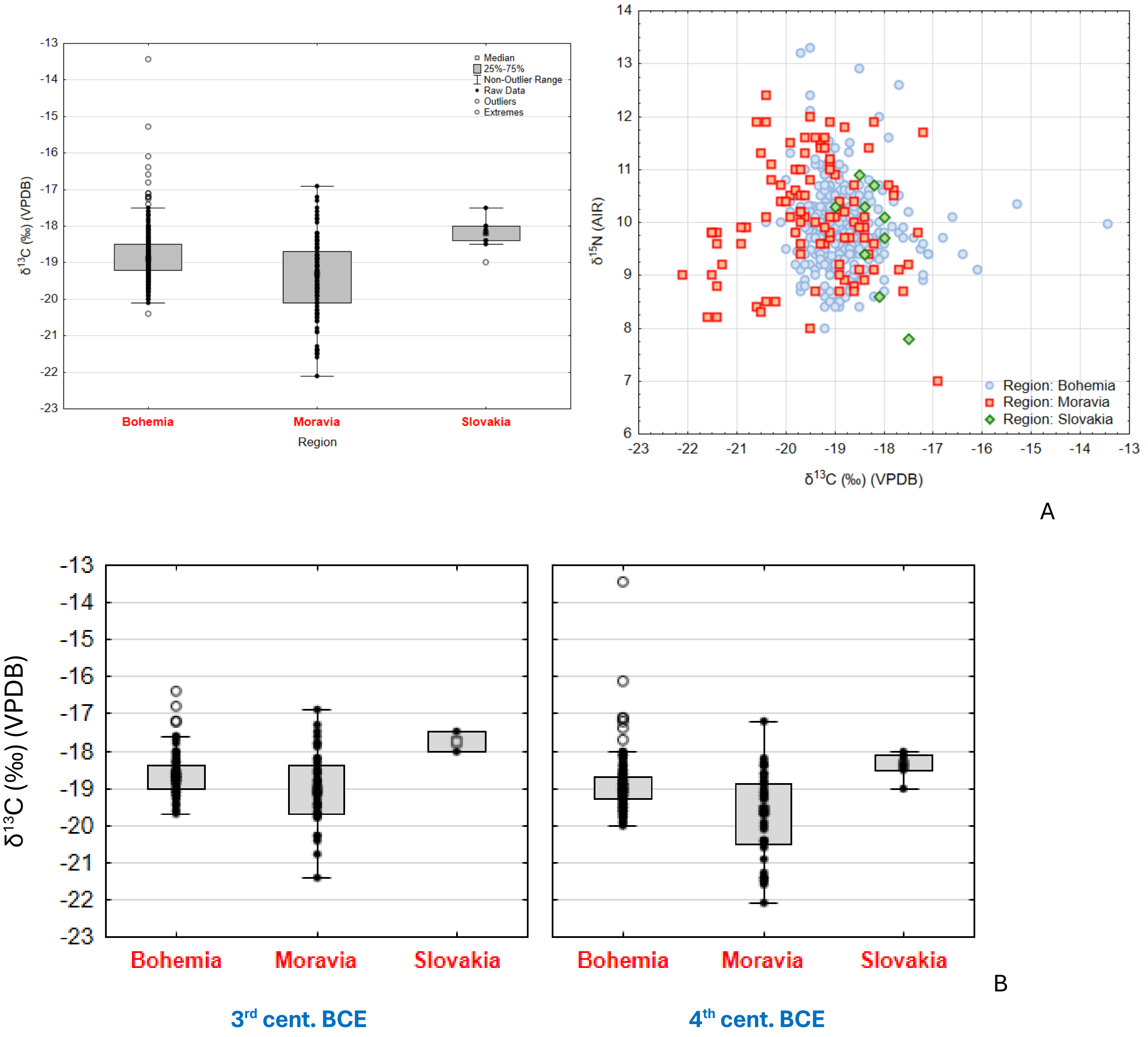

Figure S14. A - Box plots of the $\delta^{13}C$ values of the individual regions. B - Box plots of the $\delta^{13}C$ values of the individual regions according to centuries.

Depend.: $\delta^{13}C$ (VPDB)	Multiple Comparisons p values (2-tailed); $\delta^{13}C$ (VPDB)<br>Independent (grouping) variable: **REGION**<br>Kruskal-Wallis test: H (2, N= 429) =36.68891 p =.0000			
	**Bohemia**	**Moravia**	**Slovakia**	
**Bohemia**		0.000002	0.007697	
**Moravia**	0.000002		0.000015	
**Slovakia**	0.007697	0.000015		

Table S15. Kruskal-Wallis ANOVA test of the difference between the regions.

Depend.: $\delta^{13}C$ (VPDB)	**century=4th**<br>Multiple Comparisons p values (2-tailed); $\delta^{13}C$ (VPDB)<br>Independent (grouping) variable: **REGION**<br>Kruskal-Wallis test: H (2, N= 198) =25.07526 p =.0000			Depend.: $\delta^{13}C$ (VPDB)	**century=3rd**<br>Multiple Comparisons p values (2-tailed); $\delta^{13}C$ (VPDB)<br>Independent (grouping) variable: **REGION**<br>Kruskal-Wallis test: H (2, N= 142) =10.15063 p =.0062		
	**Bohemia**	**Moravia**	**Slovakia**		**Bohemia**	**Moravia**	**Slovakia**
**Bohemia**		0.000253	0.021646	**Bohemia**		0.043239	0.197965
**Moravia**	0.000253		0.000071	**Moravia**	0.043239		0.044310
**Slovakia**	0.021646	0.000071		**Slovakia**	0.197965	0.044310	

Table S16. Kruskal-Wallis ANOVA test of the difference between the regions and centuries.

Categorisation of all samples (without further subdivision) according to geographical variables revealed that there is a statistically significant difference between individual regions both in general and within the individual centuries (Fig. S14, Table S15, S16). The difference is still significant when the data are categorised by relative chronological phases (Fig. S15, Table S17). The differences tend to show between both the earlier and later phases (Bohemia), or between only the earliest and the latest phase (Moravia). The analysis did not perform for Slovakia due to low sample count.

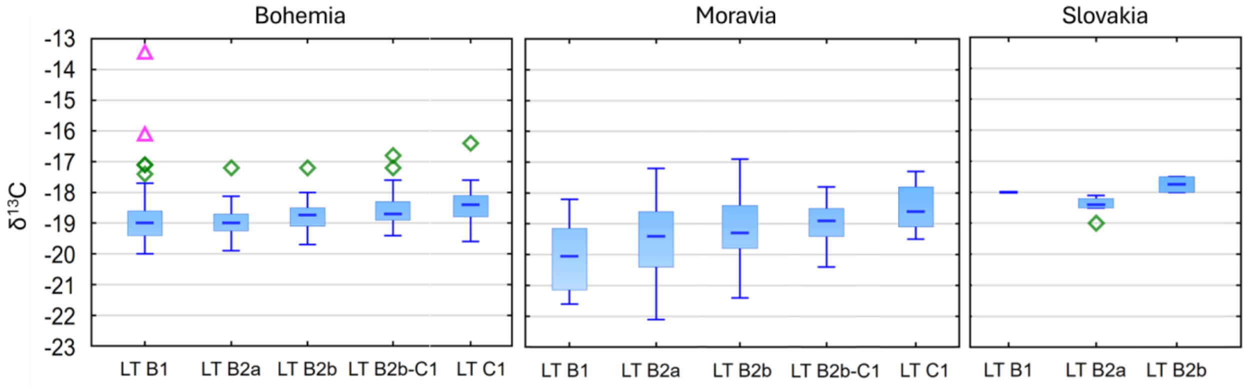

Figure S15. Development of $\delta^{13}C$ (‰) (VPDB) values per regions and phases.

Depend.: $\delta^{13}C$ (VPDB)	Region=**BOHEMIA**<br>Multiple Comparisons p values (2-tailed); δ13C (VPDB)<br>Independent (grouping) variable: Dating<br>Kruskal-Wallis test: H (4, N= 255) =30.84161 p =.0000				
	**LT B2b**	**LT B1**	**LT B2a**	**LT C1**	**LT B2b-C1**
**LT B2b**		0.356245	0.352578	0.086232	1.000000
**LT B1**	0.356245		1.000000	0.000048	0.035030
**LT B2a**	0.352578	1.000000		0.000159	0.032884
**LT C1**	0.086232	0.000048	0.000159		1.000000
**LT B2b-C1**	1.000000	0.035030	0.032884	1.000000	
Depend.: $\delta^{13}C$ (VPDB)	Region=**MORAVIA**<br>Multiple Comparisons p values (2-tailed); δ13C (VPDB)<br>Independent (grouping) variable: Dating<br>Kruskal-Wallis test: H (4, N= 85) =13.37127 p =.0096				
	**LT B2b**	**LT B1**	**LT B2a**	**LT C1**	**LT B2b-C1**
**LT B2b**		0.284489	1.000000	0.804911	1.000000
**LT B1**	0.284489		1.000000	0.007761	0.149501
**LT B2a**	1.000000	1.000000		0.218016	1.000000
**LT C1**	0.804911	0.007761	0.218016		1.000000
**LT B2b-C1**	1.000000	0.149501	1.000000	1.000000	
Depend.: $\delta^{13}C$ (VPDB)	Region=**SLOVAKIA**<br>Multiple Comparisons p values (2-tailed); δ13C (VPDB)<br>Independent (grouping) variable: Dating<br>Kruskal-Wallis test: H (4, N= 9) =0.000000 p =1.000				
	**LT B2b**	**LT B1**	**LT B2a**	**LT C1**	**LT B2b-C1**
**LT B2b**		1.000000	0.336480		
**LT B1**	1.000000		1.000000		
**LT B2a**	0.336480	1.000000			
**LT C1**					
**LT B2b-C1**					

Table S17. Kruskal-Wallis ANOVA test of the individual regions and chronological phases.

3. <u>Results of the demographic categories</u>

3.1. Age groups

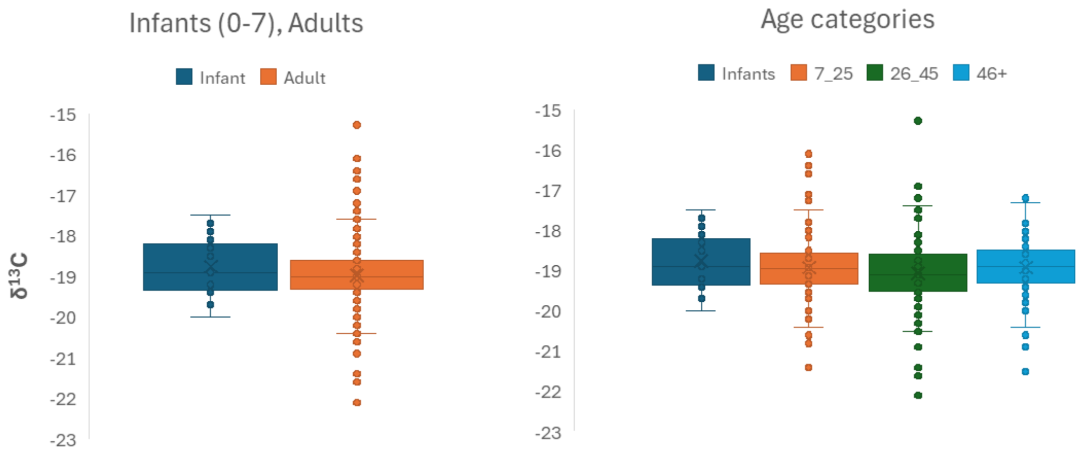

Figure S16. Box plots for individual age groups of all burials with determined age.

The results (Fig. S16) indicate that age categories were not a significant factor influencing dietary variation. This was confirmed by a Kruskal–Wallis ANOVA test ($p = 0.3611$; Table S18).

**Depend.: $\delta^{13}C$ (VPDB)**	Kruskal-Wallis ANOVA by Ranks; Independent (grouping) variable: category<br>Kruskal-Wallis test: $H(3, N = 344) = 3.204871$<br>p =.3611			
	Code	Valid (N)	Sum of (Ranks)	Mean (Rank)
**Infant**	1	15	2903.00	193.5333
**7-25**	2	98	17293.00	176.4592
**26-45**	3	146	23670.00	162.1233
**46+**	4	85	15474.00	182.0471

Table S18. Kruskal-Wallis ANOVA test of the age categories.

Comparable results were obtained when age categories were evaluated separately for the 4th and 3rd centuries BCE (Fig. S17). As in the previous analysis, no statistically significant differences were observed (Kruskal–Wallis ANOVA, $p = 0.1669$ and $p = 0.1219$, respectively; Table S19). Evaluation by finer relative chronological phases was not feasible due to the low number of samples within individual categories.

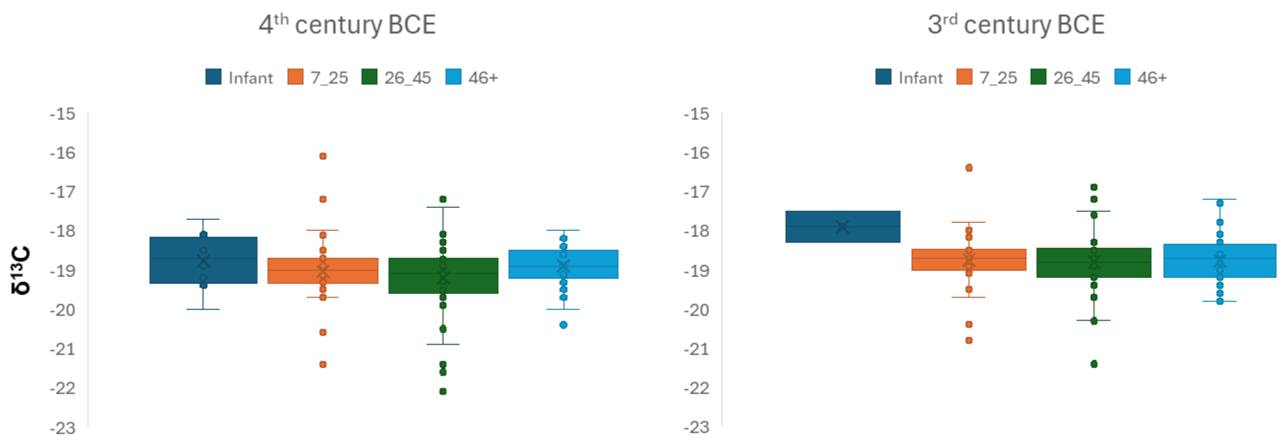

Figure S17. Box plots for individual age groups of all burials with determined age according to centuries BCE.

**Depend.: $\delta^{13}C$ (PDB)**	Kruskal-Wallis ANOVA by Ranks; $\delta^{13}C$ (PDB) (age-**4th century BCE** in C+N_data) Independent (grouping) variable: category Kruskal-Wallis test: H (3, N= 163) =5.067387 p =.1669				**Depend.: $\delta^{13}C$ (PDB)**	Kruskal-Wallis ANOVA by Ranks; $\delta^{13}C$ (PDB) (age-**3th century BCE** in C+N_data) Independent (grouping) variable: category Kruskal-Wallis test: H (3, N= 118) =5.796860 p =.1219			
	Code	Valid (N)	Sum of (Ranks)	Mean (Rank)		Code	Valid (N)	Sum of (Ranks)	Mean (Rank)
**Infant**	1	8	791.000	98.87500	**Infant**	1	3	312.000	104.0000
**7-25**	2	44	3598.500	81.78409	**7-25**	2	34	2105.500	61.9265
**26-45**	3	78	5855.000	75.06410	**26-45**	3	45	2526.000	56.1333
**46+**	4	33	3121.500	94.59091	**46+**	4	36	2077.500	57.7083

Table S19. Kruskal-Wallis ANOVA test of the age categories according to the centuries BCE. Note only three samples in the category "Infant" in the 3rd century BCE.

There is a difference between an Infant category and the rest in the plot for the 3rd century BCE. However, in this instance, the group contains only three samples, so the observed difference is not statistically robust.

3.2. Biological sex groups

The following analysis included all samples except the Infant category (0-7 years). The results (Fig. S18) indicate that, when considering the whole period of the using of cemeteries, biological sex was also not a significant factor influencing dietary patterns. This finding was supported by a Mann–Whitney U test (p = 0.8012; Table S20).

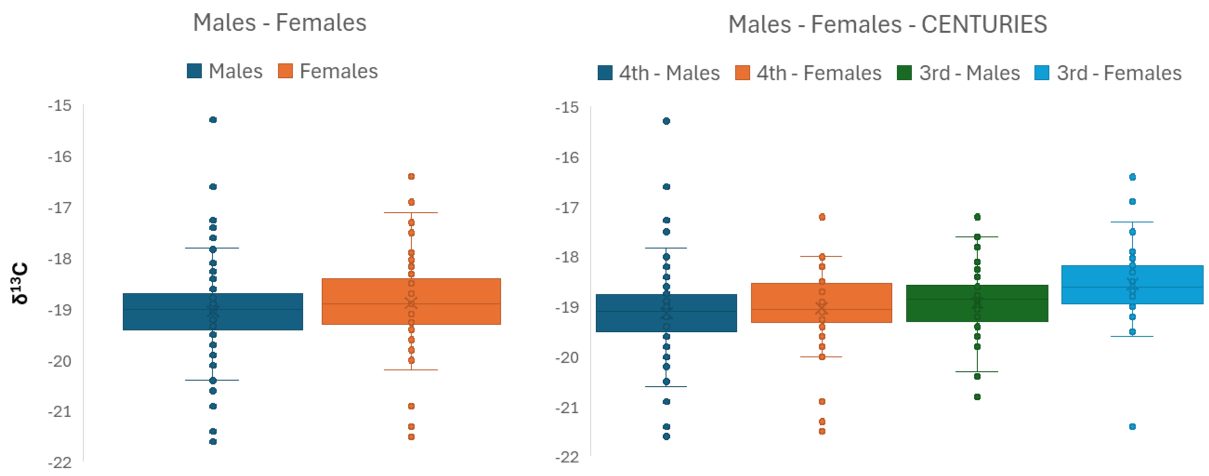

Figure S18. Box plots for male and female groups of all burials with determined sex category (left), and the same data displayed according to biological sex and century BCE.

variable	Mann-Whitney U Test (w/ continuity correction) (male-female in C+N_data)<br>By variable Sex<br>Marked tests are significant at p <.05000									
	Rank Sum (F)	Rank Sum (F)	U	Z	p-value	Z (adjusted)	p-value	Valid N (F)	Valid N (F)	2*1sided (exact p)
$\delta^{13}C$ (VPDB)	6504.000	2011.000	1454.000	-0.25142	0.801487	-0.251687	0.801283	100	30	0.802509

Table S20. Mann-Whitney U test of the biological sex categories.

However, when the data are analysed by chronology, a statistically significant difference between males and females emerges in the 3rd century BCE (Mann–Whitney U test, p = 0.0076; Fig. S18, Table S21).

variable	Mann-Whitney U Test (w/ continuity correction) **(4th century BCE)**									
	Rank Sum (F)	Rank Sum (F)	U	Z	p-value	Z (adjusted)	p-value	Valid N (F)	Valid N (F)	2*1sided (exact p)
$\delta^{13}C$ (VPDB)	2069.500	856.5000	416.5000	-1.49348	0.135312	-1.49552	0.134780	57	19	0.134612
variable	Mann-Whitney U Test (w/ continuity correction) **(3rd century BCE)**									
	Rank Sum (F)	Rank Sum (F)	U	Z	p-value	Z (adjusted)	p-value	Valid N (F)	Valid N (F)	2*1sided (exact p)
$\delta^{13}C$ (VPDB)	900.5000	134.5000	79.50000	2.593590	0.009498	2.597872	0.009381	35	10	0.007606

Table S21. Mann-Whitney U test of the biological sex categories in the 4th century BCE and the 3rd century BCE, respectively.

When breaking the chronological groups into shorter periods classified according to relative chronological phases, it can be seen, that the presumed development is somewhat irregular (Fig. S19). Female carbon values are more elevated during the chronologically non-continuous phases of LT B2b and LT C1. The statistical evaluation shows the lack of statistical significancy in the development for men, and the statistically significant difference between phases LT B1 and LT B2a (early phase – 4th century BC) and phase LT C1 (the end of the later period) for females (Table S22).

It can also be seen that many samples actually lack the biological sex determination (visible as the "indet." category) which can introduce bias into the analysis of the chronological development. Therefore, even if the tendency of higher carbon values for biological females is visible in the current dataset, the result should be regarded as tentative and state-of-the-art-data dependent.

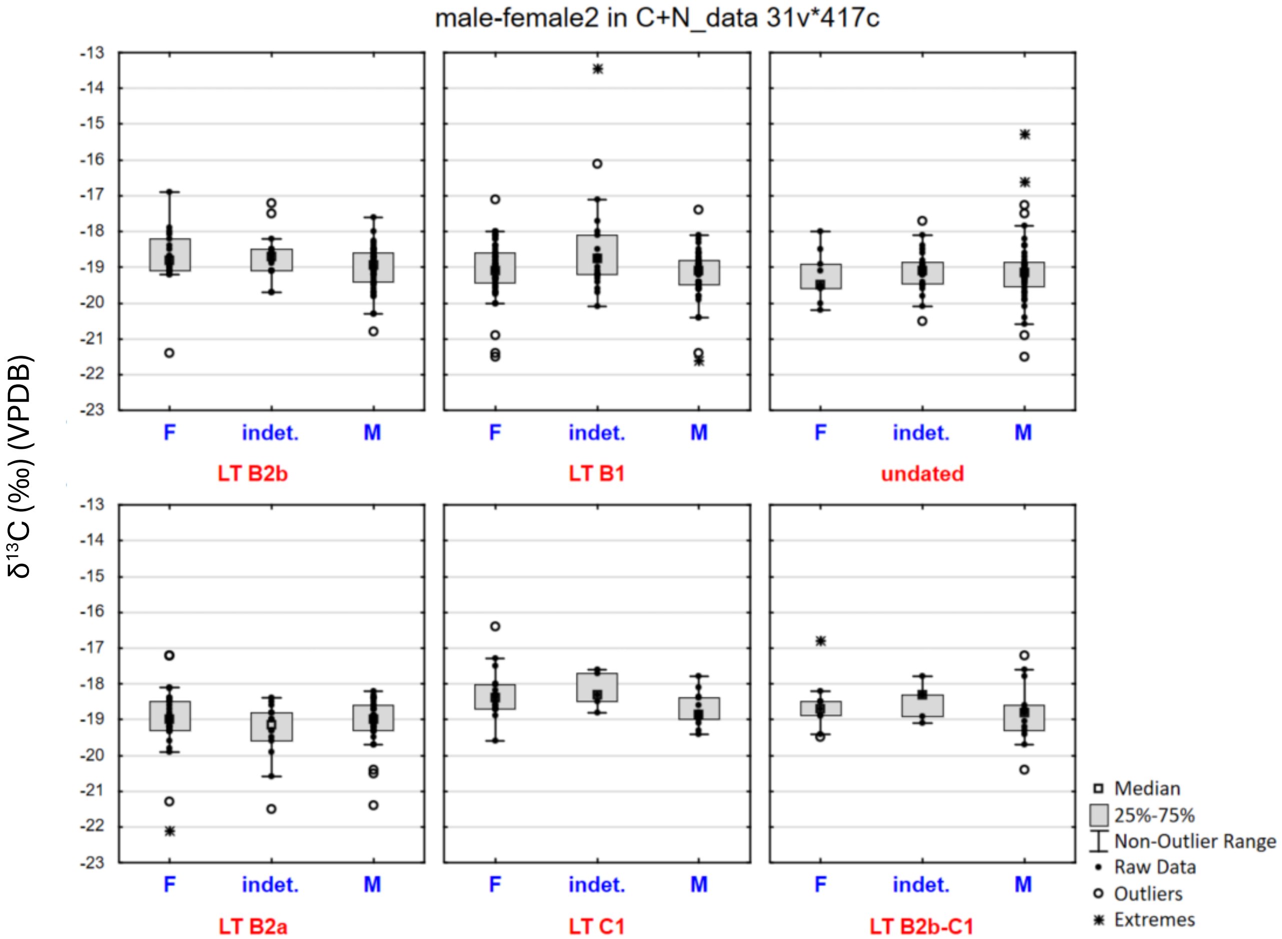

Figure S19. Evolution of male and female carbon values during individual chronological phases. Note the indetermined category ("indet."). The plots do not follow chronological order (correct: LT B1, LT B2a, LT B2b, LT B2b-C1, LT C1).

Depend.: **δ^{13}C (VPDB)**	Multiple Comparisons p values (2-tailed); "**MALES**"<br>Independent (grouping) variable: Dating<br>Kruskal-Wallis test: H (5, N= 198) =8.460876 p =.1326					
	LT B2b (R:105.45)	LT B1 (R:88.290)	undated (R:89.477)	LT B2a (R:103.46)	LT C1 (R:128.61)	LT B2b-C1 (R:114.46)
LT B2b		1.000000	1.000000	1.000000	1.000000	1.000000
LT B1	1.000000		1.000000	1.000000	0.299548	1.000000
undated	1.000000	1.000000		1.000000	0.390774	1.000000
LT B2a	1.000000	1.000000	1.000000		1.000000	1.000000
LT C1	1.000000	0.299548	0.390774	1.000000		1.000000

LT B2b-C1	1.000000	1.000000	1.000000	1.000000	1.000000	
	Multiple Comparisons p values (2-tailed); "**FEMALES**"<br>Independent (grouping) variable: Dating<br>Kruskal-Wallis test: H (5, N= 130) =17.84986 p =.0031					
Depend.: **δ^{13}C (VPDB)**	LT B2b (R:75.000)	LT B1 (R:57.032)	undated (R:44.833)	LT B2a (R:59.345)	LT C1 (R:94.618)	LT B2b-C1 (R:75.167)
LT B2b		1.000000	0.717364	1.000000	1.000000	1.000000
LT B1	1.000000		1.000000	1.000000	0.006346	1.000000
undated	0.717364	1.000000		1.000000	0.020204	1.000000
LT B2a	1.000000	1.000000	1.000000		0.032622	1.000000
LT C1	1.000000	0.006346	0.020204	0.032622		1.000000
LT B2b-C1	1.000000	1.000000	1.000000	1.000000	1.000000	

Table S22. Kruskal-Wallis ANOVA test of the biological sex categories according to relative chronological phases. First table is for males, second for females.

4. Results of the social groups (=archaeological) categories

The dataset included all samples except the Infant category (0-7 years). It was categorised by grave goods into individual “social group” categories and was first tested against both the earlier and later phases (the 4th and 3rd centuries BC, respectively). The results (Fig. S20, S21, Table S23) highlight in particular the distinct position of group 1S, which also underwent the most pronounced change between the two periods, which has been also confirmed as statistically distinctive (Table S23).

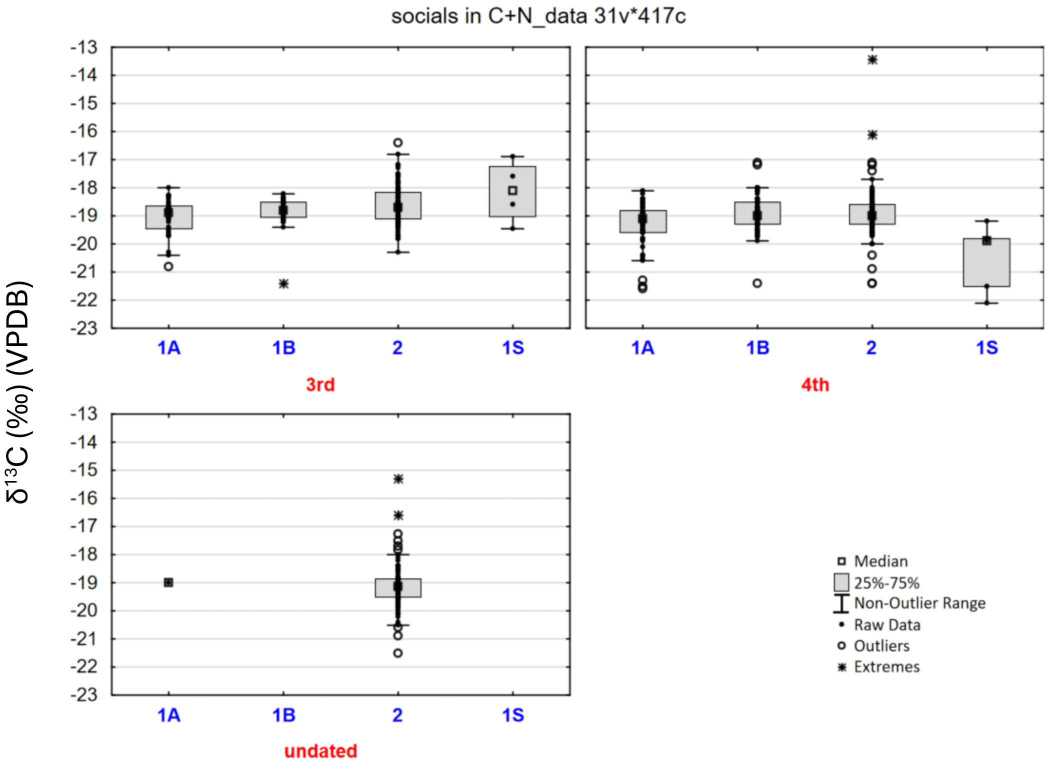

Figure S20. Social group categories compared against each other during individual centuries.

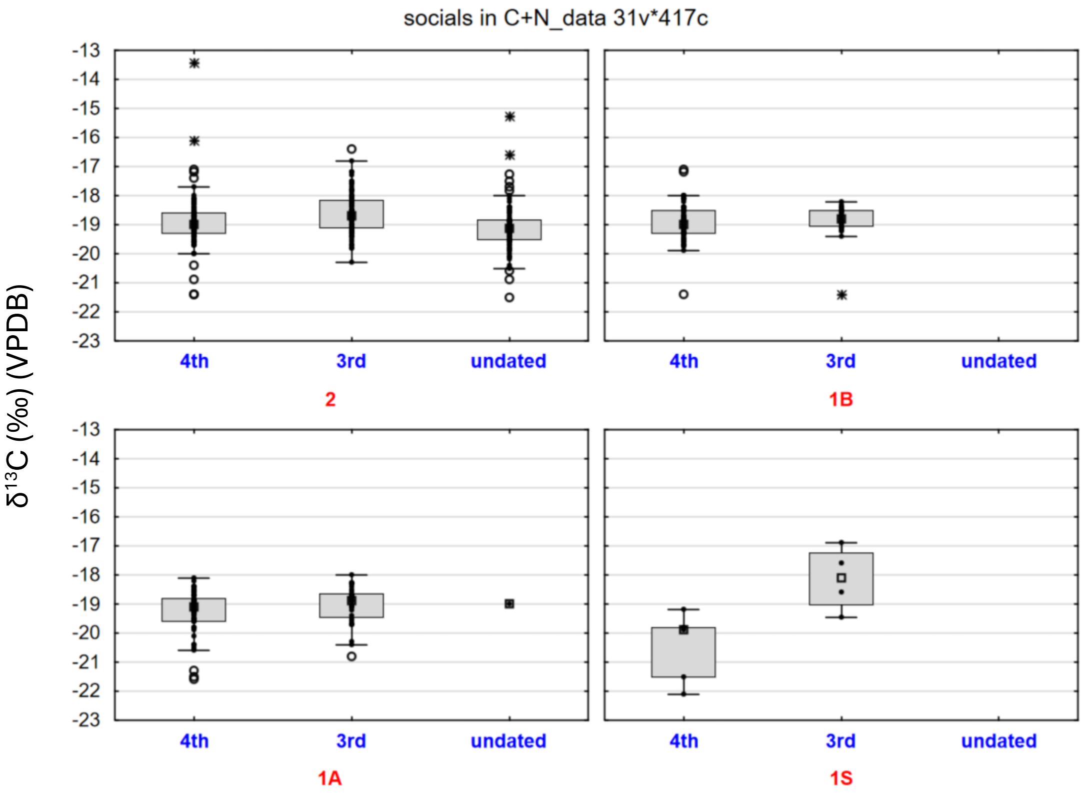

Figure S21. Social group categories compared individually within centuries.

Depend.: $\delta^{13}C$ (VPDB)	Multiple Comparisons p values (2-tailed); $\delta^{13}C$ (VPDB) (**4th century BC**)<br>Independent (grouping) variable: social group<br>Kruskal-Wallis test: H (3, N= 198) =13.93613 p =.0030			
	2 (R:108.33)	1B (R:105.39)	1A (R:85.627)	1S (R:25.500)
2		1.000000	0.157878	0.010208
1B	1.000000		0.420877	0.016452
1A	0.157878	0.420877		0.150883
1S	0.010208	0.016452	0.150883	

Depend.: $\delta^{13}C$ (VPDB)	Multiple Comparisons p values (2-tailed); $\delta^{13}C$ (VPDB) (**3rd century BC**)<br>Independent (grouping) variable: social group<br>Kruskal-Wallis test: H (3, N= 142) =7.814140 p =.0500			
	2 (R:77.994)	1B (R:67.464)	1A (R:56.344)	1S (R:94.375)
2		1.000000	0.073051	1.000000
1B	1.000000		1.000000	1.000000
1A	0.073051	1.000000		0.487685
1S	1.000000	1.000000	0.487685	

Table S23. Kruskal-Wallis ANOVA test of the social group categories according to centuries. First table is for the 4th century BC, second for 3rd century BC.

However, the limited number of samples in the category 1S suggests that this group may represent an anomaly, both statistically and perhaps also within the society itself. Also, the large number of samples from group 2 within the "undated" category calls for caution in interpreting the observed pattern. For these reasons, the subsequent analysis focused solely on the three most common social groups (1A, 1B, and 2), excluding the group 1S, and the undated samples.

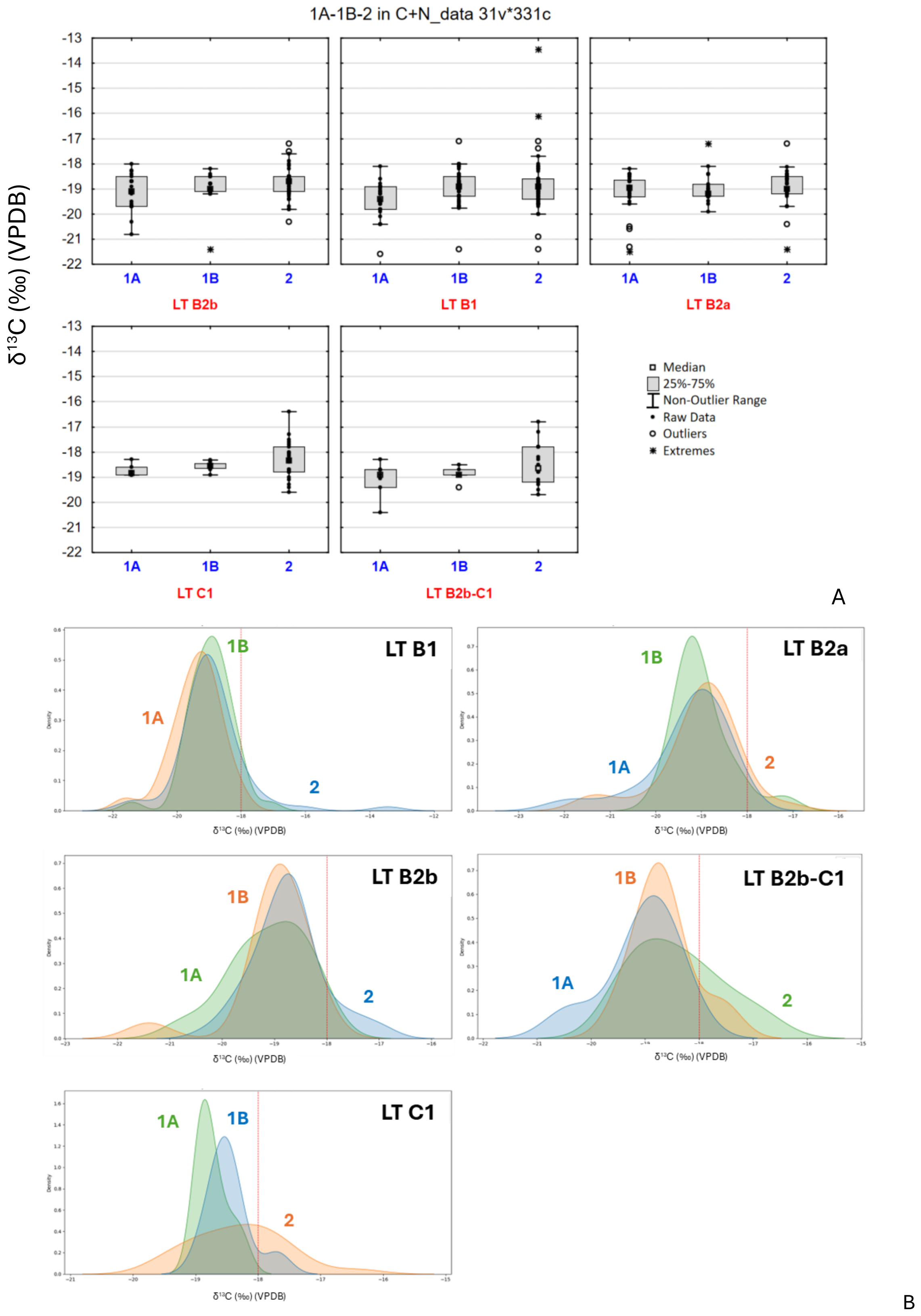

Figure S22. Social group categories (except for the group 1S) compared according to relative chronological phases. A – Box plots. The plots do not follow chronological order (correct: LT B1, LT B2a, LT B2b, LT B2b-C1, LT C1). B – kernel density plots.

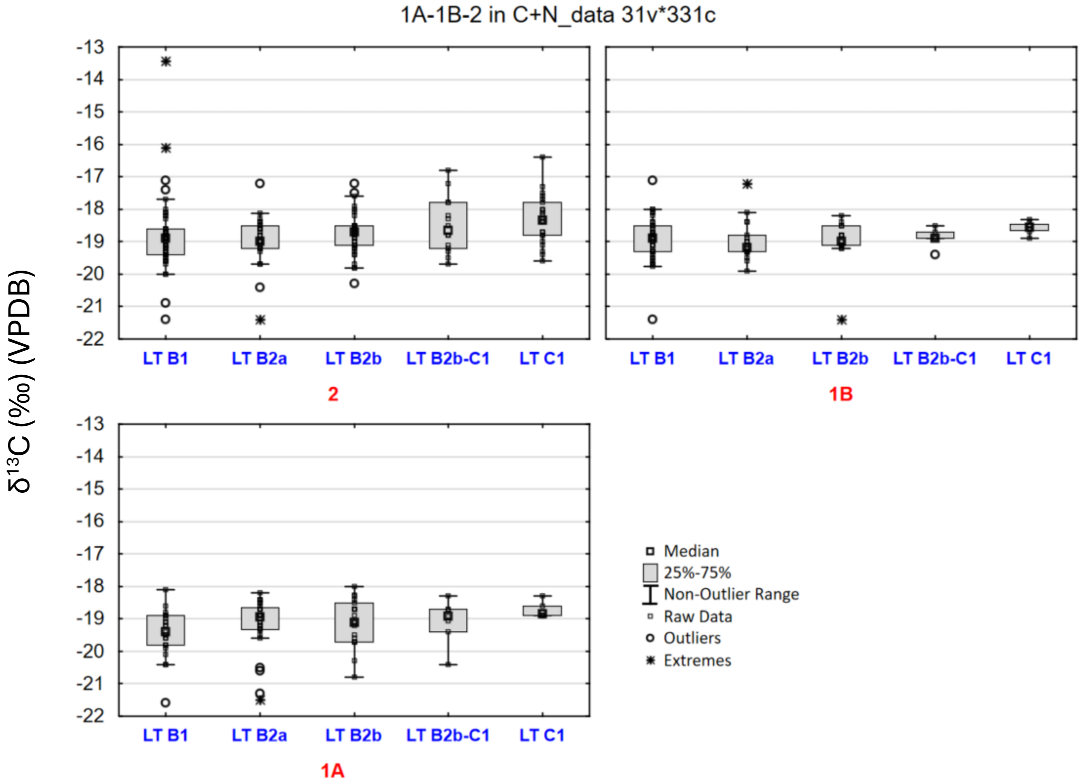

Figure S23. Social group categories compared individually within relative chronological phases.

From the results (Fig. S22, S23; Table S24, S25), it is evident that all social groups show a temporal development in their respective carbon values. Despite the overall increase observed in groups 1A and 1B, both consistently remain below the –18‰ threshold throughout the entire period. Group 2, by contrast, displays outlying values above –18‰ already in the earliest phases. Most notably, this group exhibits a systemic shift from phase LT B2b–C1 onwards, with values exceeding –17‰; the difference between the earliest and latest phases is statistically significant (Table S24). The other two groups, in contrast, show only a gradual and moderate increase, with a shift apparent in the final phase LT C1, though this was not statistically significant. The differences in carbon values between the groups, especially in phases LT B2b–C1 and LT C1, are clearly discernible (Fig. S22, S23).

	Multiple Comparisons p values (2-tailed); δ^{13}C (VPDB) (**"1A" group**)<br>Independent (grouping) variable: Dating<br>Kruskal-Wallis test: H (4, N= 83) =7.978291 p =.0924				
Depend.: δ^{13}C (VPDB)	LT B2b (R:44.237)	LT B1 (R:31.674)	LT B2a (R:44.250)	LT C1 (R:59.417)	LT B2b-C1 (R:45.929)
LT B2b		0.927244	1.000000	1.000000	1.000000
LT B1	0.927244		0.637358	0.120478	1.000000
LT B2a	1.000000	0.637358		1.000000	1.000000
LT C1	1.000000	0.120478	1.000000		1.000000
LT B2b-C1	1.000000	1.000000	1.000000	1.000000	

Depend.: $\delta^{13}C$ (VPDB)	Multiple Comparisons p values (2-tailed); $\delta^{13}C$ (VPDB) (**"1B" group**)<br>Independent (grouping) variable: Dating<br>Kruskal-Wallis test: H (4, N= 88) =7.193395 p =.1260				
	LT B2b (R:45.133)	LT B1 (R:43.720)	LT B2a (R:36.526)	LT C1 (R:65.188)	LT B2b-C1 (R:46.200)
LT B2b		1.000000	1.000000	0.729694	1.000000
LT B1	1.000000		1.000000	0.296958	1.000000
LT B2a	1.000000	1.000000		0.077706	1.000000
LT C1	0.729694	0.296958	0.077706		1.000000
LT B2b-C1	1.000000	1.000000	1.000000	1.000000	

Depend.: $\delta^{13}C$ (VPDB)	Multiple Comparisons p values (2-tailed); $\delta^{13}C$ (VPDB) (**"2" group**)<br>Independent (grouping) variable: Dating<br>Kruskal-Wallis test: H (4, N= 160) =11.88514 p =.0182				
	LT B2b (R:83.214)	LT B1 (R:68.655)	LT B2a (R:73.759)	LT C1 (R:106.18)	LT B2b-C1 (R:91.536)
LT B2b		1.000000	1.000000	0.596254	1.000000
LT B1	1.000000		1.000000	0.013237	0.989952
LT B2a	1.000000	1.000000		0.148316	1.000000
LT C1	0.596254	0.013237	0.148316		1.000000
LT B2b-C1	1.000000	0.989952	1.000000	1.000000	

Table S24. Kruskal-Wallis ANOVA test of the social group categories according to individual chronological phases. First table is for the group 1A, second for the group 1B, and the third is for the group 2.

variable	**social group=1A**<br>Mann-Whitney U Test (w/ continuity correction)<br>By variable century								
	Rank Sum	Rank Sum	U	Z	p-value	Z	p-value	Valid N	Valid N
$\delta^{13}C$ (VPDB)	1518.500	1967.500	641.5000	1.627947	0.103537	1.631125	0.102865	32	51

variable	**social group=1B**<br>Mann-Whitney U Test (w/ continuity correction)<br>By variable century								
	Rank Sum	Rank Sum	U	Z	p-value	Z	p-value	Valid N	Valid N
$\delta^{13}C$ (VPDB)	1429.500	2486.500	656.5000	1.639427	0.101125	1.642518	0.100484	28	60

variable	**social group=2**<br>Mann-Whitney U Test (w/ continuity correction)<br>By variable century								
	Rank Sum	Rank Sum	U	Z	p-value	Z	p-value	Valid N	Valid N
$\delta^{13}C$ (VPDB)	7112.500	5767.500	2364.500	2.843600	0.004461	2.846090	0.004426	78	82

Table S25. Mann-Whitney U test of the social group categories according to centuries. First table is for the group 1A, second for the group 1B, and the third is for the group 2.

References

Bujna, J. (1982). Spiegelung der Sozialstrukturen auf latènezeitlichen Gräberfeldern im Karpatenbecken. *Památky archeologické*, *77*, 312–431.

Buonasera, T., Eerkens, J., de Flamingh, A., Engbring, L., Yip, J., Li, H., Haas, R., DiGiuseppe, D., Grant, D., Salemi, M., Nijmeh, C., Arellano, M., Leventhal, A., Phinney, B., Byrd, B. F., Malhi, R. S., & Parker, G. (2020). A comparison of proteomic, genomic, and osteological methods of archaeological sex estimation. *Scientific Reports*, *10*(1), 11897. https://doi.org/10.1038/s41598-020-68550-w

Evans, T. L. (2004). *Quantitative Identities: A Statistical Summary and Analysis of Iron Age Cemeteries in North-Eastern France 600–130 BC*. BAR Publishing, Oxford. https://doi.org/10.30861/9781841715919

Gaydarska, B., Rebay-Salisbury, K., Valiente, P. R., Fries, J. E., Hofmann, D., Augereau, A., Chapman, J., Mina, M., Pape, E., Ialongo, N., Nordholz, D., Bickle, P., Haughton, M., Robb, J., & Harris, O. (2023). To Gender or not To Gender? Exploring Gender Variations through Time and Space. *European Journal of Archaeology*, *26*(3), 271–298. https://doi.org/10.1017/eaa.2022.51

Hodson, F. R. (1968). *The La Tène cemetery at Münsingen-Rain. Catalogue and relative chronology*.

Holodňák, P. (1988). Keltská pohřebiště ve středním Poohří. *Památky archeologické*, *79*, 38–105.

Kertés, S. (2025). *Potential Use of Data Analyses and Machine Learning in the Interpretation of Provenance through Stable Strontium Isotopes in the La Tène Period* [Diplomová práce]. Univerzita Karlova, Filozofická fakulta, Ústav pro archeologii.

Krämer, W. (1985). *Die Grabfunfe von Manching und die Latènezeitlichen Flachgräber in Südbayern. Band 9*.

Moghaddam, N., Müller, F., Hafner, A., & Lösch, S. (2016). Social stratigraphy in Late Iron Age Switzerland: Stable carbon, nitrogen and sulphur isotope analysis of human remains from Münsingen. *Archaeological and Anthropological Sciences*, *8*, 149–160. https://doi.org/10.1007/s12520-014-0221-4

Patterson, N., & al, et. (2022). Large-scale migration into Britain during the Middle to Late Bronze Age. *Nature*, *601*, 588–594. https://doi.org/10.1038/s41586-021-04287-4

Ramsl, P. C. (2002). Das eisenzeitliche Gräberfeld von Pottenbrunn. Forschungsansätze zu wirtschaftlichen Grundlagen und sozialen Strukturen der latènezeitlichen Bevölkerung des Traisentales, Niederösterreich. *Fundberichte aus Österreich-Materialhefte, Reihe A, Heft 11*.

Ramsl, P. C. (2018). War and violence at La Tène period in Middle Europe (La Tène period cemeteries at Eastern Austria, Slovakia and Moravia). In E. Nemeth (Ed.), *Violence in Prehistory and Antiquity / Die Gewalt in der Vorgeschichte und im Altertum: Roč. VI* (s. 117–139).

Schönfelder, M. (2009). Archäologische Untersuchungen zur Elite in der keltischen Gesellschaft – eine Zwischenbilanz. In M. Egg & D. Quast (Ed.), *Aufstieg und Untergang. Zwischenbilanz des Forschungsschwerpunktes »Studien zu Genese und Struktur von Eliten in vor- und Frühgeschichtlichen Gesellschaften«* (Roč. 82, s. 59–78). Verlag des Römisch-Germanischen Zentralmuseums.

Venclová, N., Drda, P., Michálek, J., Militký, J., Salač, V., Sankot, P., & Vokolek, V. (Ed.). (2013). *Prehistory of Bohemia 6. The Late Iron Age – The La Tène Period*. Archeologický ústav AV ČR, Praha, v.v.i.

Vytlačil, Z., Danielisová, A., Velemínský, P., Blažek, J., & Drtikolová Kaupová, S. (2024). Dietary changes seen through the isotope analysis of the La Tène burial site of Prosmyky (Bohemia, 4th-3rd century BCE). *Archaeological and Anthropological Sciences*, *16*, 86. https://doi.org/10.1007/s12520-024-01994-7

Waldhauser, J. & others. (1978). *Das keltische Gräberfeld bei Jenišův Újezd in Böhmen, I–II*.

Waldhauser, J. & others. (1987). Keltische Gräberfelder in Böhmen. *Bericht der Römisch-Germanischen Kommission*, *68*, 25–179.
